# Direct Cα-heteroarylation of structurally diverse ethers *via* a mild *N*-hydroxysuccinimide mediated cross-dehydrogenative coupling reaction†
†Electronic supplementary information (ESI) available: Tables S1–S8, Schemes S1–S10, Fig. S1–S8, experimental procedures and characterization for all of the new compounds. See DOI: 10.1039/c6sc05697k


**DOI:** 10.1039/c6sc05697k

**Published:** 2017-03-24

**Authors:** Shihui Liu, Aoxia Liu, Yongqiang Zhang, Wei Wang

**Affiliations:** a Shanghai Key Laboratory of New Drug Design , School of Pharmacy , State Key Laboratory of Bioengineering Reactor , East China University of Science and Technology , 130 Mei-long Road , Shanghai 200237 , China . Email: yongqiangzhang@ecust.edu.cn ; Email: wwang@unm.edu; b Department of Chemistry and Chemical Biology , University of New Mexico , Albuquerque , NM 87131-0001 , USA

## Abstract

An important challenge in the Cα-heteroarylation of ethers is the requirement of a large excess amount of ethers (that are used as solvents in many cases) to achieve effective transformations. This drawback has significantly restricted the Cα-heteroarylation of ethers to the use of simple and easily accessible ether substrates. To overcome this limitation, a new, efficient, *N*-hydroxysuccinimide (NHS) mediated, mild and metal-free CDC strategy for the direct Cα-heteroarylation of diverse ethers has been developed. Different to our previous benzaldehyde mediated photoredox Cα-heteroarylation, we have identified NHS as a new and efficient mediator without using light. A distinct non-photoredox engaged hydrogen-atom-transfer (HAT) mechanism that used a nitrogen-centered radical cation produced from NHS is initially revealed. Notably, only 5–10 equivalents of ethers as coupling partners are used, which allows for structurally diverse and complex ethers to engage in this process, to create highly medicinally relevant Cα-heteroarylated ethers. Furthermore, more structurally diverse heterocyclics can serve as reactants for this process.

## Abstract 2

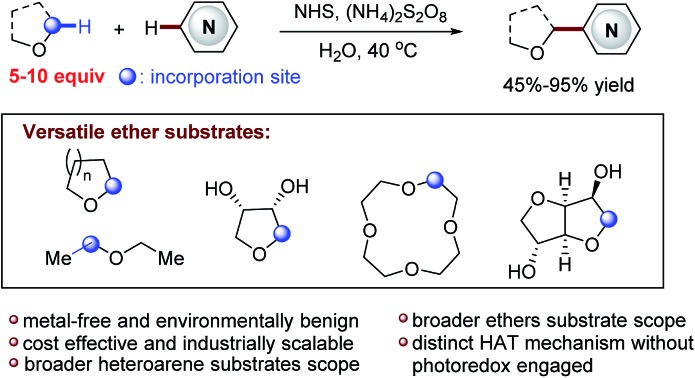
A new, efficient, *N*-hydroxysuccinimide (NHS) mediated, mild and metal-free CDC strategy for the direct Cα-heteroarylation of diverse ethers has been developed.

## Introduction

**Fig. 1:**
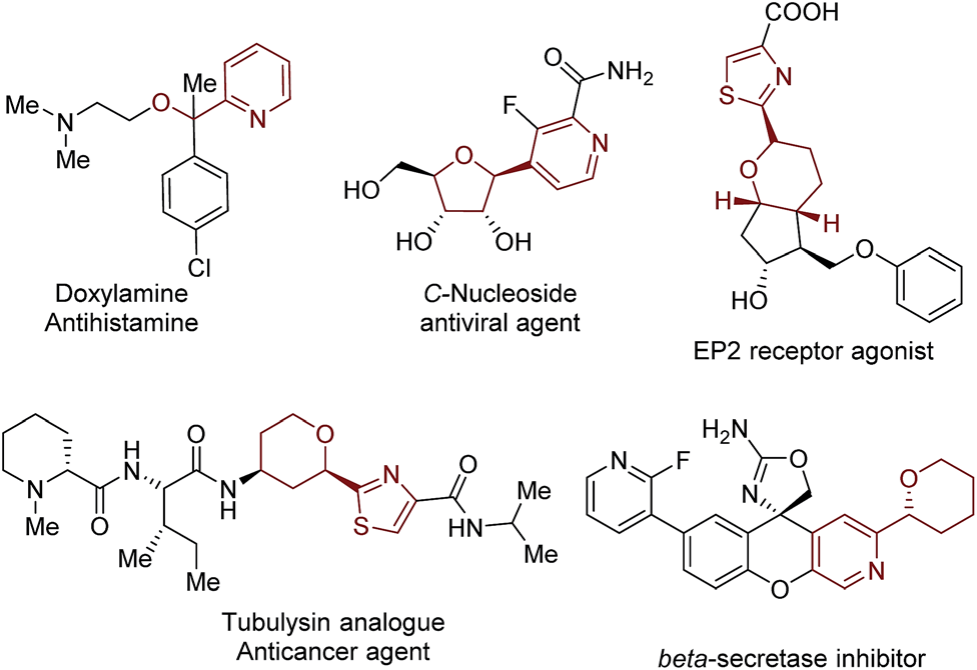
Selected bioactive compounds that contain heteroaryl α-ether functionality.

**Fig. 2:**
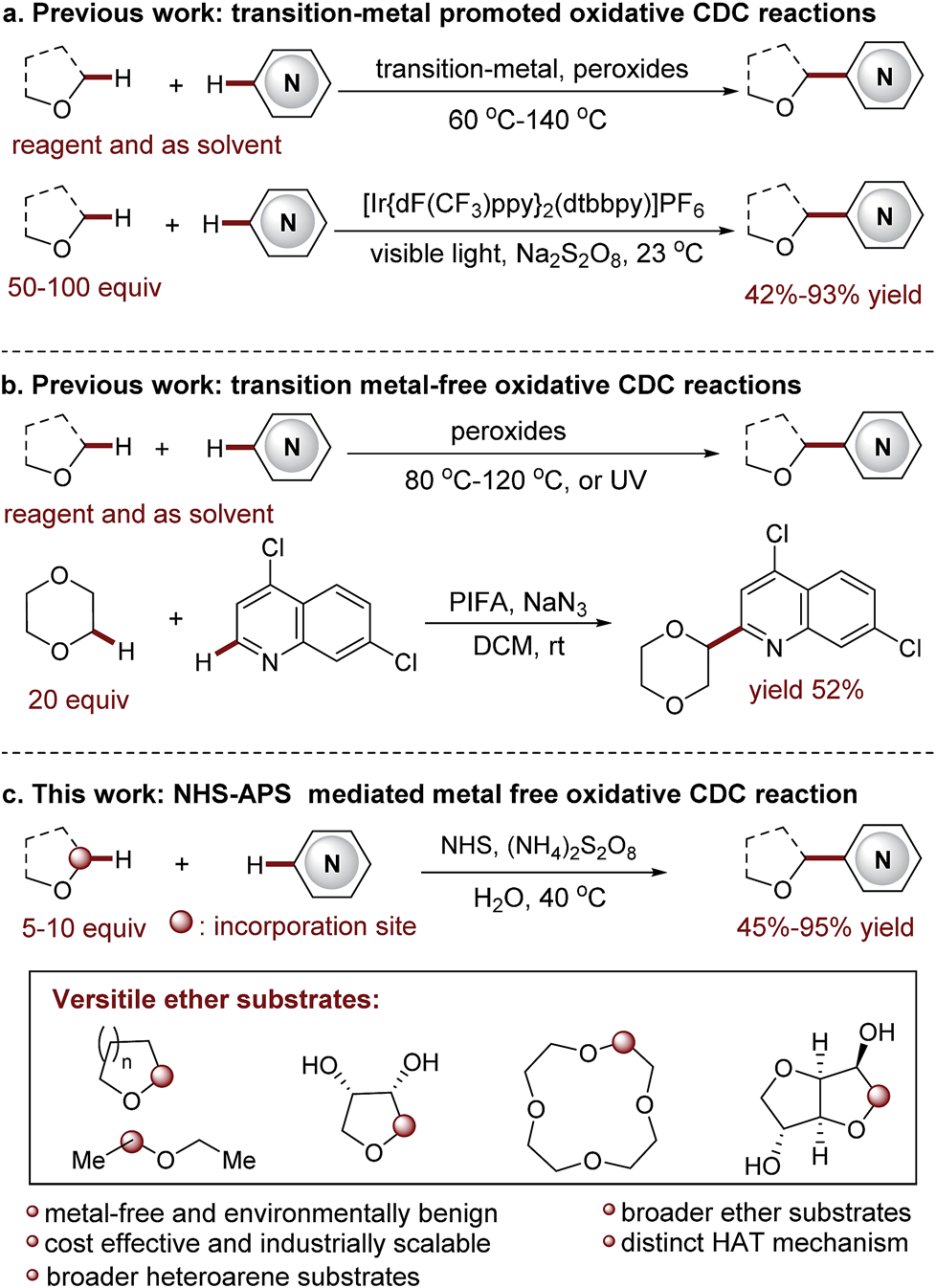
Cα-heteroarylation of ethers *via* an oxidative CDC reaction.

Functionalized acyclic and cyclic ethers, particularly those with 5- and 6-cyclic structural motifs, are featured in numerous natural products and synthetic pharmaceuticals with a broad spectrum of biological properties (see the representative examples shown in Fig. 1).^1^ Synthetic methods that enable the direct incorporation of these units into complex heteroaromatic structures without requiring any prefunctionalization represent a particularly appealing approach to produce valuable molecular architectures.^2^ Significant efforts have been made towards this goal. Among them, the oxidative cross-dehydrogenative coupling (CDC) reaction^3^ has become a viable strategy for the selective Cα-heteroarylation of ethers *via* a radical coupling process. These processes are generally carried out using transition metals as catalysts, such as Sc(OTf)_3_,4*a* Cu(OTf)_2_,4*b* FeF_2_,4*c* Ni(acac)_2_,4*d* NiF_2_,4*d* and CoCO_3_,4*e* in the presence of explosive peroxides under harsh reaction conditions (such as high reaction temperatures (60–140 °C)) (Fig. 2a). Such reaction conditions cause significant safety concerns. Furthermore, the intrinsic issue is that a large excess of ether substrates (often used as the solvent) is necessary. This drawback restricts the Cα-heteroarylation of ethers to the use of simple and easily accessible ethers, which creates a great application challenge in the synthesis of medicinally relevant and highly functionalized ether structures. Recently, a visible light driven CDC reaction for the Cα-heteroarylation of ethers under mild conditions was elegantly realized by MacMillan and co-workers. However, a transition metal based photocatalyst and a large excess of ethers (50–100 equiv.) were still required in this process.^5^

Metal-free CDC reactions have received increasing attention due to their environmentally-benign nature (Fig. 2b). In these approaches, oxidants such as K_2_S_2_O_8_, *t*-butyl hydroperoxide (TBHP), benzoyl peroxide (BPO), and butylperoxybenzoate (DTBP) are used to initiate the processes. However, a large excess of ethers was still required for effective transformations, but products were delivered with moderate yields.^6^ An improved protocol developed by Burgmann *et al.* employed 20 equiv. of dioxane as the coupling partner (Fig. 2b),^7^ but only one example was demonstrated with only moderate yield (52%), and explosive NaN_3_ was used in this process.

Clearly, the most critical, formidable and challenging issue faced in this methodology is to reduce the amount of ether used. This will streamline the Cα-heteroarylation of ethers with the capacity of employing expensive, complex ether substrates. Towards this end, we wish to report an unprecedented *N*-hydroxysuccinimide (NHS) mediated mild CDC reaction (Fig. 2c). A distinct nitrogen-centered radical cation mediated hydrogen-atom-transfer (HAT) mechanism without using metal activation is initially proposed. As demonstrated, this metal free protocol enables the use of only 5–10 equiv. of ether. Therefore, a wide array of ethers, including many complex ones, are used for the first time in the CDC processes to deliver fascinating medicinally relevant coupling products. Furthermore, broader heteroarene substrates are proved to be effective reactants.

## Results and discussion

Recently we developed a photoredox process for the Cα-heteroarylation of amides and ethers (both were used as co-solvents).^8^ In this study, we observed the insightful promotion effect of amides in an (NH_4_)_2_S_2_O_8_ (ammonium persulfate, APS) mediated oxidative CDC reaction. This interesting phenomenon triggered us to study its role in the process. We believed that this study might lead to not only understanding the reaction mechanism but also developing a more efficient and broadly applicable methodology for the Cα-heteroarylation of diverse ethers.

Firstly we conducted an experiment to verify the promotion effect of amides. A simple acetamide was initially employed in the APS initiated CDC reaction of tetrahydrofuran (THF) with isoquinoline (Table 1). We found that the presence of acetamide (2 equiv.) could lead to Cα regioselective product **3a** in high yield (entry 2, 77% yield) at 40 °C. In contrast, without it, only a 14% yield was obtained (entry 1). The outcomes of these studies clearly showed the promotion effect of acetamide.

**Table 1:** Studies of the promotion effect of amides, their derivatives, amines and amine alcohols in the Cα-heteroarylation of tetrahydrofuran with isoquinoline and the optimization of the reaction conditions^*a*^

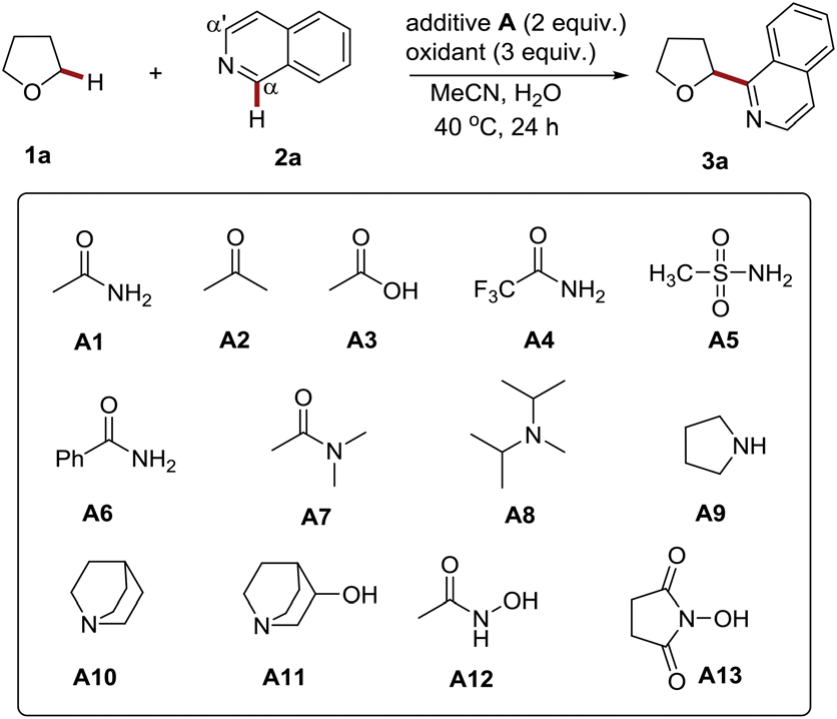				
Entry	Oxidant	Additive	**1a** (equiv.)	Yield^*b*^
1	(NH_4_)_2_S_2_O_8_	—	20	14%
2	(NH_4_)_2_S_2_O_8_	**A1**	20	77%
3	(NH_4_)_2_S_2_O_8_	**A2**	20	15%
4	(NH_4_)_2_S_2_O_8_	**A3**	20	24%
5	(NH_4_)_2_S_2_O_8_	**A4**	20	50%
6	(NH_4_)_2_S_2_O_8_	**A5**	20	53%
7	(NH_4_)_2_S_2_O_8_	**A6**	20	55%
8	(NH_4_)_2_S_2_O_8_	**A7**	20	70%
9	(NH_4_)_2_S_2_O_8_	**A8**	20	7%
10	(NH_4_)_2_S_2_O_8_	**A9**	20	15%
11	(NH_4_)_2_S_2_O_8_	**A10**	20	80%
12	(NH_4_)_2_S_2_O_8_	**A11**	20	82%
13	(NH_4_)_2_S_2_O_8_	**A12**	20	5%
14	(NH_4_)_2_S_2_O_8_	**A13**	20	88%
15	(NH_4_)_2_S_2_O_8_	**A13**	20	42%^*c*^
16	(NH_4_)_2_S_2_O_8_	**A13**	20	82%^*d*^
17	(NH_4_)_2_S_2_O_8_	**A13**	20	75%^*e*^
18	(NH_4_)_2_S_2_O_8_	**A13**	10	82%
19	(NH_4_)_2_S_2_O_8_	**A13**	10	80%^*f*^
20	(NH_4_)_2_S_2_O_8_	**A13**	5	60%^*f*^
21	(NH_4_)_2_S_2_O_8_	**A13**	5	67%^*f*^ ^,^^*g*^

^*a*^Conditions employed **1a** (2.5–10.0 mmol), **2a** (0.5 mmol), oxidant (1.5 mmol), additive (1.0 mmol), 40 °C, 24 h, and a solvent mixture (1.5 mL, MeCN : H_2_O = 1 : 1), unless otherwise noted.

^*b*^The yields were determined using ^1^H NMR with CH_2_Br_2_ as an internal standard.

^*c*^Performed with (NH_4_)_2_S_2_O_8_ (1 equiv.).

^*d*^Performed with **A13** (1 equiv.).

^*e*^Performed with **A13** (0.5 equiv.).

^*f*^Water (1.5 mL) was used as the solvent.

^*g*^Performed on a 1 g scale.

Having proved the acetamide promotion effect, we then focused on the optimization of the reaction conditions to improve the reaction efficiency. The screening of the reaction temperature and the amount of acetamide did not improve the yield (see Tables S1 and S2†). Then a variety of amides, including methanesulfonamide, and other additives were probed. It was found that the amine moiety of acetamide plays an essential role in promoting these processes (entries 2–4). The replacement of the acetyl group with stronger electron-withdrawing groups, such as trifluoroacetyl, benzoyl, and methylsulfonyl groups, resulted in the decrease of the reaction yields (entries 5–7, 50–55% yield), while the introduction of two methyl groups into acetamide (*N*,*N*-dimethylacetamide, DMA), delivered a comparable yield, accompanied with a trace amount of the α-heteroarylated product of DMA (α to nitrogen atom) (entry 8, 70% yield). It is well known that electron-rich amines, such as morpholine and piperidine, tend to be oxidized by APS, and thereby generate nitrogen-centered radicals to initiate the polymerization of olefins.^9^ It suggests that amides might also work similarly to initiate radical-based reactions *via* a redox process with APS.^10^ This may be the reason for its promotion effect. To the best of our knowledge, such an effect has not been explored for organic reactions. In addition to amides, amine additives with higher reducibility, including diisopropylethylamine (DIEA) and pyrrolidine, were probed. Nevertheless, the reaction was completely inhibited and most of the isoquinoline was recovered (entries 9–10, 7–15% yield). The redox reaction of a highly reductive amine with APS, followed by hydrogen atom transfer (HAT) at the α-position, might account for the quenching of this radical-based process (Scheme S1†).^11^ Accordingly, quinuclidine amines with a rigid structure and stronger Cα–H bond^12^ were tested. Indeed, they displayed more pronounced promotion effects (entries 11–12, 80–82% yield), which further supports the proposed quenching mechanism. A further screening study was performed by employing *N*-hydroxyacetamide as the additive, considering that the hydroxyl group can efficiently increase the electron density and reducibility of its amine group *via* a conjugate effect. It might also be more stable towards sulfate radical mediated HAT due to a polar effect.^13^ Nevertheless, the reaction was also completely suppressed with a lower yield (entry 13, 5% yield). The relatively lower BDE (bond dissociation energy) value of the O–H bond may contribute to this observation. Thus, *N*-hydroxysuccinimide (NHS) with a stronger O–H bond was then probed.^14^ To our delight, it displayed the most improved reaction efficiency (entry 14, 88% yield). The control experiments further confirmed the efficient promotion effect of NHS to the radical based CDC reactions initiated by APS (see Table S3†). With NHS as the additive, APS was proven to be the best oxidant for this process (see Table S4†). Lowering the amount of APS and NHS resulted in decreased yields (entries 15–17, 42–82% yield). Notably, decreasing the amount of THF to 10 equiv. only led to a slight drop in the yield (entry 18, 82% yield). Furthermore, when the reaction was performed in water, an excellent yield was also achieved (entry 19, 80% yield). Further reducing the amount of THF to 5 equiv. gave a moderate yield (60%, entry 20). Different to the traditional Minisci reaction (the direct open-shell addition of an alkyl radical to heteroarenes),^15^ the addition of acid additives, such as Sc(OTf)_3_4*a* and TFA,6*b* does not improve the process (see entries 2–3, Table S5†). The *in situ* generation of a strong acid (often sulfuric acid) might be expected to explain this result. The use of TBAB6*c* and even the replacement of NHS with quinuclidin-3-ol (**A11**) are also not able to improve this transformation (see entries 4–5, Table S5†). It should be noted that the reaction can be adapted to a gram scale with only 5 equiv. of THF (entry 21, 67% isolated yield), highlighting its potential utility in an industrial setting.

Having identified the optimal reaction conditions for the Cα-heteroarylation of ethers (Table 1, entry 19), we next focused on examining the scope of the heteroarene components (Table 2). A variety of structurally diverse electron-deficient heteroarenes readily coupled with THF in moderate to good yields, taking place at the most electrophilic site. For isoquinoline substrates bearing both electron-withdrawing and electron-donating groups, the reactions proceeded well (**3b–3j**, 72–95% yields) with excellent Cα selectivity. Employing 5 equiv. of THF could deliver the products, albeit with slightly lower yields (**3b–3j**, 43–65% yields). Bromine, ester, and chlorine groups were well tolerated, providing handles for further functionalization. Quinolines also displayed high reactivity towards this process to give products **3k–3o** in good yields (78–88%). By employing the standard procedure, unsubstituted quinolines gave two products, a C4 mono-substituted product and bis-substituted product **3k** in a ratio of 1 : 0.6 (data not shown here). It appeared that the C4 position is more active than the C2 position. The extension of the reaction time to 72 h led to a C2 and C4 bis-substituted product (**3k**, 75%). Blocking either position enabled the production of exclusive regioisomers **3l–3o**, respectively, in high yields. Pyridines also worked smoothly to give the corresponding products **3p–3q** in good yields (56–62%). Complete C6 selectivity was observed for 3-cyanopyridine (**3p**), while 4-cyanopyridine led to the prevalent C2 mono-substituted product **3q** accompanied with the bis-adduct in a 10 : 1 ratio. It is worth noting that efficient methods to directly couple azoles with ethers *via* a radical based CDC pathway are rather limited due to their reduced electrophilic nature.4b,4c,4e,6a Therefore, azole substrates were investigated with our new methodology. To our delight, benzothiazoles also functioned as suitable substrates, providing the products **3r–3s** in reasonable yields (48–56%). However, benzimidazole was not amenable to this protocol, and the reaction was completely suppressed. The same outcome was seen for 1-Cbz-benzimidazole, accompanied with the removal of the Cbz group. Interestingly, 1-methyl-benzimidazole reacted well to give the desired product **3u**. Finally, carboline, a widely used chemical scaffold in drug discovery,^16^ was also applicable to this protocol, providing the product in excellent yield (**3w**, 91% yield).

**Table 2:** NHS mediated Cα-heteroarylation of tetrahydrofuran with diverse heteroarenes^*a*^

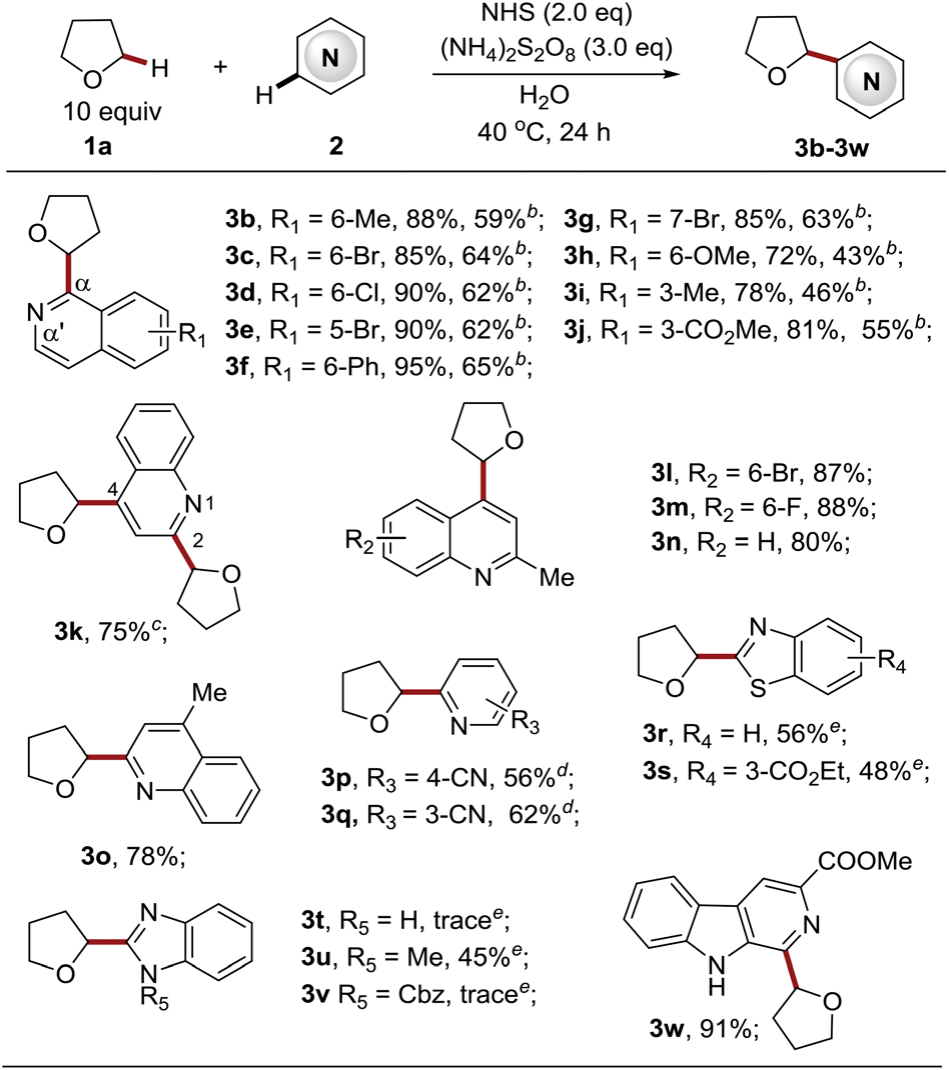

^*a*^See the general procedure for the experimental details unless otherwise noted. The isolated yield was reported for each reaction.

^*b*^Performed with 5 equiv. of THF.

^*c*^The reaction time was extended to 72 h.

^*d*^ **3p**, mono-substituted product (C-2) : bis-substituted product (C-2 and C-6) = 10 : 1 (r.r.); **3q**, C-6 product only (r.r.); the regiomeric ratio (r.r.) was determined using ^1^H NMR spectroscopy.

^*e*^20 equiv. of THF were employed.

Having demonstrated the capacity of a variety of heteroaromatics that are capable of engaging in the process, we next probed the generality and limitations of the ether components by employing isoquinoline as the coupling partner (Table 3). It should be noted that a mixture of MeCN : H_2_O = 1 : 1, v/v was employed as the solvent for ether substrates with poor water solubility. Notably, besides tetrahydrofuran (THF), pharmaceutically relevant and widely used cyclic ethers, including tetrahydropyran (THP), 1,4-dioxane, 1,3-dioxane, 1,3-dioxolane and 12-crown-4, were probed and were shown to be compatible with the process by delivering the products in good to excellent yields (**4a–4e**, 75–90% yields). It is worth noting that only 5 equiv. of 1,4-dioxane and highly costly 12-crown-4 were used in the coupling reactions to deliver high yields of **4d** and **4e**. We observed that the C-4 position of 1,3-dioxane is slightly favored over the C-2 position (**4c**, C-2 : C-4 = 1 : 1.6). However, excellent C-2 regioselectivity was observed for 1,3-dioxolane (**4d**, C-2 : C-4 = 8 : 1), thus providing a facile approach to install an aldehyde group in heteroarenes *via* acid-catalyzed hydrolysis.^17^ Acyclic diethyl ether (b.p. 35 °C) still maintained a good yield (**4f**, 65% yield) in spite of its high volatility. Substituted cyclic ethers, such as 2-methyltetrahydrofuran and tetrahydrofurfuryl acetate, were also well tolerated (**4g–4h**, 70–82% yield). The less-hindered C-5 position is slightly favored over the C-2 position for 2-methyltetrahydrofuran (**4g**, C-5 : C-2 = 2 : 1), while complete C-5 control was achieved in the presence of three available α-oxy positions containing C–H bonds in tetrahydrofurfuryl acetate (**4h**). Both of the C-5 heteroarylated products exhibited moderate diastereoselectivity. Six-membered tetrahydropyran bearing a methyl carboxylate also worked smoothly with excellent diastereoselectivity (**4i**, 62% yield; >20 : 1 d.r.). Interestingly, 2,2-dimethyl-1,3-dioxolane, as a “masked” glycol, exclusively resulted in Cα-heteroarylated glycol in good yield (**4j**, 74% yield), while glycol itself was not compatible with the reaction (data not shown here). Nevertheless, it is worth noting that unprotected multi-hydroxyl substituted cyclic ethers could efficiently couple with heteroarenes at the α-position to give C-nucleoside molecules **4k–4n** in one step, which are ‘privileged’ structures with anti-cancer and anti-virus activities.^18^ It should be noted that multiple steps and harsh reaction conditions were usually required for their synthesis.^19^ It appears that the free hydroxyl groups can be tolerated without protection. The presence of hydroxyl at the ether ring plays a role in governing the regioselectivity (**4l**, C-2 : C-5 = 2 : 1). It also contributes to the improvement of diastereoselectivity (**4l**, C-2 product, 5 : 1 d.r.; **4l**, C-5 product, >20 : 1 d.r.; **4m**, >20 : 1 d.r.; and **4n**, 10 : 1 d.r.). Finally, it is intriguing to find that the use of complex isosorbide, a commonly used scaffold in drug discovery,^20^ led to the heteroarylation of α-methylene in good yield and with excellent diastereoselectivity (**4o**, 65% yield, 20 : 1 d.r.).

**Table 3:** NHS mediated Cα-heteroarylation of diverse ethers with isoquinoline^*a*^

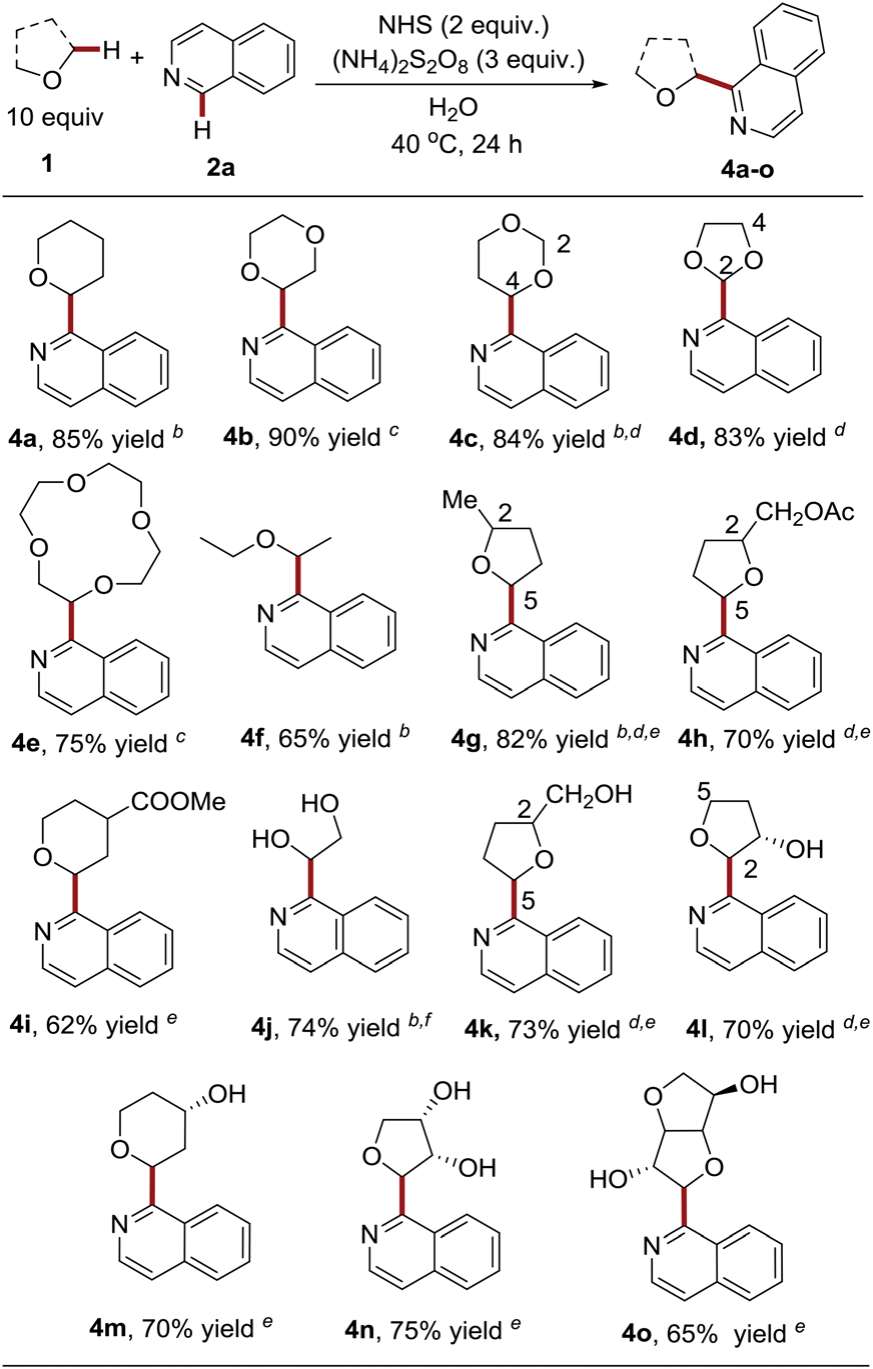

^*a*^See the general procedure for the experimental details unless otherwise noted. The isolated yield was reported for each reaction.

^*b*^The solvent mixture (MeCN : H_2_O = 1 : 1) was employed as the solvent.

^*c*^Performed with 5 equiv. of ethers.

^*d*^Regiomeric ratio (r.r.) determined using ^1^H NMR spectroscopy; **4c**, C-2 : C-4 = 1 : 1.6 (r.r.); **4d**, C-2 : C-4 = 8 : 1 (r.r.); **4g**, C-5 : C-2 = 2 : 1 (r.r); **4h** and **4k**, C-5 product only (r.r); and **4l**, C-2 : C-5 = 2 : 1 (r.r.).

^*e*^Diastereomeric ratio (d.r.) determined using^1^H NMR spectroscopy. **4g**, 1.7 : 1 d.r.; **4h**, 2.2 : 1 d.r.; **4i**, >20 : 1 d.r.; **4k**, 2 : 1 d.r.; **4l**, 5 : 1 d.r. (C-2 product); **4l**, >20 : 1 d.r. (C-5 product); **4m**, >20 : 1 d.r.; **4n**, 10 : 1 d.r.; and **4o**, 20 : 1 d.r.

^*f*^2,2-Dimethyl-1,3-dioxolane was employed as the coupling partner and **4j** was produced *via* the deprotection promoted by an *in situ* generated acid.

## Mechanism

Having established the scope of the method, we performed a study on the reaction mechanism. The formation of product **3a** was suppressed in the presence of TEMPO, a radical quencher (Scheme S3†), again suggesting that radical processes are involved in this oxidative CDC reaction. Furthermore, we observed a kinetic isotopic effect (KIE = 3.0, intermolecular competition reaction, and KIE = 2.2, two parallel reactions) when using [^2^H_8_] tetrahydrofuran and **2a** (see the ESI for details†). Therefore, the hydrogen atom abstraction (HAT) from the Cα position of tetrahydrofuran is the rate-limiting step in this oxidative cross-coupling reaction.

Considering the promotion effect of additives with reductive amine groups in the above coupling reaction (Tables 1 and S3†), the subsequent experiments were accordingly designed to investigate the redox reaction of amide and amine additives with APS. Firstly, quinuclidin-3-ol and quinuclidine, with electron rich amine groups, easily suffer from APS mediated oxidation to generate active radical species according to ^1^H NMR and EPR studies (electron paramagnetic resonance) (Scheme S6, Fig. S2 and S3†). It is of note that hydroxyl radical mediated HAT might not be involved in this process due to the excellent selectivity of tetrahydrofuran towards Cα-heteroarylation in the above reaction.^21^ A HAT process mediated by a sulfate radical was also excluded due to the higher reaction efficiency of our methodology in the Cα-heteroarylation of ethers.4b,^5^,6c–e Therefore, it is reasonably believed that the quinuclidine based cation radical formed in this redox reaction provides a more efficient approach to mediate HAT of the Cα-heteroarylation of ethers.^22^ Moreover, amide additives, including acetamide and NHS, might also be involved in the redox reaction with APS *via* the formation of reactive intermediates with nitrogen–oxygen bonds, thereafter providing another powerful HAT approach *via* the generation of amide based nitrogen-centered cation radicals (Scheme S7, Fig. S4 and S5†),^23^,^24^ which was further supported by the study of the redox reaction of *N*-phenylacetamide with APS (Scheme S8 and Fig. S6†). It should be noted that the neutral oxygen radical of the NHS mediated HAT process was ruled out according to the EPR experiment and control reactions (Scheme S10 and Fig. S7†). The occurrence of a HAT mechanism mediated by a nitrogen centered radical was further supported by the results of a preliminary kinetic study of the oxidative CDC reaction promoted by quinuclidin-3-ol and NHS (Table S8 and Fig. S8†).

**Scheme 1:**
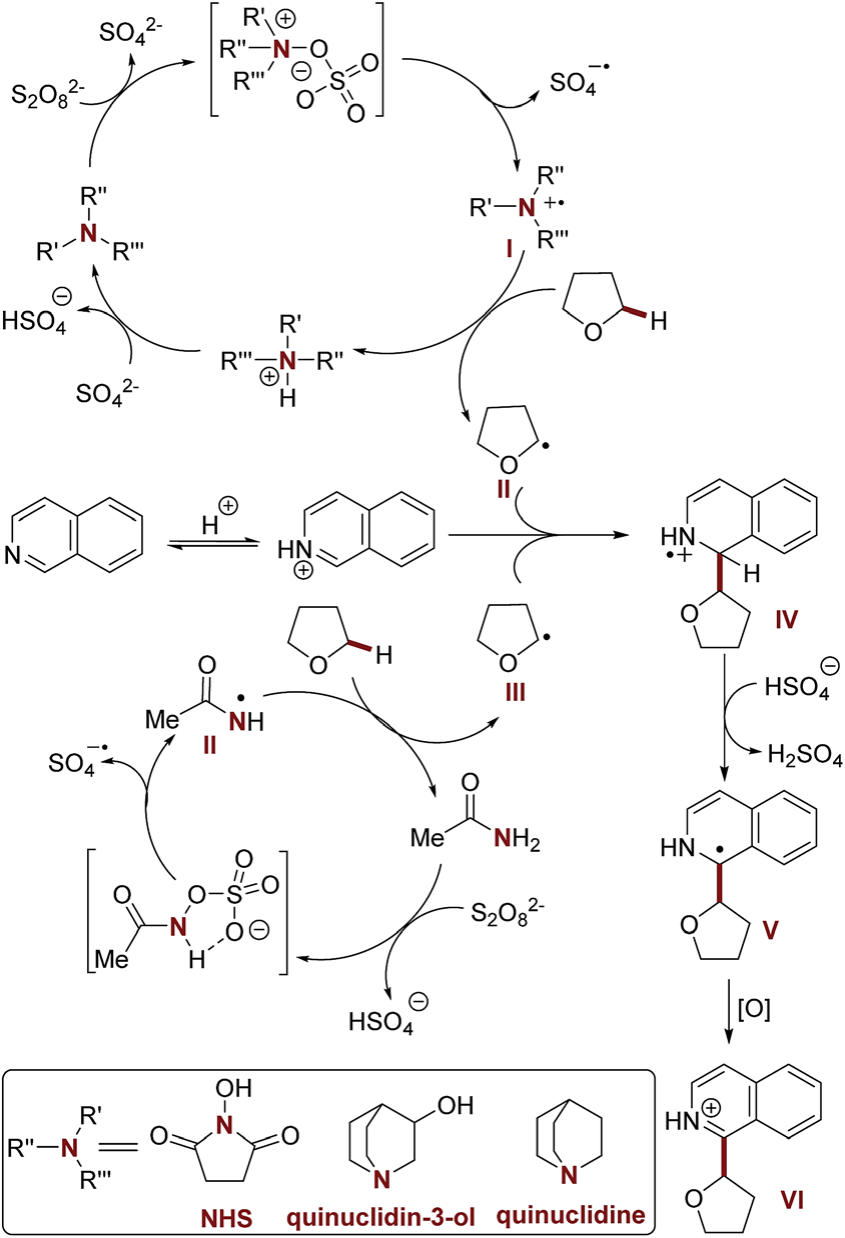
Proposed mechanism.

Based on these studies, a plausible reaction mechanism is proposed (Scheme 1). The redox reaction of the additive with APS provides the nitrogen-centered cation radical (**I** and **II**), which serves as an efficient mediator for HAT towards the Cα-heteroarylation of tetrahydrofuran. The resulting α-oxy radical (**III**) undergoes nucleophilic addition with the protonated isoquinoline at the C-1 position, followed by deprotonation and oxidation to provide the desired product **3a**. Further validation of the mechanism is still ongoing in our laboratory.

## Conclusion

In summary, a NHS mediated, mild and metal-free CDC reaction has been developed. The process was uncovered through the studies of the observed promotion effect of amides and amines in an APS mediated radical coupling reaction. The procedure is environmentally benign, cost effective and industrially scalable. Notably, the process is highly efficient by using 5.0–10.0 equiv. of ethers. Therefore, diverse ethers, particularly complex and expensive structures, become possible substrates. Moreover, the mild reaction conditions allow for various electron-deficient heteroarenes to affect the regioselectivity of the process at more electrophilic positions with a broad functional group tolerance. The promotion effects of NHS and other additives, such as acetamide, quinuclidine, and quinuclidin-3-ol, were studied. A distinct HAT mechanism mediated by a nitrogen-centered radical cation is revealed. Further investigation of the mechanism of the process and the application of the reactivity for new organic transformations is being pursued in our laboratories.

## Supplementary Material

Supplementary information
