# Supplementary material for: Direct Cα-heteroarylation of structurally diverse ethers via a mild N-hydroxysuccinimide mediated cross-dehydrogenative coupling reaction
Source: Chem Sci. 2017 Mar 24;8(5):4044–50. doi: 10.1039/c6sc05697k (PMC6094177; doi:10.1039/c6sc05697k)

Electronic Supplementary Material (ESI) for Chemical Science.
This journal is © The Royal Society of Chemistry 2017

Direct Cα-Heteroarylation of Structurally Diverse Ethers *via* a Mild *N*-Hydroxy Succinimide Mediated Cross-Dehydrogenative Coupling Reaction

Shihui Liu,[a] Aoxia Liu,[a] Yongqiang Zhang,[a,] and Wei Wang[a,b,]**

[a] School of Pharmacy, East China University of Science and Technology, Shanghai 200237, P. R. China

[b] Department of Chemistry and Chemical Biology, University of New Mexico, Albuquerque, NM 87131-0001, USA

*Correspondence to: Professor Wei Wang (E-mail: wwang@unm.edu) and Dr. Yongqiang zhang (Email: yongqiangzhang@ecust.edu.cn)

Table of Contents

The screening of reaction conditions……………………………………………......S2
The proposed quenching mechanism of amine additives…………………………....S4
The comparison of Cα-heteroarylation methodologies of ether..…………….……....S4
The radical quenching experiment…………………………………………….……..S4
Kinetic isotope effect experiments……………………………………………….......S5
The study of the redox reaction of quinuclidine structure with APS……………..…..S6
The study of the redox reaction of amide additives with APS……………………......S8
The validation of neutral oxygen radical of NHS……………………………….……S12
The preliminary kinetic study of the reaction…………………………………………S14
Experiment Procedures and Product Characterization.……………………................S15
References…………………..…………………...............................……………………….S34
Spectral Data for the Products…………....…...…………………………….........……S35

The screening of reaction conditions

Table S1 The screening of reaction temperature [a]

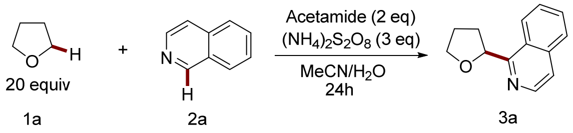

Entry	Tem (°C)	Yield [b]
1	25	14%
2	30	46%
3	35	65%
4	40	77%
5	45	74%

[a] Conditions employed **1a** (10 mmol), **2a** (0.5 mmol), $(NH_4)_2S_2O_8$ (1.5 mmol), acetamide (1.0 mmol), 24 h, and a solvent mixture (1.5 mL, MeCN:H_2O = 1:1), unless otherwise noted; [b] Yields were determined by ^{1}H NMR using CH_2Br_2 as an internal standard.

Table S2 The screening of the amount of acetamide [a]

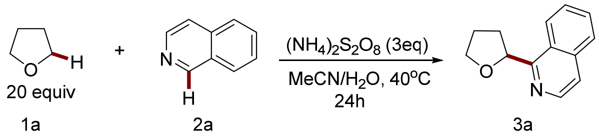

Entry	Acetamide (equiv)	Yield [b]
1	8	78%
2	5	80%
3	2	77%
4	1	68%
5	0.5	58%

[a] Conditions employed **1a** (10 mmol), **2a** (0.5 mmol), $(NH_4)_2S_2O_8$ (1.5 mmol), 40 °C, 24 h, and a solvent mixture (1.5 mL, MeCN:H_2O = 1:1), unless otherwise noted; [b] Yields were determined by ^{1}H NMR using CH_2Br_2 as an internal standard.

Table S3 Further validation of the promotion effect of NHS in the reaction [a]

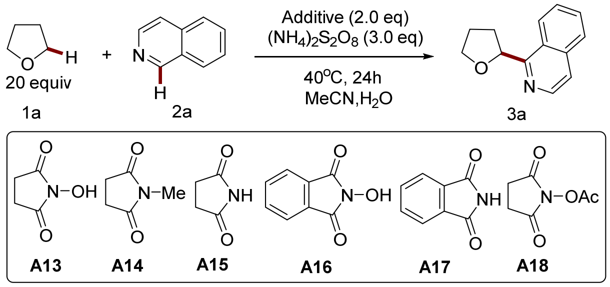

Entry	Additive	Yield [b]
12	A13	88%
13	A14	62%
14	A15	71%
15	A16	70%
16	A17	65%
17	A18	48%

[a] Conditions employed **1a** (10.0 mmol), **2a** (0.5 mmol), $(NH_4)_2S_2O_8$ (1.5 mmol), additive (1.0 mmol), 40 °C, 24 h, and a solvent mixture (1.5 mL, MeCN:H_2O = 1:1), unless otherwise noted; [b] Yields were determined by ^{1}H NMR using CH_2Br_2 as an internal standard.

Table S4 The screening of the oxidant with NHS as the additive [a]

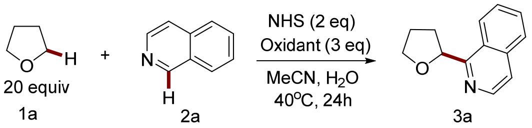

Entry	Oxidant	Yield(%) [b]
1	$(NH_4)_2S_2O_8$	88%
2	BPO	n.d [c]
3	TBHP	n.d [c]
4	$Na_2S_2O_8$	53%
5	$K_2S_2O_8$	45%

[a] Conditions employed **1a** (10.0 mmol), **2a** (0.5 mmol), oxidant (1.5 mmol), NHS (1.0 mmol), 40 °C, 24 h, and a solvent mixture (1.5 mL, MeCN:H_2O = 1:1), unless otherwise noted; [b] Yields were determined by ^{1}H NMR using CH_2Br_2 as an internal standard; [c] not detected.

Table S5 Further reaction condition optimization with 5 equivalent of THF as coupling partner [a]

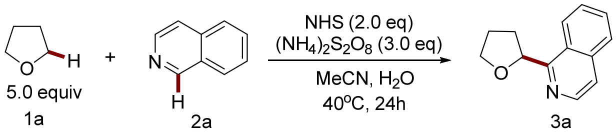

Entry	Additive	Yield(%) [b]
1	-	60%
2	TBAB (30 mol%) [c]	63%
3	$Sc(OTf)_3$ (10 mol%)	60%
4	TFA (1 equiv) [c]	60%
5	-	30% [d]

[a] Conditions employed **1a** (2.5 mmol), **2a** (0.5 mmol), $(NH_4)_2S_2O_8$ (1.5 mmol), NHS (1.0 mmol), 40 °C, 24 h, and a solvent mixture (1.5 mL, MeCN:H_2O = 1:1), unless otherwise noted; [b] Yields were determined by ^{1}H NMR using CH_2Br_2 as an internal standard; [c] TBAB: tetrabutylammonium bromide, TFA: trifluoroacetic acid; [d] quinuclidin-3-ol (**A11**) was used instead.

Table S6 Further reaction condition optimization with benzothiazole as coupling partner [a]

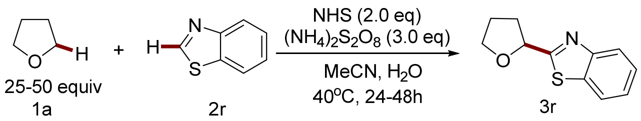

Entry	THF (equiv)	Time (h)	Yield [b]
1	20	24	56%
2	20	48	57%
3	50	24	60%
4	20	24	52% [c]

[a] Conditions employed **1a** (10-25 mmol), **2a** (0.5 mmol), $(NH_4)_2S_2O_8$ (1.5 mmol), NHS (1.0 mmol), 40 °C, 24-48 h, and a solvent mixture (1.5 mL, MeCN:H_2O = 1:1), unless otherwise noted; [b] Isolated yield was reported; [c] quinuclidin-3-ol (**A11**) was used instead.

The proposed quenching mechanism of amine additives

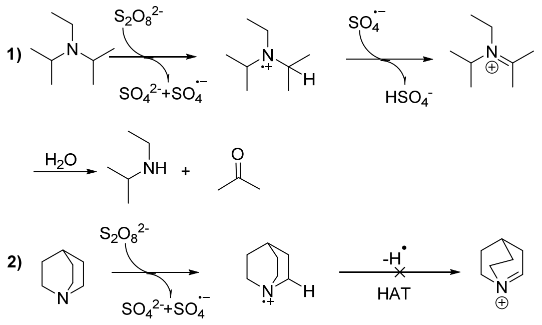

Scheme S1 Proposed quenching mechanism of DIEA in the reaction

The comparison of Cα-heteroarylation methodologies of ether

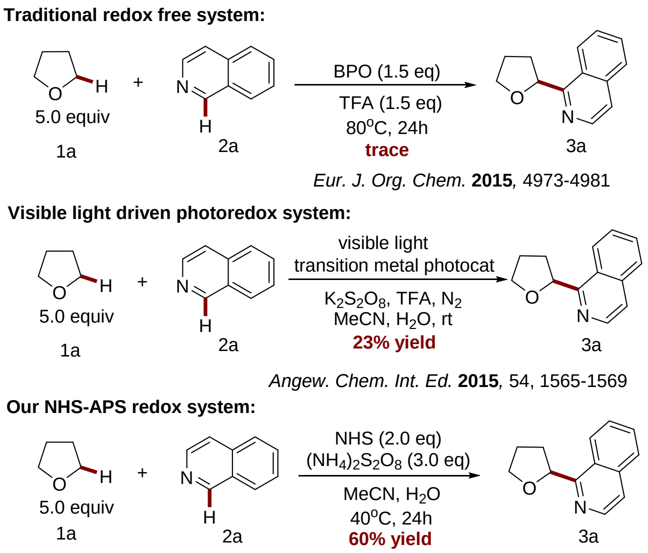

Scheme S2 The experimental result with three different reaction systems

The radical quenching experiment

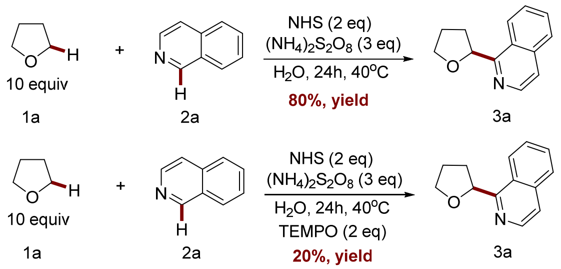

Scheme S3 The experiment to validate radical based mechanism

Kinetic Isotope Effect Experiments (KIE)[1]

The KIE value determined from an intermolecular competition reaction

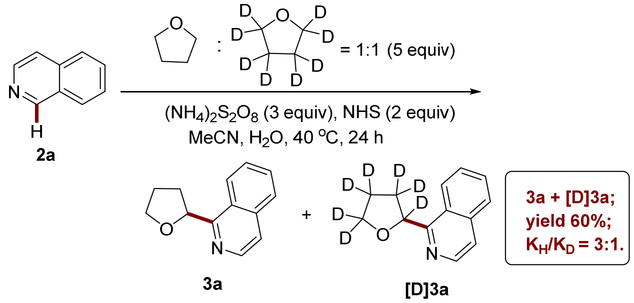

Scheme S4 Intermolecular competition KIE experiment

The KIE value determined from two parallel reactions

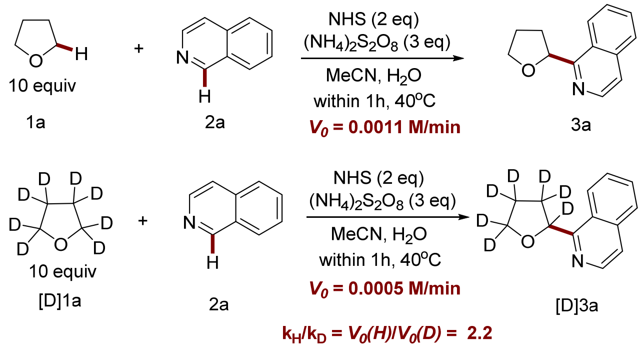

Scheme S5 KIE experiment employing two parallel reactions

Table S7 The initial rate determination of two parallel reactions employing THF and d^8-THF as coupling partner [a]

Ether	Time(min)	NMR Yield (%) [b]	[Product](M)	V_0
THF	10	5	0.01	0.0011M/min
	20	12	0.026	
	30	16	0.035	
	40	22	0.047	
	50	27	0.057	
	60	31	0.067	

Ether	Time(min)	NMR Yield (%)	[Product](M)	V_0
d^8-THF	10	2	0.004	0.0005M/min
	20	5	0.01	
	30	7	0.013	
	40	9	0.02	
	50	11	0.024	
	60	12	0.026	

[a] To a solution of THF(or THF-d_8) (5.00 mmol, 10.0 equiv.), isoquinoline (0.50 mmol, 1.0 equiv.) in 1.5 mL of CH_3CN/H_2O (1: 1) was added $(NH_4)_2S_2O_8$ (1.50 mmol, 3.0 equiv.), N-Hydroxy succinimide (1.00 mmol, 2.0 equiv.), the reaction mixture was heated at 40 °C; [b] Analyzed by

NMR spectroscopy for the formation of product with CH_2Br_2 as an internal standard.

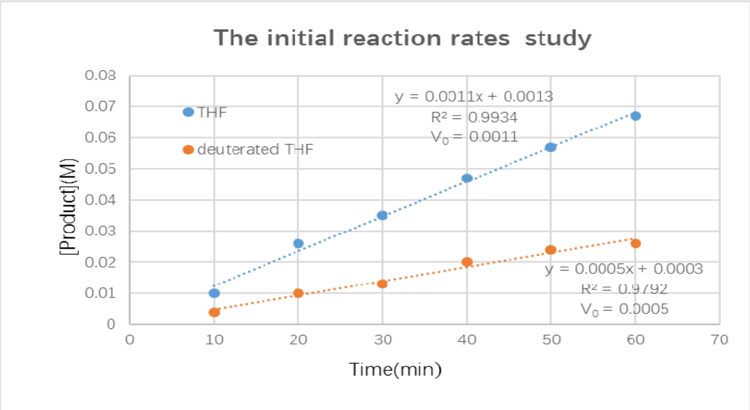

Fig. S1 The initial rate determination of two parallel reactions

The study of the redox reaction of quinuclidine structure with APS

Notes: The ^{1}H NMR spectra of quinuclidine and quinuclidin-3-ol in deuterated water were monitored after treating with APS. These two additives were totally protonated to produce the corresponding sulfate complexes even at 20 °C (Figure S2). It should be noted that the protonic acid, such as HSO_4^-, H_2SO_4, could not be generated via a heat-induced decomposition of APS or $Na_2S_2O_8$ in water at 40°C or 20°C.[2] Furthermore, the EPR data of the solution of APS in water was collected with 5,5-dimethyl-1-pyrroline N-oxide (DMPO) as free-radical spin-trapping agent. In the presence of quinuclidine, the signal of hydroxyl radical was detected,[3] while no distinct signal was observed without additive (Fig. S3). Based on these findings, we concluded that quinuclidine additives might function via the redox reaction with APS to generate the active radical species, such as sulfate radical, nitrogen based radical cation, and hydroxyl radical. The mechanism of the redox reaction of quinuclidine structure with persulfate was proposed accordingly (Scheme S6).[2]

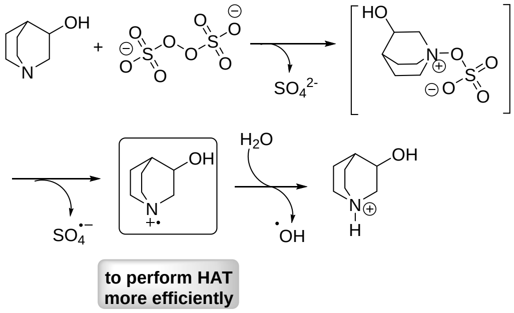

Scheme S6 The proposed redox reaction mechanism of quinuclidin-3-ol with APS

The ^{1}H NMR study of the redox reactions of quinuclidine structure with APS

General procedure: To a solution of quinuclidine or quinuclidin-3-ol (0.4 mmol, 1.0 equiv.) in D_2O (0.6 mL), was added $(NH_4)_2S_2O_8$ (0.6 mmol, 1.5 equiv.). The resulting

solution was stirred at 40 °C or 20 °C for 24 h, the ^{1}H NMR data of the reaction mixture was then collected accordingly.

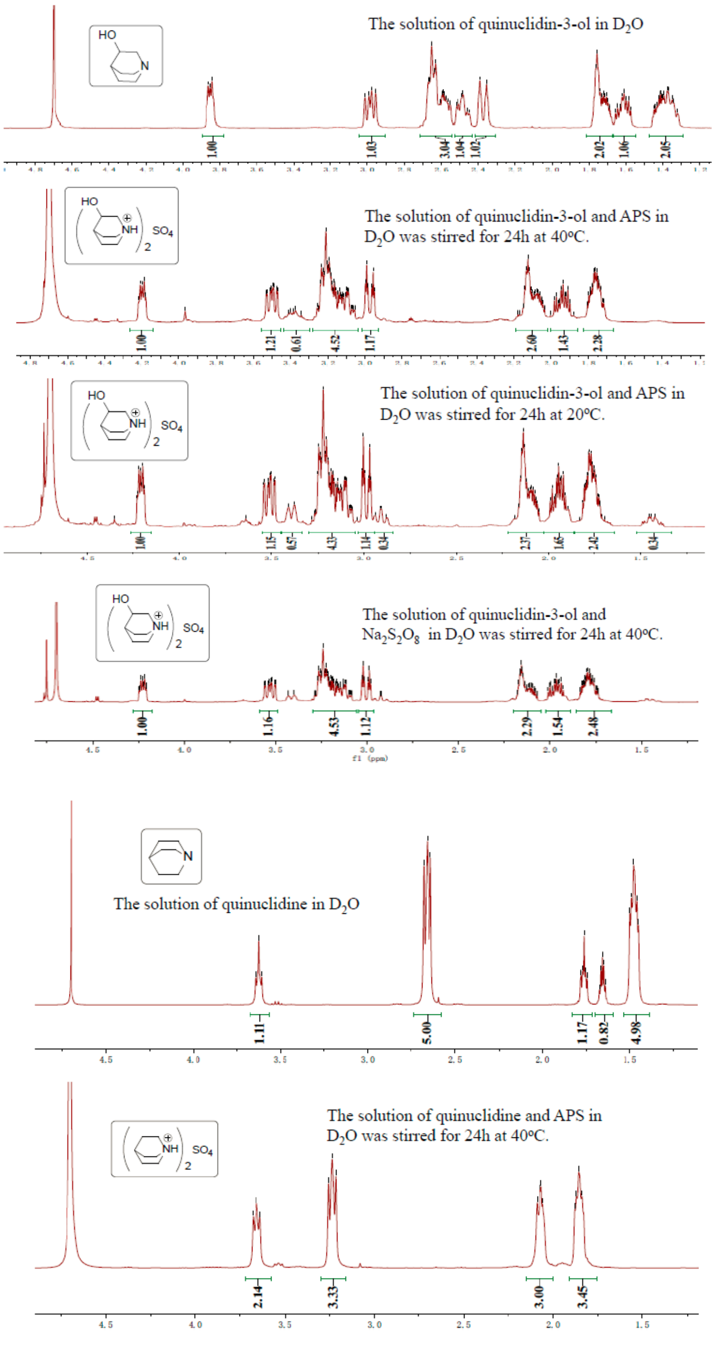

Fig. S2 The ^{1}H NMR spectra of the reaction solution of quinuclidin-3-ol or quinuclidine with APS

Electroparamagnetic resonance (EPR) study of the redox reactions of quinuclidine structure with APS

General procedure: the samples were prepared immediately before the acquisition, after tuning the ESR instrument parameters. The preparation is described as follows:

A solution of quinuclidine additives (0.05 mmol, 1 equiv.), DMPO (0.05 mmol, 1 equiv.) in H_2O (1.0 mL) was added to APS (0.05 mmol, 1 equiv.) in ESR tubes.

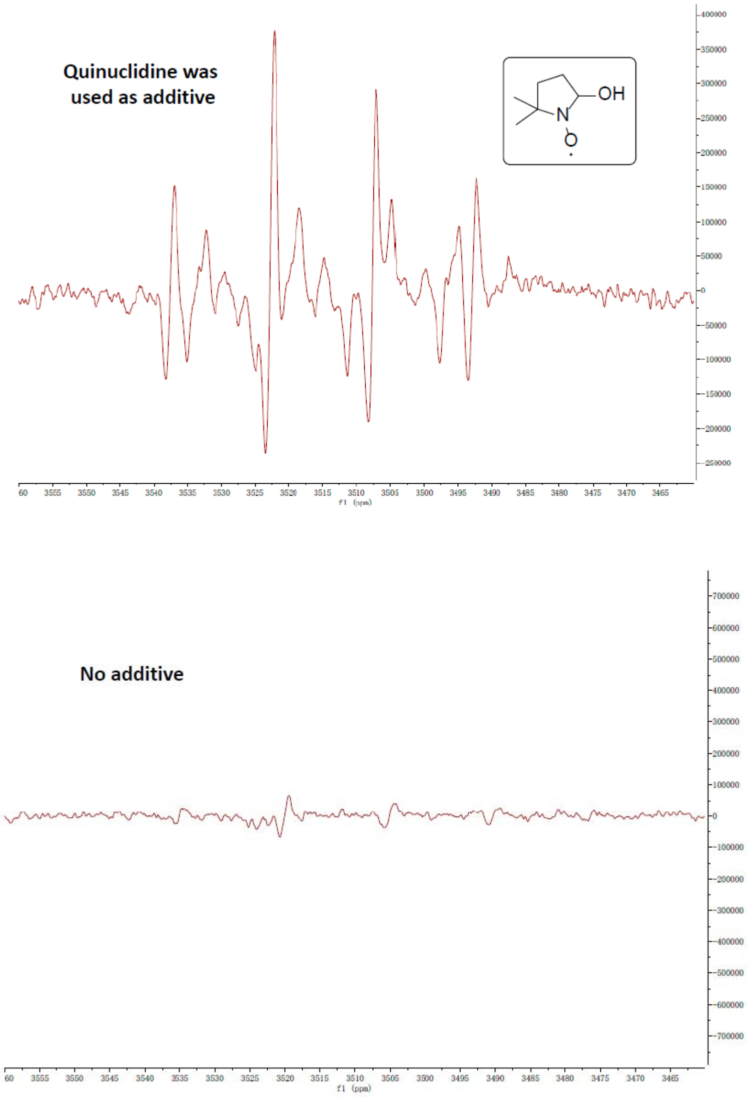

Fig. S3 The EPR spectra of the solution of APS in water

The study of the redox reaction of amide additives with APS

Notes: The ^1H NMR spectra of amide additives in deuterated water were monitored after treating with APS. Acetamide and NHS, as the additives to efficiently promote the reaction, displayed remarkable new ^1H NMR signals, while no distinct new ^1H NMR signals was detected for less efficient methanesulfonamide (Fig. S4). Moreover, in the presence of acetamide and NHS, the hydroxyl radical signal was also detected in the EPR experiment of the solution of APS in water with 5,5-dimethyl-1-pyrroline N-oxide (DMPO) as free-radical spin-trapping agent (Fig. S5). At last, APS mediated dimerization via a nitrogen-nitrogen bond, a typical reaction of nitrogen-concerted radial,[4] was observed by MS for *N*-phenylacetamide (Scheme S8 and Figure S6). Therefore, we envisioned that the redox intermediate with nitrogen-oxygen bond might be developed initially in this process, thereafter provides active radical species

to further mediate HAT process. The mechanism of the redox reaction was proposed (Scheme S7).

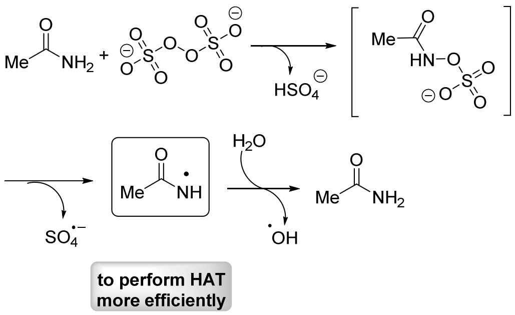

Scheme S7 Proposed redox reaction mechanism of acetamide with APS

General procedure: To a solution of amide additives (0.4 mmol, 1.0 equiv.) in D_2O (0.6 mL), was added $(NH_4)_2S_2O_8$ (0.6 mmol, 1.5 equiv.). The resulting solution was stirred at 40 °C or 20 °C for 24 h, the ^{1}H NMR data of the reaction mixture was then collected accordingly.

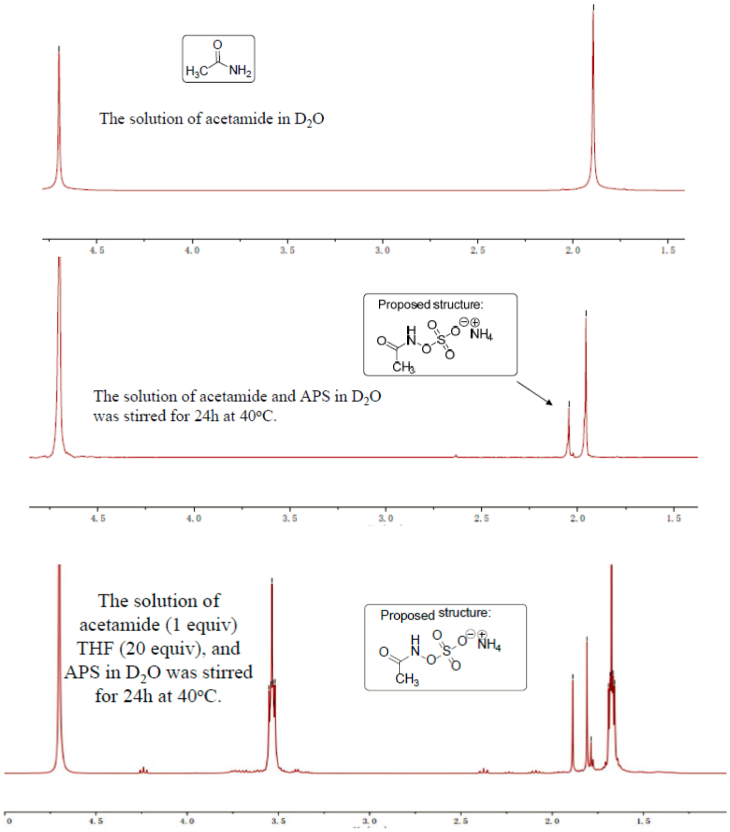

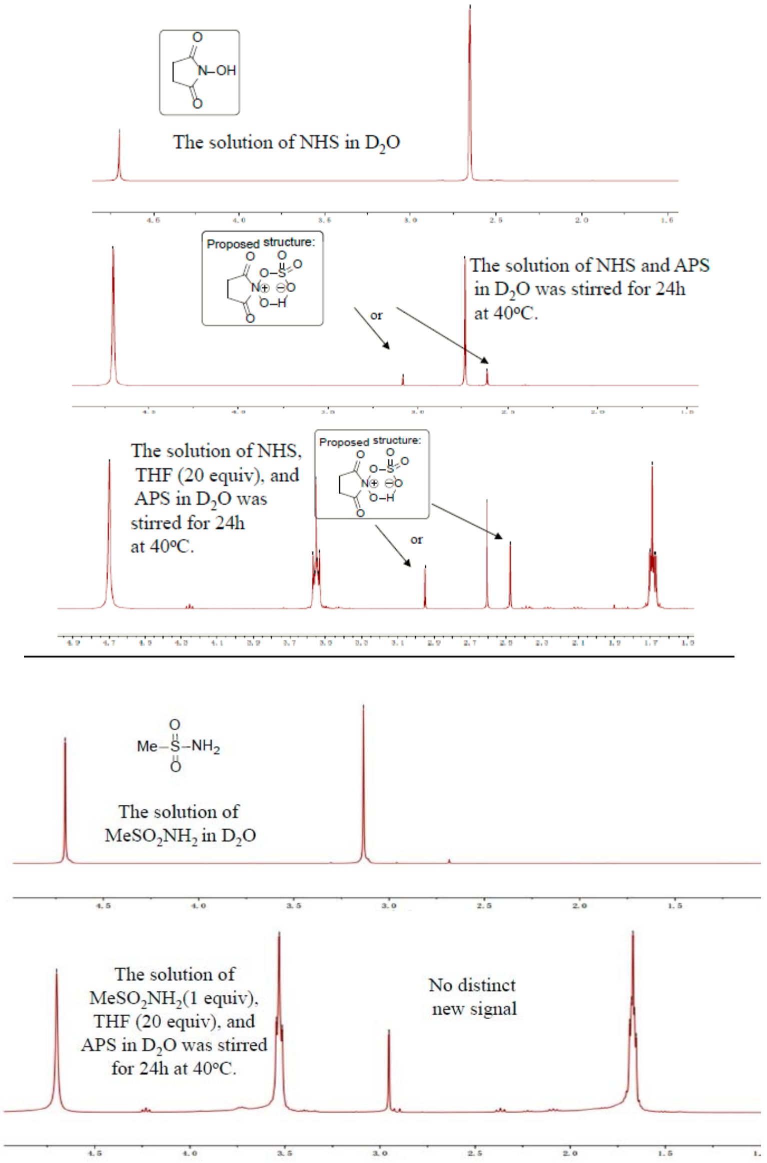

Fig. S4 The ^{1}H NMR spectra of the reaction solution of acetamide or NHS with APS

Electroparamagnetic resonance (EPR) study of the redox reactions of amide additive with APS

General procedure: the samples were prepared immediately before the acquisition, after tuning the ESR instrument parameters. The preparation is described as follows:

A solution of amide additive (0.05 mmol, 1 equiv.), DMPO (0.05 mmol, 1 equiv.) in H_2O (1.0 mL) was added to APS (0.05 mmol, 1 equiv.) in ESR tubes.

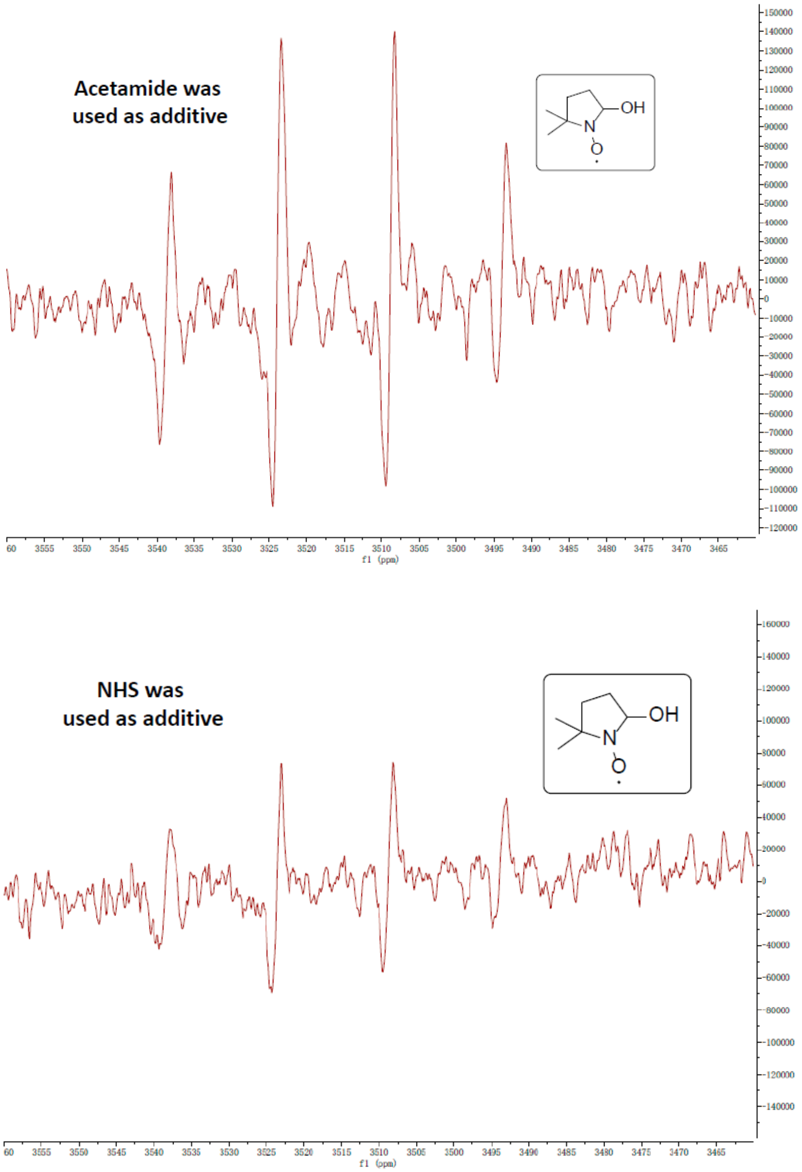

Fig. S5 The EPR spectra of the solution of APS in water with NHS and acetamide as additives

The MS study of the redox reaction of amide with APS

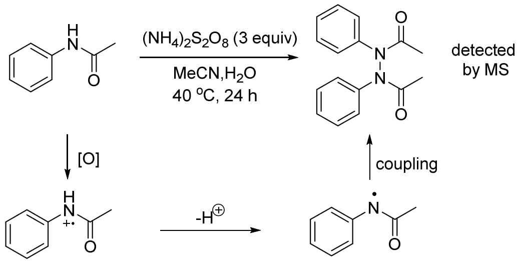

Scheme S8 APS mediated redox dimerization of N-phenylacetamide

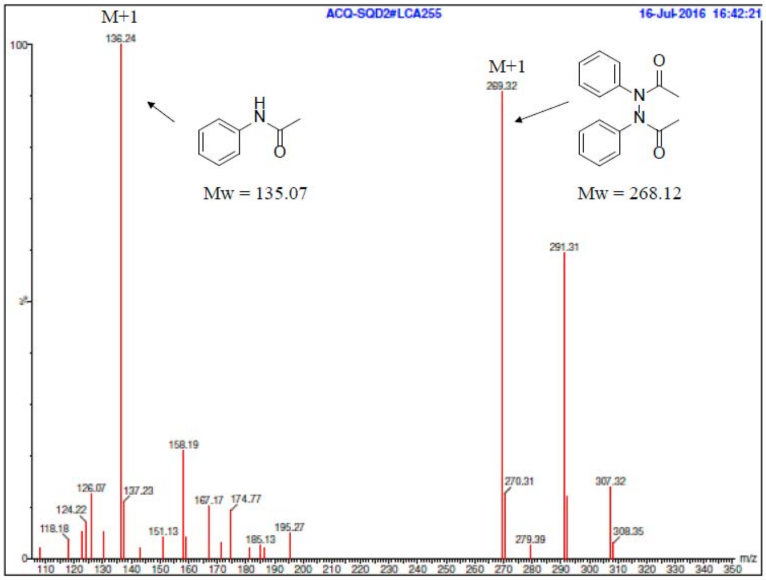

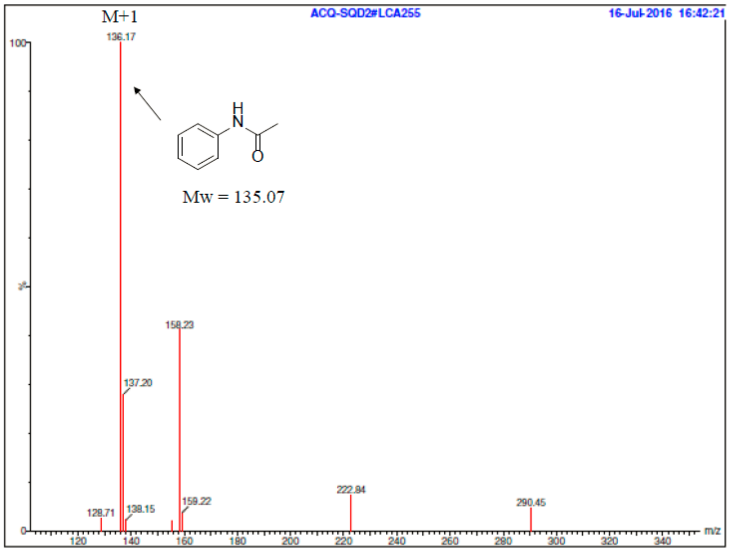

Fig. S6 MS data of N-phenylacetamide and crude product

The validation of neutral oxygen radical of NHS

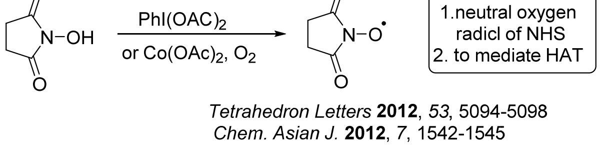

Scheme S9 The reported approach to generate neutral oxygen radical of NHS

Notes: NHS is able to produce neutral oxygen radical after treated with $PhI(OAc)_2$ or $Co(OAc)_2/O_2$ to mediate HAT process according to Yamamoto's work.[5] Therefore, the

control reactions (Scheme S10), as well as EPR experiment of NHS after treating with APS and $PhI(OAc)_2$ immediately, were performed to investigate if neutral oxygen radical was involved in our NHS/APS reaction system. The experiment result suggests that this radical might not be generated in our reaction system.

The control reactions to validate neutral oxygen radical of NHS

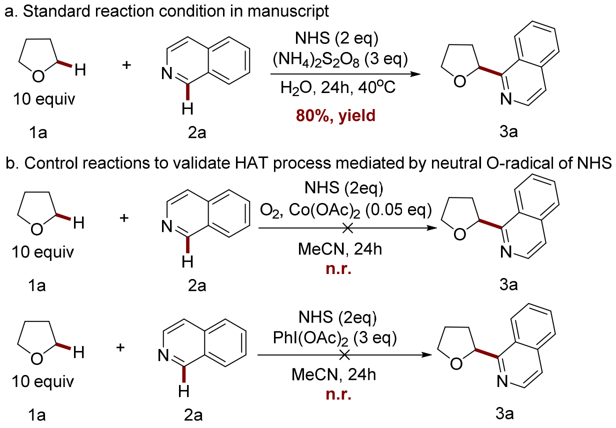

Scheme S10 Control reactions to generate neutral oxygen radical of NHS

Electroparamagnetic resonance (EPR) study

General procedure: the samples were prepared immediately before the acquisition, after tuning the ESR instrument parameters. The preparation is described as follows:

A solution of NHS (0.05 mmol, 1 equiv.) in CH_2Cl_2 (1.0 mL) was added to oxidant (0.05 mmol, 1 equiv.) in ESR tubes.[5]

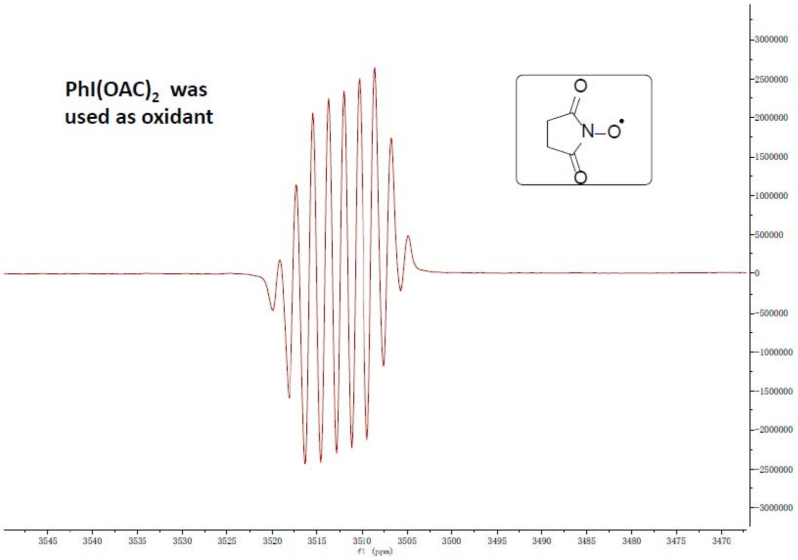

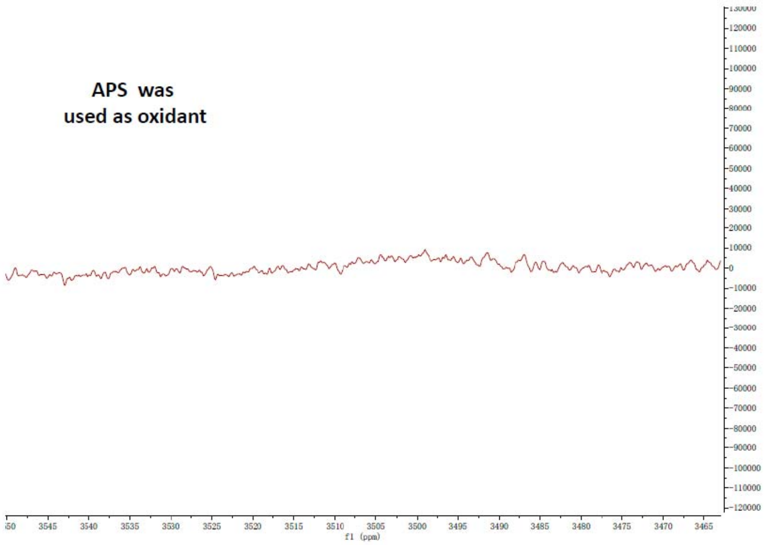

Fig. S7 The EPR spectra of the solution of NHS in water

The preliminary kinetic study of the reaction

Notes: The reaction kinetic was preliminary studied by collecting the time curve of the reaction with NHS or quinuclidin-3-ol as the additive. With NHS or quinuclidin-3-ol as additive at 40 ºC, the yield kept increasing during 24 h, and eventually 88% or 82% of yield were achieved. However, in the absence of these two additives, the highest yield (13%) was reached at 3 h, thereafter remained unchanged. Raising the temperature to 70 ºC (APS is easy to decompose at this temperature) resulted in moderate yield (less than 2 h, 63% yield).

Table S8 The preliminary kinetic study of NHS and quinuclidin-3-ol promoted CDC reaction [a]

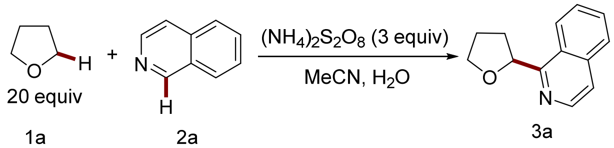

Entry	Time (h)	Yield (%) [b,c]	Yield (%) [b, d]	Yield (%) [b,e]	Yield (%) [b, f]
1	2	40%	36%	4%	63%
2	4	50%	44%	8%	63%
3	8	65%	61%	11%	64%
4	16	81%	76%	12%	60%
5	20	84%	79%	12%	53%
6	24	88%	82%	14%	50%

[a] Conditions employed **1a** (10.0 mmol), **2a** (0.5 mmol), $(NH_4)_2S_2O_8$ (1.5 mmol), 24 h, and a solvent mixture (1.5 mL, MeCN:H_2O = 1:1), unless otherwise noted; [b] Yields were determined by ^{1}H NMR using CH_2Br_2 as an internal standard; [c] performed with NHS (1.0 mmol) at 40 ºC; [d] performed with Quinuclidin-3-ol (1.0 mmol) at 40 ºC; [e] performed at 40 ºC without additive; [f] performed at 70ºC without additive.

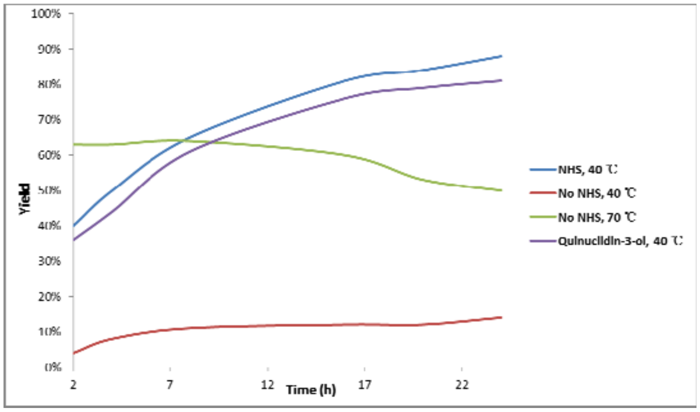

Fig. S8 The preliminary kinetic study of NHS and quinuclidin-3-ol promoted CDC reaction

Experiment Procedures and Product Characterization

Commercial reagents and solvents were used as received, unless otherwise stated. Organic solution was concentrated under reduced pressure on a Büchi rotary evaporator using an isopropyl alcohol-dry ice bath. Analytical thin layer chromatography (TLC) was performed on 0.25 mm silica gel plates (Qingdao Haiyang Chemical China), and the compounds were visualized with a UV light at 254 nm. Further visualization was achieved by staining with iodine. Flash chromatography was performed on silica gel 200–300 mesh (purchased from Qingdao Haiyang Chemical China) with commercial solvents (purchased from Adamas-beta®). The ^{1}H and ^{13}C NMR spectra were recorded on a Bruker AM 400 Spectrometer (400 and 100 MHz for ^{1}H and ^{13}C NMR, respectively) and are internally referenced to residual solvent signals (note: $CDCl_3$ referenced at 7.26 and 77.00 ppm in ^{1}H and ^{13}C NMR, respectively; d^6-DMSO referenced at 2.50 and 39.52 ppm in ^{1}H and ^{13}C NMR, respectively). Multiplicities were given as s (singlet), d (doublet), t (triplet), dd (double of doublet), and m (multiplets). Coupling constants were reported in Hertz (Hz). Data for ^{13}C NMR are reported in terms of chemical shift. High-resolution mass spectrometry (HRMS) was recorded on Waters LCT Premier XE spectrometer.

General procedure for Cα-heteroarylation of ether

To a 10 mL sealed tube equipped with a magnetic stir bar was charged a heteroarene (0.50 mmol, 1.0 equiv.), $(NH_4)_2S_2O_8$ (1.50 mmol, 3.0 equiv.), N-Hydroxy succinimide (1.00 mmol, 2.0 equiv.), 1.5 mL H_2O, and 1.5 mL solvent mixture (MeCN:H_2O = 1:1). After stirring at 40 °C for the indicated time, the reaction mixture was diluted with 2.5 mL of aqueous NaOH (1 M), and extracted with EtOAc (20 mL × 3). The combined organic extracts were washed with brine (20 mL × 2), dried over Na_2SO_4, and concentrated in vacuo. Purification of the crude product by flash chromatography on silica gel using the indicated solvent system afforded the desired product.

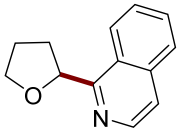

1-(Tetrahydrofuran-2-yl)isoquinoline (±3a): According to the general procedure, tetrahydrofuran (360 mg, 5.00 mmol, 10.0 equiv.), isoquinoline (65 mg, 0.50 mmol, 1.0 equiv.), $(NH_4)_2S_2O_8$ (342 mg, 1.50 mmol, 3.0 equiv.), N-Hydroxy succinimide (115 mg, 1.00 mmol, 2.0 equiv.) and 1.5 mL of H_2O were used. After 24 hours, the reaction mixture was subjected to the workup protocol outlined in the general procedure and purified by flash chromatography (10% ethyl acetate/petroleum ether) to provide the title compound as a colourless oil (80 mg, 80% yield). ^{1}H NMR (400 MHz, $CDCl_3$) δ ppm 8.46 (d, *J* = 5.7 Hz, 1H), 8.29 (d, *J* = 8.4 Hz, 1H), 7.76 (d, *J* = 8.0 Hz, 1H), 7.65 - 7.58 (m, 1H), 7.58 - 7.50 (m, 2H), 5.68 (t, *J* = 7.1 Hz, 1H), 4.15 (dd, *J* = 14.4, 7.4 Hz, 1H), 4.03 - 3.97 (m, 1H), 2.52 - 2.43 (m, 1H), 2.42 - 2.31 (m, 1H), 2.19 - 2.00 (m, 2H); ^{13}C NMR (100 MHz, $CDCl_3$) δ ppm 159.42, 141.38, 136.32, 129.63, 127.12, 126.92, 126.40, 125.08, 120.33, 78.92, 68.80, 30.59, 25.96; HRMS (ESI) Calcd. for $C_{13}H_{14}NO$ [$(M+H)^+$] 200.1075, found 200.1081.

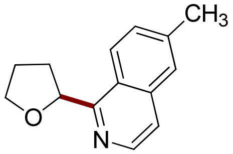

6-Methyl-1-(tetrahydrofuran-2-yl)isoquinoline (±3b): According to the general procedure, tetrahydrofuran (360 mg, 5.00 mmol, 10.0 equiv.), 6-methylisoquinoline (72 mg, 0.50 mmol, 1.0 equiv.), $(NH_4)_2S_2O_8$ (342 mg, 1.50 mmol, 3.0 equiv.), N-Hydroxy succinimide (115 mg, 1.00 mmol, 2.0 equiv.) and 1.5 mL of H_2O were used. After 24 hours, the reaction mixture was subjected to the workup protocol outlined in the general procedure and purified by flash chromatography (10% ethyl acetate/petroleum ether) to provide the title compound as a colourless oil (94 mg, 88% yield). ^{1}H NMR (400 MHz, $CDCl_3$) δ ppm 8.43 (d, *J* = 5.7 Hz, 1H), 8.19 (d, *J* = 8.7 Hz, 1H), 7.56 (s, 1H), 7.45 (d, *J* = 5.7 Hz, 1H), 7.40 (dd, *J* = 8.7, 1.6 Hz, 1H), 5.67 (t, *J* = 7.1 Hz, 1H), 4.17 (dd, *J* = 14.4, 7.5 Hz, 1H), 4.04 - 3.98 (m, 1H), 2.50 (s, 3H), 2.49 - 2.33 (m, 2H), 2.22 - 2.03 (m, 2H); ^{13}C NMR (100 MHz, $CDCl_3$) δ ppm 159.19, 141.58, 139.92, 136.75, 129.20, 126.09, 124.90, 119.91, 78.99, 68.84, 30.70, 26.05, 21.73; HRMS (ESI) Calcd. for $C_{14}H_{16}NO$ [$(M+H)^+$] 214.1232, found 214.1239.

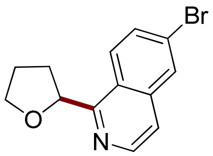

6-Bromo-1-(tetrahydrofuran-2-yl)isoquinoline (±3c): According to the general procedure, tetrahydrofuran (360 mg, 5.00 mmol, 10.0 equiv.), 6-bromoisoquinoline (104 mg, 0.50 mmol, 1.0 equiv.), $(NH_4)_2S_2O_8$ (342 mg, 1.50 mmol, 3.0 equiv.), N-Hydroxy succinimide (115 mg, 1.00 mmol, 2.0 equiv.) and 1.5 mL of H_2O were used. After 24 hours, the reaction mixture was subjected to the workup protocol outlined in the general procedure and purified by flash chromatography (10% ethyl acetate/petroleum ether) to provide the title compound as a white solid (118 mg, 85% yield). ^{1}H NMR (400 MHz, $CDCl_3$) δ ppm 8.45 (d, *J* = 5.7 Hz, 1H), 8.18 (d, *J* = 9.0 Hz, 1H), 7.91 (d, *J* = 1.7 Hz, 1H), 7.60 (dd, *J* = 9.0, 1.9 Hz, 1H), 7.41 (d, *J* = 5.7 Hz,

1H), 5.58 (t, J = 7.1 Hz, 1H), 4.10 (dd, J = 14.6, 7.3 Hz, 1H), 3.98 (dd, J = 14.2, 7.8 Hz, 1H), 2.61 - 2.42 (m, 1H), 2.37 - 2.29 (m, 1H), 2.22 - 1.96 (m, 2H); ^{13}C NMR (100 MHz, $CDCl_3$) δ ppm 159.72, 142.46, 137.56, 130.43, 129.20, 127.15, 124.94, 124.47, 119.33, 79.08, 68.88, 30.41, 25.98; HRMS (ESI) Calcd. for $C_{13}H_{13}BrNO$ [$(M+H)^+$] 278.0181, found 278.0167.

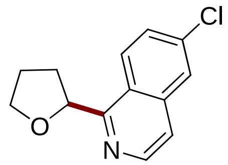

6-Chloro-1-(tetrahydrofuran-2-yl)isoquinoline (±3d): According to the general procedure, tetrahydrofuran (360 mg, 5.00 mmol, 10.0 equiv.), 6-chloroisoquinoline (82 mg, 0.50 mmol, 1.0 equiv.), $(NH_4)_2S_2O_8$ (342 mg, 1.50 mmol, 3.0 equiv.), N-Hydroxy succinimide (115 mg, 1.00 mmol, 2.0 equiv.) and 1.5 mL of H_2O were used. After 24 hours, the reaction mixture was subjected to the workup protocol outlined in the general procedure and purified by flash chromatography (10% ethyl acetate/petroleum ether) to provide the title compound as a white solid (105 mg, 90% yield). ^{1}H NMR (400 MHz, $CDCl_3$) δ ppm 8.46 (d, J = 5.7 Hz, 1H), 8.28 (d, J = 9.0 Hz, 1H), 7.76 (d, J = 1.7 Hz, 1H), 7.49 (dd, J = 9.0, 1.9 Hz, 1H), 7.44 (d, J = 5.7 Hz, 1H), 5.61 (t, J = 7.1 Hz, 1H), 4.12 (dd, J = 14.6, 7.3 Hz, 1H), 4.00 (dd, J = 14.2, 7.8 Hz, 1H), 2.59 - 2.43 (m, 1H), 2.39 - 2.31 (m, 1H), 2.19 - 2.04 (m, 2H); ^{13}C NMR (100 MHz, $CDCl_3$) δ ppm 159.62, 142.51, 137.29, 135.91, 127.92, 127.23, 125.86, 124.79, 119.50, 79.16, 68.89, 30.41, 26.00; HRMS (ESI) Calcd. for $C_{13}H_{13}ClNO$ [$(M+H)^+$] 234.0686, found 234.0690.

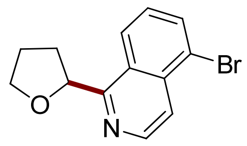

5-Bromo-1-(tetrahydrofuran-2-yl)isoquinoline (±3e): According to the general procedure, tetrahydrofuran (360 mg, 5.00 mmol, 10.0 equiv.), 5-bromoisoquinoline (104 mg, 0.50 mmol, 1.0 equiv.), $(NH_4)_2S_2O_8$ (342 mg, 1.50 mmol, 3.0 equiv.), N-Hydroxy succinimide (115 mg, 1.00 mmol, 2.0 equiv.) and 1.5 mL of H_2O were used. After 24 hours, the reaction mixture was subjected to the workup protocol outlined in the general procedure and purified by flash chromatography (10% ethyl acetate/petroleum ether) to provide the title compound as a white solid (125 mg, 90% yield). ^{1}H NMR (400 MHz, $CDCl_3$) δ ppm 8.56 (d, J = 5.9 Hz, 1H), 8.30 (d, J = 8.5 Hz, 1H), 7.92 (s, 1H), 7.91 - 7.88 (m, 1H), 7.40 (dd, J = 8.4, 7.6 Hz, 1H), 5.67 (t, J = 7.0 Hz, 1H), 4.12 (dd, J = 14.3, 7.5 Hz, 1H), 4.03 - 3.97 (m, 1H), 2.61 - 2.44 (m, 1H), 2.44 - 2.30 (m, 1H), 2.21 - 2.01 (m, 2H); ^{13}C NMR (101 MHz, $CDCl_3$) δ ppm 159.75, 142.66), 135.35, 133.38, 127.55, 127.16, 124.91, 122.03, 119.11, 78.77, 68.82, 30.40, 25.90; HRMS (ESI) Calcd. for $C_{13}H_{13}BrNO$ [$(M+H)^+$] 278.0181, found 278.0186.

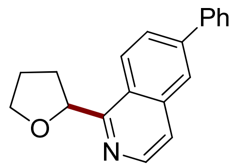

6-Phenyl-1-(tetrahydrofuran-2-yl)isoquinoline (±3f): According to the general procedure, tetrahydrofuran (360 mg, 5.00 mmol, 10.0 equiv.), 6-phenylisoquinoline (103 mg, 0.50 mmol, 1.0 equiv.), $(NH_4)_2S_2O_8$ (342 mg, 1.50 mmol, 3.0 equiv.), N-Hydroxy succinimide (115 mg, 1.00 mmol, 2.0 equiv.) and 1.5 mL of H_2O were used. After 24 hours, the reaction mixture was subjected to the workup protocol outlined in the general procedure and purified by flash chromatography (20% ethyl acetate/petroleum ether) to provide the title compound as a white solid (131 mg, 95% yield). ^{1}H NMR (400 MHz, $CDCl_3$) δ ppm 8.51 (d, J = 5.7 Hz, 1H), 8.39 (d, J = 8.8 Hz, 1H), 7.97 (d, J = 1.5 Hz, 1H), 7.83 (dd, J = 8.8, 1.8 Hz, 1H), 7.73 - 7.66 (m, 2H), 7.59 (d, J = 5.7 Hz, 1H), 7.48 (t, J = 7.5 Hz, 2H), 7.40 (t, J = 7.3 Hz, 1H), 5.72 (t, J = 7.1 Hz, 1H), 4.20 (dd, J = 14.5, 7.4 Hz, 1H), 4.07 - 4.02 (m, 1H), 2.59 - 2.50 (m, 1H), 2.47 - 2.34 (m, 1H), 2.24 - 2.05 (m, 2H); ^{13}C NMR (100 MHz, $CDCl_3$) δ ppm 159.38, 142.20, 141.85, 139.89, 136.76, 128.85, 127.97, 127.33, 126.65, 125.75, 125.50, 124.73, 120.57, 79.03, 68.84, 30.59, 26.02; HRMS (ESI) Calcd. for $C_{19}H_{18}NO$ [(M+H)$^+$] 276.1388, found 276.1383.

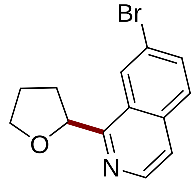

7-Bromo-1-(tetrahydrofuran-2-yl)isoquinoline (±3g): According to the general procedure, tetrahydrofuran (360 mg, 5.00 mmol, 10.0 equiv.), 7-bromoisoquinoline (104 mg, 0.50 mmol, 1.0 equiv.), $(NH_4)_2S_2O_8$ (342 mg, 1.50 mmol, 3.0 equiv.), N-Hydroxy succinimide (115 mg, 1.00 mmol, 2.0 equiv.) and 1.5 mL of H_2O were used. After 24 hours, the reaction mixture was subjected to the workup protocol outlined in the general procedure and purified by flash chromatography (10% ethyl acetate/petroleum ether) to provide the title compound as a white solid (118 mg, 85% yield). ^{1}H NMR (400 MHz, $CDCl_3$) δ ppm 8.49 (dd, J = 10.2, 3.2 Hz, 2H), 7.71 - 7.63 (m, 2H), 7.49 (d, J = 5.6 Hz, 1H), 5.57 (t, J = 7.1 Hz, 1H), 4.12 (dd, J = 14.5, 7.5 Hz, 1H), 4.03 - 3.98 (m, 1H), 2.57 - 2.48 (m, 1H), 2.43 - 2.30 (m, 1H), 2.21 - 2.02 (m, 2H); ^{13}C NMR (100 MHz, $CDCl_3$) δ ppm 158.61, 141.81, 134.83, 133.20, 128.80, 127.82, 127.52, 120.85, 120.09, 79.03, 68.89, 30.35, 26.00; HRMS (ESI) Calcd. for $C_{13}H_{13}BrNO$ [(M+H)$^+$] 278.0181, found 278.0187.

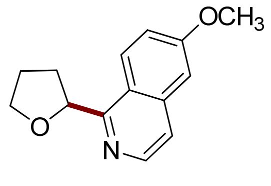

6-Methoxy-1-(tetrahydrofuran-2-yl)isoquinoline (±3h): According to the general procedure, tetrahydrofuran (360 mg, 5.00 mmol, 10.0 equiv.), 6-methoxyisoquinoline (80 mg, 0.50 mmol, 1.0 equiv.), $(NH_4)_2S_2O_8$ (342 mg, 1.50 mmol, 3.0 equiv.), N-Hydroxy succinimide (115 mg, 1.00 mmol, 2.0 equiv.) and 1.5 mL of H_2O were used. After 24 hours, the reaction mixture was subjected to the workup protocol outlined in the general procedure and purified by flash chromatography (10% ethyl acetate/petroleum ether) to provide the title compound as a colourless oil (83 mg, 72% yield). ^1H NMR (400 MHz, $CDCl_3$) δ ppm 8.40 (d, *J* = 5.7 Hz, 1H), 8.23 (d, *J* = 9.3 Hz, 1H), 7.45 (d, *J* = 5.7 Hz, 1H), 7.20 (dd, *J* = 9.3, 2.4 Hz, 1H), 7.04 (d, *J* = 2.3 Hz, 1H), 5.62 (t, *J* = 7.2 Hz, 1H), 4.16 (dd, *J* = 14.5, 7.4 Hz, 1H), 4.00 (dd, *J* = 14.1, 7.8 Hz, 1H), 3.91 (s, 3H), 2.53 - 2.42 (m, 1H), 2.40 - 2.32 (m, 1H), 2.20 - 2.05 (m, 2H); ^{13}C NMR (100 MHz, $CDCl_3$) δ ppm 160.23, 158.94, 142.13, 138.59, 127.03, 122.21, 119.80, 104.70, 79.16, 68.87, 55.32, 30.75, 26.07; HRMS (ESI) Calcd. for $C_{14}H_{16}NO_2$ [$(M+H)^+$] 230.1181, found 230.1202.

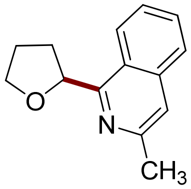

3-methyl-1-(tetrahydrofuran-2-yl)isoquinoline (±3i): According to the general procedure, tetrahydrofuran (360 mg, 5.00 mmol, 10.0 equiv.), 3-methylisoquinoline (72 mg, 0.50 mmol, 1.0 equiv.), $(NH_4)_2S_2O_8$ (342 mg, 1.50 mmol, 3.0 equiv.), N-Hydroxy succinimide (115 mg, 1.00 mmol, 2.0 equiv.) and 1.5 mL of H_2O were used. After 24 hours, the reaction mixture was subjected to the workup protocol outlined in the general procedure and purified by flash chromatography (10% ethyl acetate/petroleum ether) to provide the title compound as a colourless oil (83 mg, 78% yield). ^1H NMR (400 MHz, $CDCl_3$) δ ppm 8.30 (d, *J* = 8.3 Hz, 1H), 7.71 (d, *J* = 8.1 Hz, 1H), 7.59 (t, *J* = 7.2 Hz, 1H), 7.49 (t, *J* = 7.4 Hz, 1H), 7.38 (s, 1H), 5.65 (t, *J* = 7.1 Hz, 1H), 4.20 (dd, *J* = 14.3, 7.3 Hz, 1H), 4.02 (dd, *J* = 14.2, 7.5 Hz, 1H), 2.67 (s, 3H), 2.58 - 2.50 (m, 1H), 2.40 - 2.32 (m, 1H), 2.21 - 2.06 (m, 2H); ^{13}C NMR (100 MHz, $CDCl_3$) δ ppm 158.72, 150.03, 137.28, 129.51, 126.59, 125.88, 125.17, 124.59, 118.32, 79.67, 68.86, 30.59, 25.98, 24.28; HRMS (ESI) Calcd. for $C_{14}H_{16}NO$ [$(M+H)^+$] 214.1232, found 214.1237.

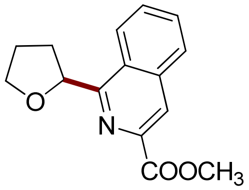

Methyl 1-(tetrahydrofuran-2-yl)isoquinoline-3-carboxylate (±3j): According to the general procedure, tetrahydrofuran (360 mg, 5.00 mmol, 10.0 equiv.), methyl isoquinoline-3-carboxylate (94 mg, 0.50 mmol, 1.0 equiv.), $(NH_4)_2S_2O_8$ (342 mg, 1.50 mmol, 3.0 equiv.), N-Hydroxy succinimide (115 mg, 1.00 mmol, 2.0 equiv.) and 1.5

mL of H_2O were used. After 24 hours, the reaction mixture was subjected to the workup protocol outlined in the general procedure and purified by flash chromatography (20% ethyl acetate/petroleum ether) to provide the title compound as a white solid (104 mg, 81% yield). ^{1}H NMR (400 MHz, $CDCl_3$) δ ppm 8.53 - 8.44 (m, 2H), 7.94 (dd, J = 6.6, 2.7 Hz, 1H), 7.77 - 7.64 (m, 2H), 5.65 (t, J = 7.2 Hz, 1H), 4.16 (dd, J = 14.7, 7.5 Hz, 1H), 4.07 - 3.94 (m, 4H), 2.73 - 2.64 (m, 1H), 2.50 - 2.33 (m, 1H), 2.28 - 2.05 (m, 2H); ^{13}C NMR (100 MHz, $CDCl_3$) δ ppm 166.50, 160.01, 140.02, 136.38, 130.51, 129.31, 128.77, 128.17), 126.00, 124.14, 80.40, 68.98, 52.69, 30.26, 26.10; HRMS (ESI) Calcd. for $C_{15}H_{16}NO_3$ [$(M+H)^+$] 258.1130, found 258.1125.

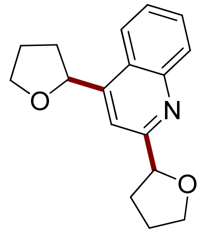

2,4-bis(tetrahydrofuran-2-yl)quinoline (±3k): According to the general procedure, tetrahydrofuran (360 mg, 5.00 mmol, 10.0 equiv.), quinoline (65 mg, 0.50 mmol, 1.0 equiv.), $(NH_4)_2S_2O_8$ (342 mg, 1.50 mmol, 3.0 equiv.), N-Hydroxy succinimide (115 mg, 1.00 mmol, 2.0 equiv.) and 1.5 mL of H_2O were used. After 72 hours, the reaction mixture was subjected to the workup protocol outlined in the general procedure and purified by flash chromatography (10% ethyl acetate/petroleum ether) to provide the title mixture as a colourless oil (102mg, 75% yield). ^{1}H NMR (400 MHz, $CDCl_3$) δ ppm 8.07 (dd, J = 8.1, 6.0 Hz, 1H), 7.87 (t, J = 8.4 Hz, 1H), 7.76 - 7.62 (m, 2H), 7.49 (dd, J = 8.2, 7.0 Hz, 1H), 5.57 (dd, J = 17.0, 7.2 Hz, 1H), 5.22 - 5.07 (m, 1H), 4.29 - 4.10 (m, 2H), 4.08 - 3.95 (m, 2H), 2.66 - 2.53 (m, 1H), 2.51 - 2.43 (m, 1H), 2.15 - 1.93 (m, 5H), 1.92 - 1.76 (m, 1H); ^{13}C NMR (101 MHz, $CDCl_3$) δ 163.56, 163.10, 150.01, 149.74, 147.54, 147.38, 129.81, 129.68, 128.89, 128.95, 125.80, 124.74, 124,70, 123.12, 123,02, 113.72, 113.00, 82.21, 81.95, 77.11, 76.90, 69.26, 69.09, 68.92, 68.83, 33.82, 33.72, 33.49, 32.99, 25.90, 25.87. HRMS (ESI) Calcd. for $C_{17}H_{20}NO_2$ [$(M+H)^+$] 270.1489, found 270.1491.

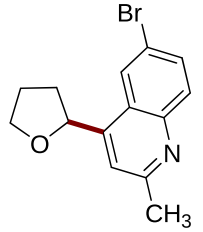

6-Bromo-2-methyl-4-(tetrahydrofuran-2-yl)quinoline (±3l): According to the general procedure, tetrahydrofuran (360 mg, 5.00 mmol, 10.0 equiv.), 6-bromo-2-methylquinoline (111 mg, 0.50 mmol, 1.0 equiv.), $(NH_4)_2S_2O_8$ (342 mg, 1.50 mmol, 3.0 equiv.), N-Hydroxy succinimide (115 mg, 1.00 mmol, 2.0 equiv.) and 1.5 mL of H_2O were used. After 24 hours, the reaction mixture was subjected to the workup protocol outlined in the general procedure and purified by flash

chromatography (10% ethyl acetate/petroleum ether) to provide the title compound as a white solid (127 mg, 87% yield). ^{1}H NMR (400 MHz, $CDCl_3$) δ ppm 7.93 (d, *J* = 2.1 Hz, 1H), 7.85 (d, *J* = 9.0 Hz, 1H), 7.67 (dd, *J* = 9.0, 2.1 Hz, 1H), 7.41 (s, 1H), 5.41 (t, *J* = 7.2 Hz, 1H), 4.17 (dd, *J* = 13.5, 7.7 Hz, 1H), 3.99 (dd, *J* = 15.3, 7.1 Hz, 1H), 2.67 (s, 3H), 2.61 - 2.50 (m, 1H), 2.11 - 1.90 (m, 2H), 1.81 - 1.72 (m, 1H); ^{13}C NMR (100 MHz, $CDCl_3$) δ ppm 159.47, 148.31, 146.36, 132.17, 130.98, 125.34, 125.02, 119.28, 117.92, 68.85, 33.72, 25.87, 25.40; HRMS (ESI) Calcd. for $C_{14}H_{15}BrNO$ [$(M+H)^+$] 292.0337, found 292.0401.

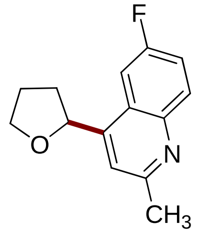

6-Fluoro-2-methyl-4-(tetrahydrofuran-2-yl)quinoline (±3m): According to the general procedure, tetrahydrofuran (360 mg, 5.00 mmol, 10.0 equiv.), 6-fluoro-2-methylquinoline (81 mg, 0.50 mmol, 1.0 equiv.), $(NH_4)_2S_2O_8$ (342 mg, 1.50 mmol, 3.0 equiv.), N-Hydroxy succinimide (115 mg, 1.00 mmol, 2.0 equiv.) and 1.5 mL of H_2O were used. After 24 hours, the reaction mixture was subjected to the workup protocol outlined in the general procedure and purified by flash chromatography (10% ethyl acetate/petroleum ether) to provide the title compound as a white solid (102 mg, 88% yield). ^{1}H NMR (400 MHz, $CDCl_3$) δ ppm 7.99 (dd, *J* = 9.1, 5.6 Hz, 1H), 7.43 - 7.34 (m, 3H), 5.38 (t, *J* = 7.2 Hz, 1H), 4.23 - 4.11 (m, 1H), 3.98 (dd, *J* = 15.4, 7.1 Hz, 1H), 2.68 (s, 3H), 2.57 - 2.49 (m, 1H), 2.09 - 1.89 (m, 2H), 1.81 - 1.72 (m, 1H); ^{13}C NMR (100 MHz, $CDCl_3$) δ ppm 160.82, 158.37, 159.23, 158.20, 148,62, 149.56, 144.85, 131.60, 131.51, 124.34, 124.25, 118.83, 118.58, 117.84, 106.85, 106.63, 68.84, 33.46, 25.81, 25.21; HRMS (ESI) Calcd. for $C_{14}H_{15}FNO$ [$(M+H)^+$] 232.1138, found 232.1143.

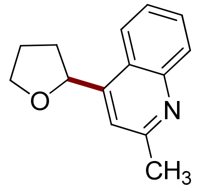

2-Methyl-4-(tetrahydrofuran-2-yl)quinoline (±3n): According to the general procedure, tetrahydrofuran (360 mg, 5.00 mmol, 10.0 equiv.), 2-methylquinoline (72 mg, 0.50 mmol, 1.0 equiv.), $(NH_4)_2S_2O_8$ (342 mg, 1.50 mmol, 3.0 equiv.), N-Hydroxy succinimide (115 mg, 1.00 mmol, 2.0 equiv.) and 1.5 mL of H_2O were used. After 24 hours, the reaction mixture was subjected to the workup protocol outlined in the general procedure and purified by flash chromatography (10% ethyl acetate/petroleum ether) to provide the title compound as a colourless oil (85 mg, 80% yield). ^{1}H NMR (400 MHz, $CDCl_3$) δ ppm 8.03 (dd, *J* = 8.4, 0.5 Hz, 1H), 7.81 (dd, *J* = 8.4, 0.8 Hz, 1H), 7.64 (ddd, *J* = 8.4, 6.9, 1.3 Hz, 1H), 7.47 - 7.43 (m, 1H), 7.42 (d, *J* = 0.7 Hz, 1H), 5.54 (t, *J* = 7.1 Hz, 1H), 4.25 - 4.14 (m, 1H), 4.07 - 3.94 (m, 1H), 2.72

(s, 3H), 2.63 - 2.51 (m, 1H), 2.09 - 1.91 (m, 2H), 1.87 - 1.74 (m, 1H); ^{13}C NMR (100 MHz, $CDCl_3$) δ ppm 159.08, 149.42, 147.78, 129.23, 128.99, 125.51, 123.84, 123.00, 117.21, 76.75, 68.98, 63.48, 33.88, 25.97; HRMS (ESI) Calcd. for $C_{14}H_{16}NO$ [$(M+H)^+$] 214.1232, found 214.1245.

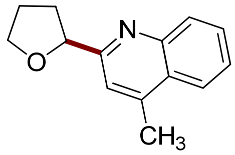

4-Methyl-2-(tetrahydrofuran-2-yl)quinoline (±3o): According to the general procedure, tetrahydrofuran (360 mg, 5.00 mmol, 10.0 equiv.), 4-methylquinoline (72 mg, 0.50 mmol, 1.0 equiv.), $(NH_4)_2S_2O_8$ (342 mg, 1.50 mmol, 3.0 equiv.), N-Hydroxy succinimide (115 mg, 1.00 mmol, 2.0 equiv.) and 1.5 mL of H_2O were used. After 24 hours, the reaction mixture was subjected to the workup protocol outlined in the general procedure and purified by flash chromatography (10% ethyl acetate/petroleum ether) to provide the title compound as a colourless oil (83 mg, 78% yield). ^{1}H NMR (400 MHz, $CDCl_3$) δ ppm 8.05 (d, J = 8.4 Hz, 1H), 7.96 (d, J = 8.3 Hz, 1H), 7.72 -7.64 (m, 1H), 7.54 - 7.48 (m, 1H), 7.44 (s, 1H), 5.13 (t, J = 6.9 Hz, 1H), 4.23 - 4.14 (m, 1H), 4.06 - 4.01 (m, 1H), 2.70 (s, 3H), 2.56 - 2.44 (m, 1H), 2.13 - 1.93 (m, 3H); ^{13}C NMR (100 MHz, $CDCl_3$) δ ppm 163.04, 147.27, 144.90, 129.48, 129.08, 127.40, 125.76, 123.64, 118.54, 82.03, 69.21, 33.28, 25.93, 18.85; HRMS (ESI) Calcd. for $C_{14}H_{16}NO$ [$(M+H)^+$] 214.1232, found 214.1240.

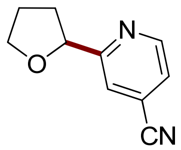

2-(Tetrahydrofuran-2-yl) isonicotinonitrile (±3p): According to the general procedure, tetrahydrofuran (360 mg, 5.00 mmol, 10.0 equiv.), isonicotinonitrile (52 mg, 0.50 mmol, 1.0 equiv.), $(NH_4)_2S_2O_8$ (342 mg, 1.50 mmol, 3.0 equiv.), N-Hydroxy succinimide (115 mg, 1.00 mmol, 2.0 equiv.) and 1.5 mL of H_2O were used. After 24 hours, the reaction mixture was subjected to the workup protocol outlined in the general procedure and purified by flash chromatography (10% ethyl acetate/petroleum ether) to provide the title compound as a colourless oil (49 mg, 56% yield). ^{1}H NMR (400 MHz, $CDCl_3$) δ ppm 8.71 (dd, J = 5.0, 0.7 Hz, 1H), 7.74 - 7.69 (m, 1H), 7.38 (dd, J = 4.8, 1.3 Hz, 1H), 5.08 - 5.02 (m, 1H), 4.15 - 4.07 (m, 1H), 4.04 - 3.96 (m, 1H), 2.51 - 2.41 (m, 1H), 2.05 - 1.89 (m, 3H); ^{13}C NMR (100 MHz, $CDCl_3$) δ ppm 165.23, 149.91, 123.37, 121.59, 120.88, 116.72, 80.63 , 69.28, 33.03, 25.65; HRMS (ESI) Calcd. for $C_{10}H_{11}N_2O$ [$(M+H)^+$] 175.0871, found 175.0880.

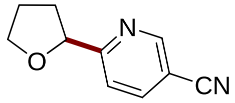

6-(Tetrahydrofuran-2-yl)nicotinonitrile (±3q): According to the general procedure,

tetrahydrofuran (360 mg, 5.00 mmol, 10.0 equiv.), nicotinonitrile (52 mg, 0.50 mmol, 1.0 equiv.), $(NH_4)_2S_2O_8$ (342 mg, 1.50 mmol, 3.0 equiv.), N-Hydroxy succinimide (115 mg, 1.00 mmol, 2.0 equiv.) and 1.5 mL of H_2O were used. After 24 hours, the reaction mixture was subjected to the workup protocol outlined in the general procedure and purified by flash chromatography (10% ethyl acetate/petroleum ether) to provide the title compound as a white solid (54 mg, 62% yield). ^{1}H NMR (400 MHz, $CDCl_3$) δ ppm 8.79 (d, *J* = 1.4 Hz, 1H), 7.93 (dd, *J* = 8.2, 2.1 Hz, 1H), 7.59 (d, *J* = 8.2 Hz, 1H), 5.04 (dd, *J* = 7.4, 5.9 Hz, 1H), 4.07 (ddd, *J* = 6.3, 5.6, 2.4 Hz, 1H), 4.02 - 3.94 (m, 1H), 2.50 - 2.39 (m, 1H), 2.06 - 1.87 (m, 3H); ^{13}C NMR (100 MHz, $CDCl_3$) δ ppm 167.76, 151.73, 139.73, 119.67, 116.76, 107.98, 80.79, 69.25, 32.97, 25.62; HRMS (ESI) Calcd. for $C_{10}H_{11}N_2O$ [$(M+H)^+$] 175.0871, found 175.0880.

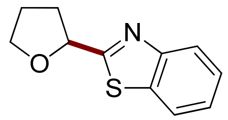

2-(Tetrahydrofuran-2-yl)benzo[d]thiazole (±3r): According to the general procedure, tetrahydrofuran (360 mg, 5.00 mmol, 10.0 equiv.), benzo[d]thiazole (68 mg, 0.50 mmol, 1.0 equiv.), $(NH_4)_2S_2O_8$ (342 mg, 1.50 mmol, 3.0 equiv.), N-Hydroxy succinimide (115 mg, 1.00 mmol, 2.0 equiv.) and 1.5 mL of H_2O were used. After 24 hours, the reaction mixture was subjected to the workup protocol outlined in the general procedure and purified by flash chromatography (5% ethyl acetate/petroleum ether) to provide the title compound as a colourless oil (57 mg, 56% yield). ^{1}H NMR (400 MHz, $CDCl_3$) δ ppm 7.97 (d, *J* = 8.2 Hz, 1H), 7.88 (dd, *J* = 8.0, 0.6 Hz, 1H), 7.46 (ddd, *J* = 8.3, 7.3, 1.3 Hz, 1H), 7.38 - 7.34 (m, 1H), 5.35 (dd, *J* = 7.8, 5.4 Hz, 1H), 4.19 - 4.13 (m, 1H), 4.03 - 3.98 (m, 1H), 2.57 - 2.48 (m, 1H), 2.32 - 2.22 (m, 1H), 2.09 - 1.98 (m, 2H); ^{13}C NMR (100 MHz, $CDCl_3$) δ ppm 176.43, 153.68, 134.74, 125.95, 124.79, 122.79, 121.80, 78.77, 69.49, 33.41, 25.72; HRMS (ESI) Calcd. for $C_{11}H_{12}NOS$ [$(M+H)^+$] 206.0640, found 206.0645.

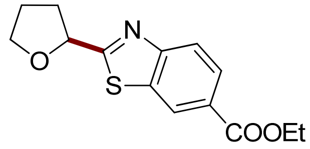

Ethyl 2-(tetrahydrofuran-2-yl)benzo[d]thiazole-6-carboxylate (±3s): According to the general procedure, tetrahydrofuran (360 mg, 5.00 mmol, 10.0 equiv.), ethyl benzo[d]thiazole-6-carboxylate (104 mg, 0.50 mmol, 1.0 equiv.), $(NH_4)_2S_2O_8$ (342 mg, 1.50 mmol, 3.0 equiv.), N-Hydroxy succinimide (115 mg, 1.00 mmol, 2.0 equiv.) and 1.5 mL of H_2O were used. After 24 hours, the reaction mixture was subjected to the workup protocol outlined in the general procedure and purified by flash chromatography (10% ethyl acetate/petroleum ether) to provide the title compound as a white solid (67 mg, 48% yield). ^{1}H NMR (400 MHz, $CDCl_3$) δ ppm 8.60 (d, *J* = 1.3 Hz, 1H), 8.14 (dd, *J* = 8.6, 1.7 Hz, 1H), 7.98 (d, *J* = 8.6 Hz, 1H), 5.35 (dd, *J* = 7.9, 5.4 Hz, 1H), 4.41 (q, *J* = 7.1 Hz, 2H), 4.22 - 4.11 (m, 1H), 4.01 (dd, *J* = 15.3, 7.1 Hz, 1H), 2.58 - 2.49 (m, 1H), 2.31 - 2.23 (m, 1H), 2.09 - 1.96 (m, 2H), 1.42 (t, *J* = 7.1 Hz, 3H);

^{13}C NMR (100 MHz, $CDCl_3$) δ ppm 180.25, 166.17, 156.57, 134.60, 127.13, 126.91, 123.93, 122.40, 78.75), 69.54, 61.21, 33.33, 25.68, 14.32; HRMS (ESI) Calcd. for $C_{14}H_{16}NO_3S$ [$(M+H)^+$] 278.0851, found 278.0847.

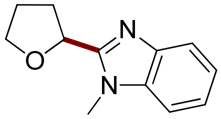

1-Methyl-2-(tetrahydrofuran-2-yl)-1H-benzo[d]imidazole (±3u): According to the general procedure, tetrahydrofuran (360 mg, 5.00 mmol, 10.0 equiv.), 1-methyl-1H-benzo[d]imidazole (66 mg, 0.50 mmol, 1.0 equiv.), $(NH_4)_2S_2O_8$ (342 mg, 1.50 mmol, 3.0 equiv.), N-Hydroxy succinimide (115 mg, 1.00 mmol, 2.0 equiv.) and 1.5 mL of H_2O were used. After 24 hours, the reaction mixture was subjected to the workup protocol outlined in the general procedure and purified by flash chromatography (50% ethyl acetate/petroleum ether) to provide the title compound as a white solid (46 mg, 45% yield). ^1H NMR (400 MHz, $CDCl_3$) δ ppm 7.76 (dd, J = 6.7, 1.8 Hz, 1H), 7.36 - 7.18 (m, 3H), 5.19 (t, J = 6.9 Hz, 1H), 3.93 (t, J = 6.8 Hz, 2H), 3.84 (s, 3H), 2.86 - 2.71 (m, 1H), 2.42 - 2.28 (m, 1H), 2.24 - 2.14 (m, 1H), 2.12 - 1.97 (m, 1H); ^{13}C NMR (100 MHz, $CDCl_3$) δ ppm 153.49, 141.79, 136.35, 122.62, 121.90, 119.69, 109.09, 73.48, 68.63, 30.13, 29.26, 25.96; HRMS (ESI) Calcd. for $C_{12}H_{15}N_2O$ [$(M+H)^+$] 203.1184, found 203.1189.

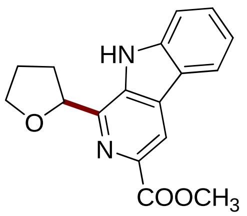

Methyl 1-(tetrahydrofuran-2-yl)-9H-pyrido[3,4-b]indole-3-carboxylate (±3w): According to the general procedure, tetrahydrofuran (360 mg, 5.00 mmol, 10.0 equiv.), methyl 9H-pyrido[3,4-b]indole-3-carboxylate [6] (113 mg, 0.50 mmol, 1.0 equiv.), $(NH_4)_2S_2O_8$ (342 mg, 1.50 mmol, 3.0 equiv.), N-Hydroxy succinimide (115 mg, 1.00 mmol, 2.0 equiv.) and 1.5 mL of H_2O were used. After 24 hours, the reaction mixture was subjected to the workup protocol outlined in the general procedure and purified by flash chromatography (10% methanol/ dichloromethane) to provide the title compound as a white solid (135 mg, 91% yield). ^1H NMR (400 MHz, $CDCl_3$) δ ppm 9.70 (s, 1H), 8.78 (s, 1H), 8.15 (d, J = 7.9 Hz, 1H), 7.63 - 7.50 (m, 2H), 7.33 - 7.29 (m, 1H), 5.51 (t, J = 7.1 Hz, 1H), 4.16 (dd, J = 14.0, 7.8 Hz, 1H), 4.10 - 3.99 (m, 4H), 2.66 - 2.58 (m, 1H), 2.43 - 2.34 (m, 1H), 2.12 - 1.92 (m, 2H); ^{13}C NMR (100 MHz, $CDCl_3$) δ ppm 166.70, 145.37, 140.31, 136.61, 134.97, 129.39, 128.71, 121.64, 121.31, 120.51, 116.79, 111.90, 82.78, 69.02, 52.55, 32.20, 25.43; HRMS (ESI) Calcd. for $C_{17}H_{17}N_2O_3$ [$(M+H)^+$] 297.1239, found 297.1243.

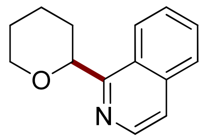

1-(Tetrahydro-2H-pyran-2-yl)isoquinoline (±4a): According to the general procedure, tetrahydro-2H-pyran (430 mg, 5.00 mmol, 10.0 equiv.), isoquinoline (65 mg, 0.50 mmol, 1.0 equiv.), $(NH_4)_2S_2O_8$ (342 mg, 1.50 mmol, 3.0 equiv.), N-Hydroxy succinimide (115 mg, 1.00 mmol, 2.0 equiv.) and 1.5 mL of $MeCN/H_2O$ (1: 1) were used. After 24 hours, the reaction mixture was subjected to the workup protocol outlined in the general procedure and purified by flash chromatography (10% ethyl acetate/petroleum ether) to provide the title compound as a colourless oil (91 mg, 85% yield). ^1H NMR (400 MHz, $CDCl_3$) δ ppm 8.41 (dd, J = 5.6, 0.9 Hz, 1H), 8.24 (d, J = 8.4 Hz, 1H), 7.68 (d, J = 8.2 Hz, 1H), 7.52 (ddd, J = 8.2, 7.0, 1.3 Hz, 1H), 7.49 - 7.40 (m, 2H), 5.07 (d, J = 11.1 Hz, 1H), 4.15 (d, J = 12.7 Hz, 1H), 3.67 (t, J = 11.6 Hz, 1H), 2.09 - 1.84 (m, 3H), 1.81 - 1.63 (m, 2H), 1.54 (d, J = 12.1 Hz, 1H); ^{13}C NMR (100 MHz, $CDCl_3$) δ ppm 159.47, 141.51, 136.42, 129.56, 127.15, 126.79, 125.83, 125.03, 120.33, 79.08, 69.20, 30.88, 25.70, 23.79; HRMS (ESI) Calcd. for $C_{14}H_{16}NO$ $[(M+H)^+]$ 214.1232, found 214.1227.

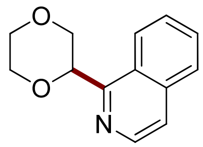

1-(1,4-Dioxan-2-yl)isoquinoline (±4b): According to the general procedure, 1,4-dioxane (440 mg, 5.00 mmol, 10.0 equiv.), isoquinoline (65mg, 0.50 mmol, 1.0 equiv.), $(NH_4)_2S_2O_8$ (342 mg, 1.50 mmol, 3.0 equiv.), N-Hydroxy succinimide (115 mg, 1.00 mmol, 2.0 equiv.) and 1.5 mL of H_2O were used. After 12 hours, the reaction mixture was subjected to the workup protocol outlined in the general procedure and purified by flash chromatography (10% ethyl acetate/petroleum ether) to provide the title compound as a white solid (97 mg, 90% yield). ^1H NMR (400 MHz, $CDCl_3$) δ ppm 8.48 (d, J = 5.7 Hz, 1H), 8.26 (d, J = 8.6 Hz, 1H), 7.77 (d, J = 7.7 Hz, 1H), 7.63 (ddd, J = 8.2, 6.9, 1.3 Hz, 1H), 7.60 - 7.53 (m, 2H), 5.42 (dd, J = 9.6, 3.0 Hz, 1H), 4.14 (dd, J = 11.9, 3.0 Hz, 1H), 4.11 - 3.98 (m, 3H), 3.90 - 3.80 (m, 2H); ^{13}C NMR (100 MHz, $CDCl_3$) δ ppm 155.79, 141.61, 136.26, 129.84, 127.27, 126.30, 124.51, 120.88, 75.65, 70.08, 67.38, 66.32; HRMS (ESI) Calcd. for $C_{13}H_{14}NO_2$ $[(M+H)^+]$ 216.1025, found 216.0968.

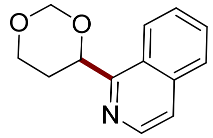

1-(1,3-Dioxan-4-yl)isoquinoline(±4c major isomer): According to the general procedure, 1,3-dioxane (440 mg, 5.00 mmol, 10.0 equiv.), isoquinoline (65 mg, 0.50 mmol, 1.0 equiv.), $(NH_4)_2S_2O_8$ (342 mg, 1.50 mmol, 3.0 equiv.), N-Hydroxy

succinimide (115 mg, 1.00 mmol, 2.0 equiv.) and 1.5 mL of MeCN/H_2O (1: 1) were used. After 24 hours, the reaction mixture was subjected to the workup protocol outlined in the general procedure and purified by flash chromatography (20% ethyl acetate/petroleum ether) to provide the title compound as a colourless oil (49 mg, 46% yield). ^{1}H NMR (400 MHz, $CDCl_3$) δ ppm 8.50 (d, J = 5.7 Hz, 1H), 8.47 (d, J = 8.6 Hz, 1H), 7.84 (d, J = 8.1 Hz, 1H), 7.68 (ddd, J = 8.2, 6.9, 1.2 Hz, 1H), 7.65 – 7.57 (m, 2H), 5.47 (dd, J = 11.1, 2.6 Hz, 1H), 5.30 (d, J = 6.3 Hz, 1H), 5.06 (d, J = 6.3 Hz, 1H), 4.34 (dd, J = 11.4, 4.7 Hz, 1H), 4.06 - 3.99 (m, 1H), 2.78 - 2.60 (m, 1H), 1.91 - 1.87 (m, 1H); ^{13}C NMR (100 MHz, $CDCl_3$) δ ppm 157.57, 141.52, 136.77, 129.94, 127.39, 127.21, 126.22, 125.36, 121.11, 94.21, 78.71, 66.94, 30.48; HRMS (ESI) m/z calculated for $C_{13}H_{14}NO_2$ [$(M+H)^+$] 216.1025, found 216.1032.

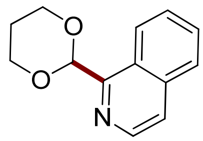

1-(1,3-dioxan-2-yl) isoquinoline (±4c minor isomer): According to the general procedure, 1,3-dioxane (440 mg, 5.00 mmol, 10.0 equiv.), isoquinoline (65 mg, 0.50 mmol, 1.0 equiv.), $(NH_4)_2S_2O_8$ (342 mg, 1.50 mmol, 3.0 equiv.), N-Hydroxy succinimide (115 mg, 1.00 mmol, 2.0 equiv.) and 1.5 mL of MeCN/H_2O (1: 1) were used. After 24 hours, the reaction mixture was subjected to the workup protocol outlined in the general procedure and purified by flash chromatography (20% ethyl acetate/petroleum ether) to provide the title compound as a colourless oil (30 mg, 28% yield). ^{1}H NMR (400 MHz, $CDCl_3$) δ ppm 8.69 (d, J = 8.6 Hz, 1H), 8.49 (d, J = 5.7 Hz, 1H), 7.81 (d, J = 7.9 Hz, 1H), 7.71 - 7.56 (m, 3H), 6.11 (s, 1H), 4.39 (dd, J = 11.3, 4.5 Hz, 2H), 4.17 - 4.10 (m, 2H), 2.53 - 2.41 (m, 1H), 1.60 - 1.53 (m, 1H); ^{13}C NMR (100 MHz, $CDCl_3$) δ ppm 154.88, 141.20, 137.04, 130.03, 127.19, 126.95, 126.38, 125.88, 122.00, 104.30, 67.86, 25.96; HRMS (ESI) Calcd. for $C_{13}H_{14}NO_2$ [$(M+H)^+$] 216.1025, found 216.1032.

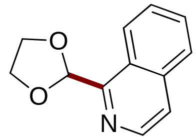

1-(1,3-Dioxolan-2-yl)isoquinoline (±4d major isomer): According to the general procedure, 1,3-dioxolane (370 mg, 5.00 mmol, 10.0 equiv.), isoquinoline (65 mg, 0.50 mmol, 1.0 equiv.), $(NH_4)_2S_2O_8$ (342 mg, 1.50 mmol, 3.0 equiv.), N-Hydroxy succinimide (115 mg, 1.00 mmol, 2.0 equiv.) and 1.5 mL of H_2O were used. After 24 hours, the reaction mixture was subjected to the workup protocol outlined in the general procedure and purified by flash chromatography (10% ethyl acetate/petroleum ether) to provide the title compound as a colourless oil (83 mg, 82% yield). ^{1}H NMR (400 MHz, $CDCl_3$) δ ppm 8.53 (d, J = 5.6 Hz, 1H), 8.39 (d, J = 8.5 Hz, 1H), 7.83 (d, J = 8.2 Hz, 1H), 7.75 - 7.53 (m, 3H), 6.46 (s, 1H), 4.41 - 4.28 (m, 2H), 4.25 - 4.12 (m, 2H); ^{13}C NMR (100 MHz, $CDCl_3$) δ ppm 154.57, 141.45, 136.68,

129.99, 127.29, 127.16, 126.28, 125.14, 121.88, 103.46, 65.45; HRMS (ESI) Calcd. for $C_{12}H_{12}NO_2$ [(M+H)$^+$] 202.0868, found 202.0859.

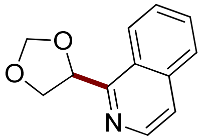

1-(1,3-dioxolan-4-yl) isoquinoline (±4d minor isomer): According to the general procedure, 1,3-dioxolane (370 mg, 5.00 mmol, 10.0 equiv.), isoquinoline (65 mg, 0.50 mmol, 1.0 equiv.), $(NH_4)_2S_2O_8$ (342 mg, 1.50 mmol, 3.0 equiv.), N-Hydroxy succinimide (115 mg, 1.00 mmol, 2.0 equiv.) and 1.5 mL of H_2O were used. After 24 hours, the reaction mixture was subjected to the workup protocol outlined in the general procedure and purified by flash chromatography (10% ethyl acetate/petroleum ether) to provide the title compound as a colourless oil (10 mg, 10% yield). ^{1}H NMR (400 MHz, $CDCl_3$) δ ppm 8.50 (d, J = 5.7 Hz, 1H), 8.36 (d, J = 8.6 Hz, 1H), 7.86 (d, J = 8.1 Hz, 1H), 7.75 - 7.68 (m, 1H), 7.65 (dd, J = 11.2, 3.9 Hz, 2H), 5.80 (t, J = 6.7 Hz, 1H), 5.29 (s, 1H), 5.20 (s, 1H), 4.57 (dd, J = 8.1, 6.5 Hz, 1H), 4.42 (dd, J = 8.0, 7.2 Hz, 1H); ^{13}C NMR (100 MHz, $CDCl_3$) δ ppm 156.29, 141.44, 136.59, 130.12 (s), 127.52, 127.42, 126.85, 124.93, 121.21, 96.00, 76.15, 68.43; HRMS (ESI) Calcd. for $C_{12}H_{12}NO_2$ [(M+H)$^+$] 202.0868, found 202.0859.

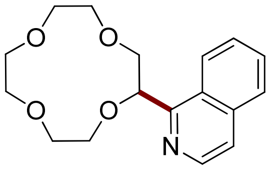

1-(1,4,7,10-Tetraoxacyclododecan-2-yl)isoquinoline (±4e): According to the general procedure, 1,4,7,10-tetraoxacyclododecane (440 μL, 2.50 mmol, 5.0 equiv.), isoquinoline (65 mg, 0.50 mmol, 1.0 equiv.), $(NH_4)_2S_2O_8$ (342 mg, 1.50 mmol, 3.0 equiv.), N-Hydroxy succinimide (115 mg, 1.00 mmol, 2.0 equiv.) and 1.5 mL of H_2O were used. After 24 hours, the reaction mixture was subjected to the workup protocol outlined in the general procedure and purified by flash chromatography (10% methanol/ dichloromethane) to provide the title compound as a colourless oil (114 mg, 75% yield). ^{1}H NMR (400 MHz, $CDCl_3$) δ ppm 8.55 (d, J = 8.6 Hz, 1H), 8.48 (d, J = 5.7 Hz, 1H), 7.80 (d, J = 8.2 Hz, 1H), 7.68 - 7.62 (m, 1H), 7.61 - 7.53 (m, 2H), 5.64 (dd, J = 9.5, 2.4 Hz, 1H), 4.14 - 4.08 (m, 1H), 3.95 - 3.67 (m, 12H), 3.55 - 3.47 (m, 1H); ^{13}C NMR (100 MHz, $CDCl_3$) δ ppm 159.34, 141.30, 136.24, 129.56, 127.04, 126.84, 126.33, 125.03, 120.24, 78.85, 68.71, 67.69, 53.29, 30.48, 25.90, 25.36, 18.91; HRMS (ESI) Calcd. for $C_{17}H_{22}NO_4$ [(M+H)$^+$] 304.1549, found 304.1540.

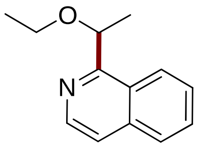

1-(1-Ethoxyethyl)isoquinoline (±4f): According to the general procedure, diethyl

ether (370 mg, 5.00 mmol, 10.0 equiv.), isoquinoline (65 mg, 0.50 mmol, 1.0 equiv.) $(NH_4)_2S_2O_8$ (342 mg, 1.50 mmol, 3.0 equiv.), N-Hydroxy succinimide (115 mg, 1.00 mmol, 2.0 equiv.) and 1.5 mL of $MeCN/H_2O$ (1: 1) were used. After 24 hours, the reaction mixture was subjected to the workup protocol outlined in the general procedure and purified by flash chromatography (10% ethyl acetate/petroleum ether) to provide the title compound as a colourless oil (65 mg, 65% yield). ^1H NMR (400 MHz, $CDCl_3$) δ ppm 8.69 (d, J = 8.6 Hz, 1H), 8.46 (d, J = 5.7 Hz, 1H), 7.84 (d, J = 8.1 Hz, 1H), 7.67 (t, J = 7.5 Hz, 1H), 7.58 (t, J = 7.9 Hz, 2H), 5.17 (q, J = 6.7 Hz, 1H), 3.53 - 3.47 (m, 1H), 3.43 - 3.36 (m, 1H), 1.70 (d, J = 6.8 Hz, 3H), 1.19 (t, J = 7.0 Hz, 3H); ^{13}C NMR (100 MHz, $CDCl_3$) δ ppm 161.79, 141.52, 136.81, 129.87, 127.39, 126.85, 126.05, 125.57, 120.50, 80.09, 64.47, 21.63, 15.45; HRMS (ESI) Calcd. for $C_{13}H_{16}NO$ $[(M+H)^+]$ 202.1232, found 202.1239.

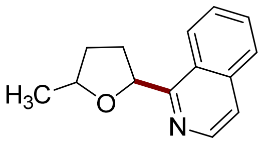

1-(5-Methyltetrahydrofuran-2-yl) isoquinoline (±4g major isomer): According to the general procedure, 2-methyltetrahydrofuran (430 mg, 5.00 mmol, 10.0 equiv.), isoquinoline (65 mg, 0.50 mmol, 1.0 equiv.), $(NH_4)_2S_2O_8$ (342 mg, 1.50 mmol, 3.0 equiv.), N-Hydroxy succinimide (115 mg, 1.00 mmol, 2.0 equiv.) and 1.5 mL of $MeCN/H_2O$ (1: 1) were used. After 24 hours, the reaction mixture was subjected to the workup protocol outlined in the general procedure and purified by flash chromatography (10% ethyl acetate/petroleum ether) to provide the title compound as a colourless oil (59mg, 55% yield; diastereoisomers, d.r. 1.7: 1). ^1H NMR (400 MHz, $CDCl_3$) δ ppm 8.48 (d, J = 5.7 Hz, 1H), 8.41(8.33) (d, J = 8.4 Hz, 1H); 7.79 (d, J = 8.0 Hz, 1H), 7.64 (ddd, J = 8.1, 6.9, 1.2 Hz, 1H), 7.61 - 7.52 (m, 2H), 5.61(5.86) (t, J = 7.3 Hz, 1H), 4.30 - 4.22(4.49 - 4.40) (m, 1H), 2.67 - 2.51 (m, 1H), 2.48 - 2.33 (m, 1H), 2.32 - 2.17 (m, 1H), 1.81 - 1.67 (m, 1H), 1.38(1.37) (d, J = 6.1 Hz, 3H); ^{13}C NMR (101 MHz, $CDCl_3$) δ ppm 160.02 (159.06), 141.49, 136.52 (136.43), 129.70 (129.68), 127.20 (127.13), 126.99 (126.95), 126.47, 125.58 (125.20), 120.53 (120.31), 79.88 (78.37), 76.73 (75.96), 33.99 (33.10), 31.36 (30.29), 21.32 (21.06). HRMS (ESI) m/z calculated for $C_{14}H_{16}NO$ $[(M+H)^+]$ 214.1232, found 214.1237.

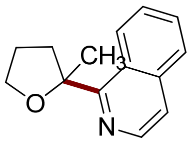

1-(2-methyltetrahydrofuran -2-yl)isoquinoline (±4g minor isomer): According to the general procedure, 2-methyltetrahydrofuran (430 mg, 5.00 mmol, 10.0 equiv.), isoquinoline (65 mg, 0.50 mmol, 1.0 equiv.), $(NH_4)_2S_2O_8$ (342 mg, 1.50 mmol, 3.0 equiv.), N-Hydroxy succinimide (115 mg, 1.00 mmol, 2.0 equiv.) and 1.5 mL of $MeCN/H_2O$ (1: 1) were used. After 24 hours, the reaction mixture was subjected to the workup protocol outlined in the general procedure and purified by flash

chromatography (10% ethyl acetate/petroleum ether) to provide the title compound as a colourless oil (29mg, 27% yield). ^{1}H NMR (400 MHz, $CDCl_3$) δ ppm 9.01 (d, J = 8.6 Hz, 1H), 8.42 (d, J = 5.6 Hz, 1H), 7.77 (d, J = 8.0 Hz, 1H), 7.65 - 7.58 (m, 1H), 7.56 - 7.50 (m, 2H), 4.14 - 3.98 (m, 1H), 3.82 - 3.68 (m, 1H), 3.32 - 3.17 (m, 1H), 2.11 - 1.97 (m, 2H), 1.93 - 1.81 (m, 1H), 1.76 (s, 3H); ^{13}C NMR (100 MHz, $CDCl_3$) δ ppm 163.68, 140.44, 137.25, 129.32, 128.00, 127.26, 126.27, 126.10, 120.33, 88.28, 67.71, 37.56, 28.29, 25.03; HRMS (ESI) Calcd. for $C_{14}H_{16}NO$ [(M+H)$^+$] 214.1232, found 214.1237.

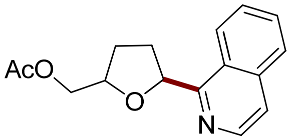

(5-(Isoquinolin-1-yl)tetrahydrofuran-2-yl)methyl acetate (±4h): According to the general procedure, (tetrahydrofuran-2-yl)methyl acetate (720 mg, 5.00 mmol, 10.0 equiv.), isoquinoline (65 mg, 0.50 mmol, 1.0 equiv.), $(NH_4)_2S_2O_8$ (342 mg, 1.50 mmol, 3.0 equiv.), N-Hydroxy succinimide (115 mg, 1.00 mmol, 2.0 equiv.) and 1.5 mL of H_2O were used. After 24 hours, the reaction mixture was subjected to the workup protocol outlined in the general procedure and purified by flash chromatography (20% ethyl acetate/petroleum ether) to provide the title compound (diastereoisomers, d.r. 2.2: 1) as a colourless oil (95 mg, 70% yield); major diastereoisomer: ^{1}H NMR (400 MHz, $CDCl_3$) δ ppm 8.47(8.45) (d, J = 5.7 Hz, 1H), 8.30(8.39) (d, J = 8.5 Hz, 1H), 7.82 (d, J = 7.8 Hz, 1H), 7.69 - 7.64 (m, 1H), 7.63 - 7.55 (m, 2H), 5.87(5.74) (t, J = 6.9 Hz, 1H), 4.62 - 4.57(4.44 - 4.38) (m, 1H), 4.30 - 4.20(4.18 - 4.14) (m, 2H), 2.58 - 2.51(2.69 - 2.60) (m, 1H), 2.49 - 2.37 (m, 1H), 2.35 - 2.16 (m, 1H), 2.11(2.00) (s, 3H), 1.95 - 1.84 (m, 1H); ^{13}C NMR (100 MHz, $CDCl_3$) δ ppm 171.16(171.02.9), 159.03(158.64), 141.46(141.37), 136.54(136.47), 129.87(129.81), 127.29(127.21), 127.17(127.04), 126.75(126.45), 125.50(125.08), 120.75(120.64), 80.32(79.10), 77.96(77.20), 66.53(66.53), 30.94(29.91), 28.26(28.09), 20.97(20.84);. HRMS (ESI) Calcd. for $C_{16}H_{18}NO_3$ [(M+H)$^+$] 272.1287, found 272.1289.

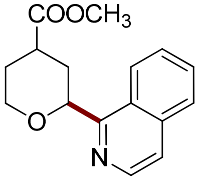

Methyl 2-(isoquinolin-1-yl)tetrahydro-2H-pyran-4-carboxylate (±4i): According to the general procedure, methyl tetrahydro-2H-pyran-4-carboxylate (720 mg, 5.00 mmol, 10.0 equiv.), isoquinoline (65 mg, 0.50 mmol, 1.0 equiv.), $(NH_4)_2S_2O_8$ (342 mg, 1.50 mmol, 3.0 equiv.), N-Hydroxy succinimide (115 mg, 1.00 mmol, 2.0 equiv.) and 1.5 mL of H_2O were used. After 24 hours, the reaction mixture was subjected to the workup protocol outlined in the general procedure and purified by flash chromatography (20% ethyl acetate/petroleum ether) to provide the title compound as a colourless oil (84 mg, 62% yield). ^{1}H NMR (400 MHz, $CDCl_3$) δ ppm 8.54 (d, J =

5.7 Hz, 1H), 8.37 (d, J = 8.4 Hz, 1H), 7.82 (d, J = 7.6 Hz, 1H), 7.70 - 7.65 (m, 1H), 7.63 (ddd, J = 8.2, 7.0, 1.4 Hz, 1H), 7.58 (d, J = 5.7 Hz, 1H), 5.57 (dd, J = 10.1, 2.6 Hz, 1H), 4.09 - 4.04 (m, 1H), 3.91 - 3.84 (m, 1H), 3.84 (s, 3H), 3.18 - 3.13 (m, 1H), 2.47 - 2.42 (m, 1H), 2.35 - 2.24 (m, 1H), 2.15 - 2.10 (m, 2H); ^{13}C NMR (100 MHz, $CDCl_3$) δ ppm 175.35, 158.96, 141.70, 136.36, 129.81, 127.27, 127.24, 125.97, 124.86, 120.50, 73.93, 65.42, 51.90, 37.07, 31.56, 27.28; HRMS (ESI) Calcd. for $C_{16}H_{18}NO_3$ [(M+H)$^+$] 272.1287, found 272.1282.

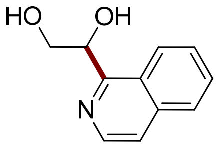

1-(Isoquinolin-1-yl)ethane-1,2-diol (±4j): According to the general procedure, 2,2-dimethyl-1,3-dioxolane (510 mg, 5.00 mmol, 10.0 equiv.), isoquinoline (65 mg, 0.50 mmol, 1.0 equiv.), $(NH_4)_2S_2O_8$ (342 mg, 1.50 mmol, 3.0 equiv.), N-Hydroxy succinimide (115 mg, 1.00 mmol, 2.0 equiv.) and 1.5 mL of MeCN/H_2O (1: 1) were used. After 24 hours, the reaction mixture was subjected to the workup protocol outlined in the general procedure and purified by flash chromatography (ethyl acetate) to provide the title compound as a colourless oil (70mg, 74% yield); ^{1}H NMR (400 MHz, $CDCl_3$) δ ppm 8.45 (d, J = 5.7 Hz, 1H), 8.12 (d, J = 8.5 Hz, 1H), 7.88 (d, J = 8.2 Hz, 1H), 7.73 (ddd, J = 8.2, 7.0, 1.1 Hz, 1H), 7.69 - 7.59 (m, 2H), 5.55 (dd, J = 6.3, 3.2 Hz, 1H), 4.10 (dd, J = 11.5, 3.2 Hz, 1H), 3.76 (dd, J = 11.5, 6.3 Hz, 1H); ^{13}C NMR (100 MHz, $CDCl_3$) δ ppm 157.69, 140.33, 136.47, 130.54, 127.74, 127.56, 125.33, 124.01, 121.10, 70.51, 67.60; HRMS (ESI) Calcd. for $C_{11}H_{12}NO_2$ [(M+H)$^+$] 190.0868, found 190.0879.

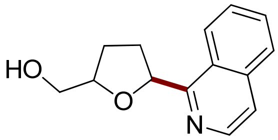

(5-(Isoquinolin-1-yl)tetrahydrofuran-2-yl)methanol (±4k): According to the general procedure, (tetrahydrofuran-2-yl)methanol (510 mg, 5.00 mmol, 10.0 equiv.), isoquinoline (65 mg, 0.50 mmol, 1.0 equiv.), $(NH_4)_2S_2O_8$ (342 mg, 1.50 mmol, 3.0 equiv.), N-Hydroxy succinimide (115 mg, 1.00 mmol, 2.0 equiv.) and 1.5 mL of H_2O were used. After 24 hours, the reaction mixture was subjected to the workup protocol outlined in the general procedure and purified by flash chromatography (ethyl acetate) to provide the title compound (diastereoisomers, d.r. 2: 1) as a colourless oil (84 mg, 73% yield). Major: ^{1}H NMR (400 MHz, $CDCl_3$) δ ppm 8.43 (d, J = 5.8 Hz, 1H), 8.09 (d, J = 8.4 Hz, 1H), 7.86 (d, J = 8.2 Hz, 1H), 7.76 - 7.68 (m, 1H), 7.67 - 7.57 (m, 2H), 6.02 (dd, J = 8.5, 2.8 Hz, 1H), 4.50 - 4,45 (m, 1H), 4.24 (dd, J = 11.9, 2.4 Hz, 1H), 3.68 (m, 1H), 2.74 - 2.59 (m, 1H), 2.34 - 2.18 (m, 2H), 2.04 - 1.93 (m, 1H); ^{13}C NMR (100 MHz, $CDCl_3$) δ ppm 162.17, 140.50, 136.30, 130.36, 127.63, 127.49, 125.15, 123.76, 120.59, 82.10, 76.45, 63.70, 34.32, 25.31. Minor: ^{1}H NMR (400 MHz, $CDCl_3$) δ ppm 8.49 (d, J = 5.7 Hz, 1H), 8.28 (d, J = 8.5 Hz, 1H), 7.83 (d, J = 8.2 Hz, 1H),

7.72 - 7.64 (m, 1H), 7.60 - 7.57 (m, 2H), 5.85 (t, J = 7.1 Hz, 1H), 4.53 - 4.47 (m, 1H), 3.83 (dd, J = 11.6, 3.3 Hz, 1H), 3.70 - 3.66 (m, 1H), 2.57 - 2.43 (m, 2H), 2.26 - 2.18 (m, 2H), 2.01 - 1.92 (m, 1H); ^{13}C NMR (100 MHz, $CDCl_3$) δ ppm 159.44, 141.47, 136.50, 129.91, 127.38, 127.17, 126.28, 124.97, 120.61, 80.32, 79.19, 65.02, 31.77, 27.70. HRMS (ESI) Calcd. for $C_{14}H_{16}NO_2$ [$(M+H)^+$] 230.1181, found 230.1185.

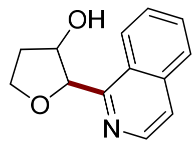

2-(Isoquinolin-1-yl)tetrahydrofuran -3-ol (±4l major isomer): According to the general procedure, tetrahydrofuran-2-ol (440 mg, 5.00 mmol, 10.0 equiv.), isoquinoline (65 mg, 0.50 mmol, 1.0 equiv.), $(NH_4)_2S_2O_8$ (342 mg, 1.50 mmol, 3.0 equiv.), N-Hydroxy succinimide (115 mg, 1.00 mmol, 2.0 equiv.) and 1.5 mL of H_2O were used. After 24 hours, the reaction mixture was subjected to the workup protocol outlined in the general procedure and purified by flash chromatography (ethyl acetate) to provide the title mixture as a colourless oil (50 mg, 47% yield; diastereoisomers, d.r. 4.9: 1). ^{1}H NMR (400 MHz, $CDCl_3$) δ ppm 8.45(8.39) (d, J = 5.6 Hz, 1H), 8.39 - 8.35 (m, 1H), 7.84 - 7.80 (m, 1H), 7.74 - 7.66 (m, 1H), 7.66 - 7.57 (m, 2H), 6.95 (br, 1H), 6.11(5.27) (dd, J = 8.8, 1.6 Hz, 1H), 4.57 - 4.55(4.95 - 4.93) (m, 1H), 4.42 - 3.97 (m, 2H), 2.68 - 2.24 (m, 2H); ^{13}C NMR (100 MHz, $CDCl_3$) δ ppm 160.06(157.72), 140.71(139.73), 136.69(136.44), 130.51(130.43), 127.75 (127.67), 127.27 (127.10), 126.62 (125.80), 124.60, 121.26 (120.99), 78.67 (78.35), 75.21 (73.28), 72.32 (67.58), 38.95 (34.97). HRMS (ESI) Calcd. for $C_{13}H_{14}NO_2$ [$(M+H)^+$] 216.1025, found 216.1031.

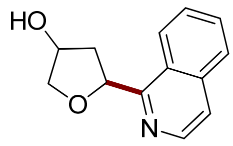

5-(Isoquinolin -1-yl) tetrahydrofuran-2-ol (±4l minor isomer): According to the general procedure, tetrahydrofuran-2-ol (440 mg, 5.00 mmol, 10.0 equiv.), isoquinoline (65 mg, 0.50 mmol, 1.0 equiv.), $(NH_4)_2S_2O_8$ (342 mg, 1.50 mmol, 3.0 equiv.), N-Hydroxy succinimide (115 mg, 1.00 mmol, 2.0 equiv.) and 1.5 mL of H_2O were used. After 24 hours, the reaction mixture was subjected to the workup protocol outlined in the general procedure and purified by flash chromatography (ethyl acetate) to provide the title mixture as a colourless oil (25 mg, 23% yield). ^{1}H NMR (400 MHz, $CDCl_3$) δ ppm 8.45 (t, J = 7.5 Hz, 2H), 7.81 (d, J = 8.1 Hz, 1H), 7.68 (t, J = 7.5 Hz, 1H), 7.60 (dd, J = 13.9, 6.8 Hz, 2H), 5.43 (d, J = 4.4 Hz, 1H), 4.96 - 4.92 (m, 1H), 4.32 - 4.16 (m, 2H), 2.47 - 2.38 (m, 1H), 2.14 - 2.09 (m, 1H). ^{13}C NMR (100 MHz, $CDCl_3$) δ ppm 158.45, 141.40, 136.50, 130.00, 127.35, 127.26, 126.90, 125.19, 120.85, 77.40, 76.18, 72.80, 40.15. HRMS (ESI) Calcd. for $C_{13}H_{14}NO_2$ [$(M+H)^+$] 216.1025, found 216.1031.

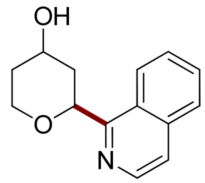

2-(Isoquinolin-1-yl)tetrahydro-2H-pyran-4-ol (±4m): According to the general procedure, tetrahydro-2H-pyran-4-ol (510 mg, 5.00 mmol, 10.0 equiv.), isoquinoline (65 mg, 0.50 mmol, 1.0 equiv.), $(NH_4)_2S_2O_8$ (342 mg, 1.50 mmol, 3.0 equiv.), N-Hydroxy succinimide (115 mg, 1.00 mmol, 2.0 equiv.) and 1.5 mL of H_2O were used. After 24 hours, the reaction mixture was subjected to the workup protocol outlined in the general procedure and purified by flash chromatography (ethyl acetate) to provide the title compound as a colourless oil (80 mg, 70% yield). ^{1}H NMR (400 MHz, $CDCl_3$) δ ppm 8.51 (d, *J* = 5.7 Hz, 1H), 8.34 (d, *J* = 8.4 Hz, 1H), 7.81 (d, *J* = 8.1 Hz, 1H), 7.69 - 7.63 (m, 1H), 7.63 - 7.55 (m, 2H), 5.76 (dd, *J* = 11.2, 2.3 Hz, 1H), 4.54 - 4.45 (m, 1H), 4.30 - 4.21 (m, 1H), 4.04 - 4.00 (m, 1H), 2.44 - 2.37 (m, 1H), 2.15 - 2.00 (m, 2H), 1.78 - 1.67 (m, 1H); ^{13}C NMR (100 MHz, $CDCl_3$) δ ppm 159.15, 141.64, 136.51, 129.85, 127.32, 127.15, 126.16, 125.05, 120.63, 72.21, 64.15, 63.34, 37.84, 32.92; HRMS (ESI) Calcd. for $C_{14}H_{16}NO_2$ $[(M+H)^+]$ 230.1181, found 230.1187.

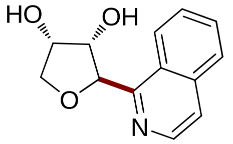

(*3S,4S*)-2-(Isoquinolin-1-yl)tetrahydrofuran-3,4-diol (±4n): According to the general procedure, 1,4-anhydroerythritol (520 mg, 5.00 mmol, 10.0 equiv.), isoquinoline (65 mg, 0.50 mmol, 1.0 equiv.), $(NH_4)_2S_2O_8$ (342 mg, 1.50 mmol, 3.0 equiv.), N-Hydroxy succinimide (115 mg, 1.00 mmol, 2.0 equiv.) and 1.5 mL of H_2O were used. After 24 hours, the reaction mixture was subjected to the workup protocol outlined in the general procedure and purified by flash chromatography (ethyl acetate) to provide the title compound as a colourless oil (87 mg, 75% yield). ^{1}H NMR (400 MHz, $CDCl_3$) δ ppm 8.46 (d, *J* = 5.8 Hz, 1H), 8.33 (d, *J* = 8.5 Hz, 1H), 7.88 (d, *J* = 8.2 Hz, 1H), 7.79 - 7.73 (m, 1H), 7.72 - 7.66 (m, 2H), 5.81 (d, *J* = 6.2 Hz, 1H), 4.84 (dd, *J* = 6.1, 5.2 Hz, 1H), 4.46 - 4.43 (m, 1H), 4.20 - 4.18 (m, 1H), 4.15 - 4.11 (m, 1H); ^{13}C NMR (100 MHz, $CDCl_3$) δ ppm 157.63, 139.60, 136.87, 131.07, 128.24, 127.84, 127.46, 125.12, 121.74, 75.60, 74.45, 73.63, 72.39; HRMS (ESI) Calcd. for $C_{13}H_{14}NO_3$ $[(M+H)^+]$ 232.0974, found 232.0970.

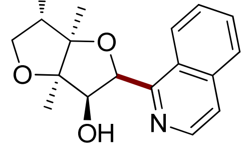

(*3R,3aR,6S,6aR*)-2-(Isoquinolin-1-yl)-3a,6a-dimethylhexahydrofuro[3,2-b]furan-

3,6-diol (±4o): According to the general procedure, isosorbide (870 mg, 5.00 mmol, 10.0 equiv.), isoquinoline (65 mg, 0.50 mmol, 1.0 equiv.), $(NH_4)_2S_2O_8$ (342 mg, 1.50 mmol, 3.0 equiv.), N-Hydroxy succinimide (115 mg, 1.00 mmol, 2.0 equiv.) and 1.5 mL of H_2O were used. After 24 hours, the reaction mixture was subjected to the workup protocol outlined in the general procedure and purified by flash chromatography (ethyl acetate) to provide the title compound as a colourless oil (98 mg, 65% yield). ^{1}H NMR (400 MHz, $CDCl_3$) δ ppm 8.47 (d, J = 5.7 Hz, 1H), 8.31 (d, J = 8.5 Hz, 1H), 7.84 (d, J = 8.2 Hz, 1H), 7.73 - 7.67 (m, 1H), 7.65 - 7.59 (m, 2H), 5.51 (d, J = 6.2 Hz, 1H), 4.96 - 4.88 (m, 1H), 4.82 (dd, J = 11.4, 5.4 Hz, 2H), 4.57 (d, J = 3.2 Hz, 1H), 4.20 (dd, J = 10.1, 3.5 Hz, 1H), 4.11 (d, J = 10.1 Hz, 1H), 2.90 (br, 2H); ^{13}C NMR (100 MHz, $CDCl_3$) δ ppm 156.77, 141.33, 136.54, 130.25, 127.54, 127.33, 126.85, 125.10, 121.18, 88.13, 83.47, 82.29, 76.85, 75.87; HRMS (ESI) Calcd. for $C_{17}H_{20}NO_4$ [$(M+H)^+$] 302.1392, found 302.1387.

References

1. E. M. Simmons, J. F. Hartwig, *Angew. Chem. Int. Ed.* **2012**, *51*, 3066-3072.
2. (a) X.-D. Feng, X.-Q. Guo, K.-Y. Qiu, *Polymer Bull.* **1987**, *18*, 19-26. (b) D. Hunkeler, A. E. Hamielec, *Macromol.* **1991**, *24*, 2160-2171. (c) X.-D. Feng, K.-Y. Qiu, *Chin. Polym. Bull.* **2005**, *4*, 23-34.
3. I. Mangion, Y.-Z. Liu, M. Reibarkh, R. T. Williamson, C. J. Welch, *J. Org. Chem.* **2016**, *81*, 6937−6944.
4. (a) T. Gieshoff, D. Schollmeyer, S. R. Waldvogel, *Angew. Chem. Int. Ed.* **2016**, *55*, 9437-9440; (b) M. V. D. Almeida, D. H. R. Barton, I. Bytheway, J. A. Ferreira, M. B. Hall, W. Liu, D. K. Taylor, L. Thomson, *J. Am. Chem. Soc.* **1995**, *117*, 4870-4874.
5. H.-Y. Yao, Y. Tang, K. Yamamoto, *Tetrahedron Lett.* **2012**, *53*, 5094-5098.
6. R. Ikeda, T. Kimura, T. Tsutsumi, S. Tamura, N. Sakai, T. Konakahara, *Bioorg. Med. Chem. Lett.* **2012**, *22*, 3506-3515.

Spectral Data for the Products

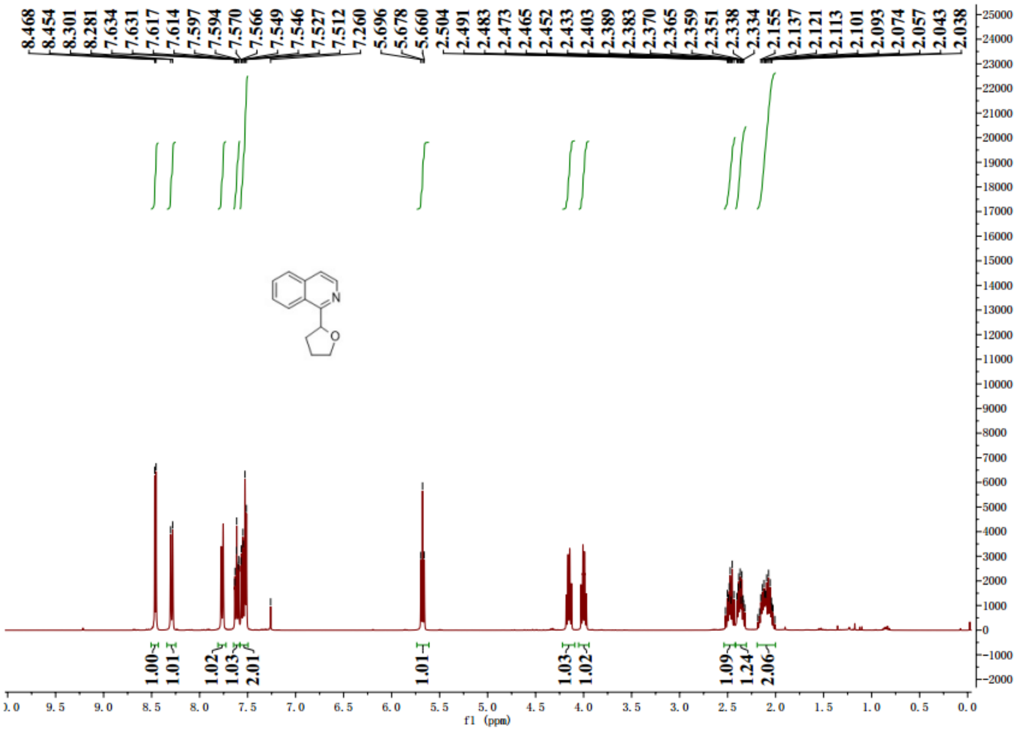

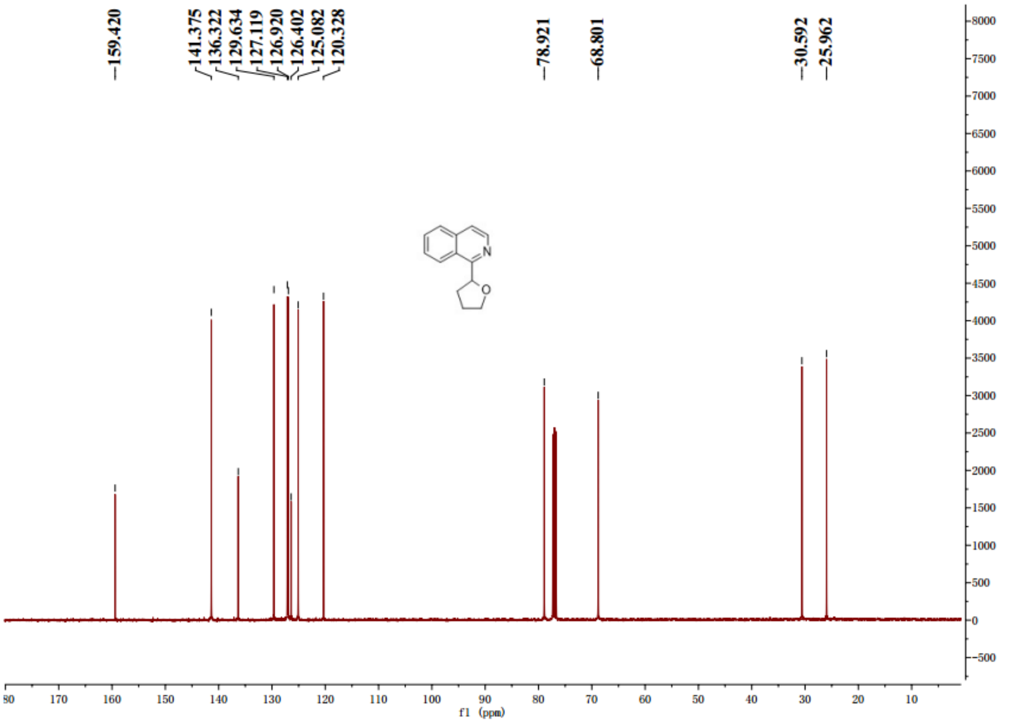

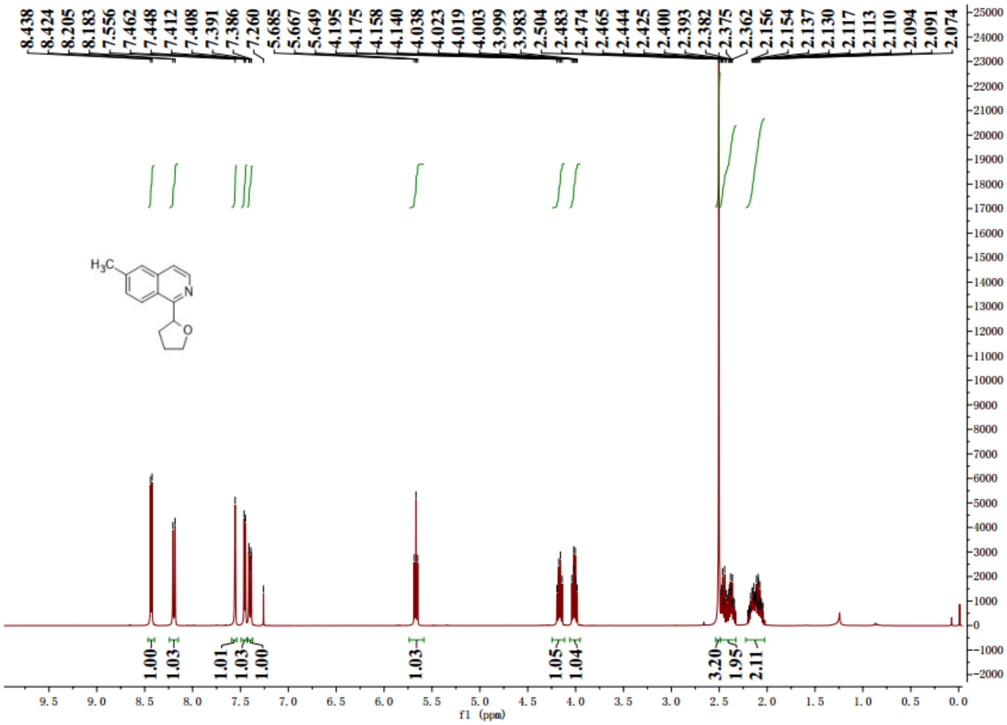

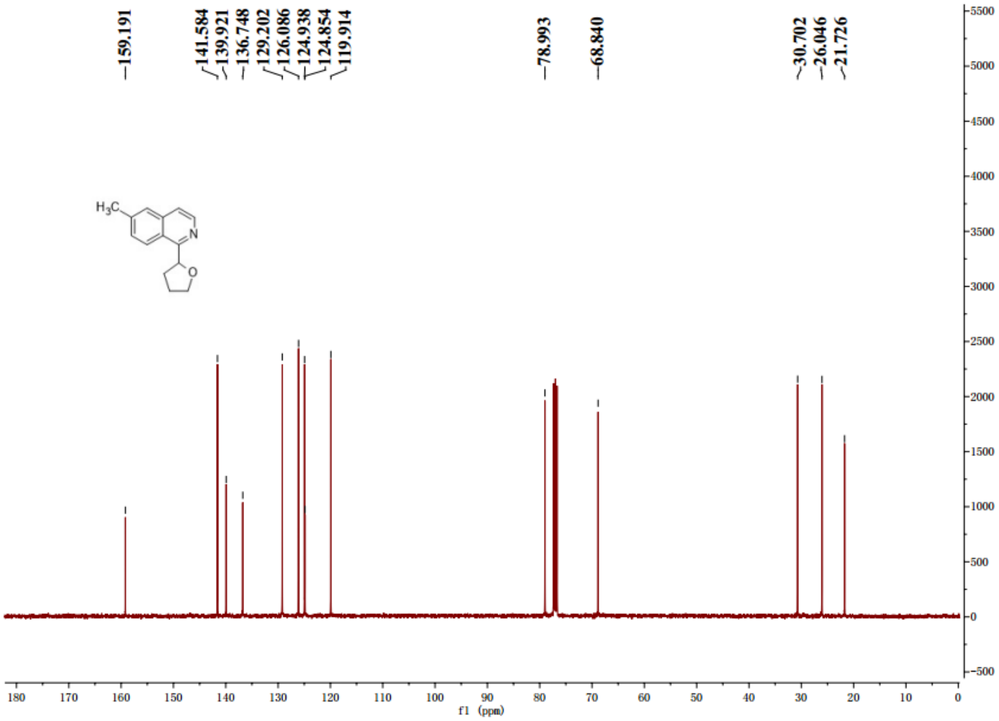

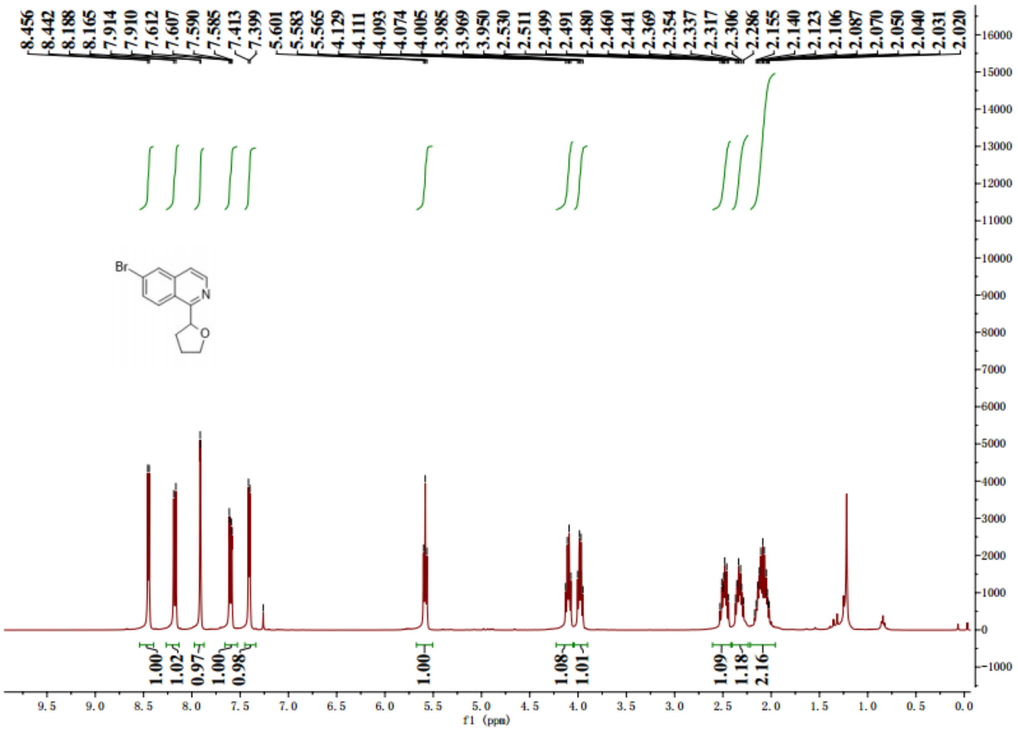
8.456
8.442
8.188
8.165
7.914
7.910
7.612
7.607
7.590
7.585
7.413
7.399
5.601
5.583
5.565
4.129
4.111
4.093
4.074
4.005
3.985
3.969
3.950
2.530
2.511
2.499
2.491
2.480
2.460
2.441
2.369
2.354
2.337
2.317
2.306
2.286
2.155
2.140
2.123
2.106
2.087
2.070
2.050
2.040
2.031
2.020
Br
N
O
1.00
1.02
0.97
1.00
0.98
1.00
1.08
1.01
1.09
1.18
2.16
f1 (ppm)

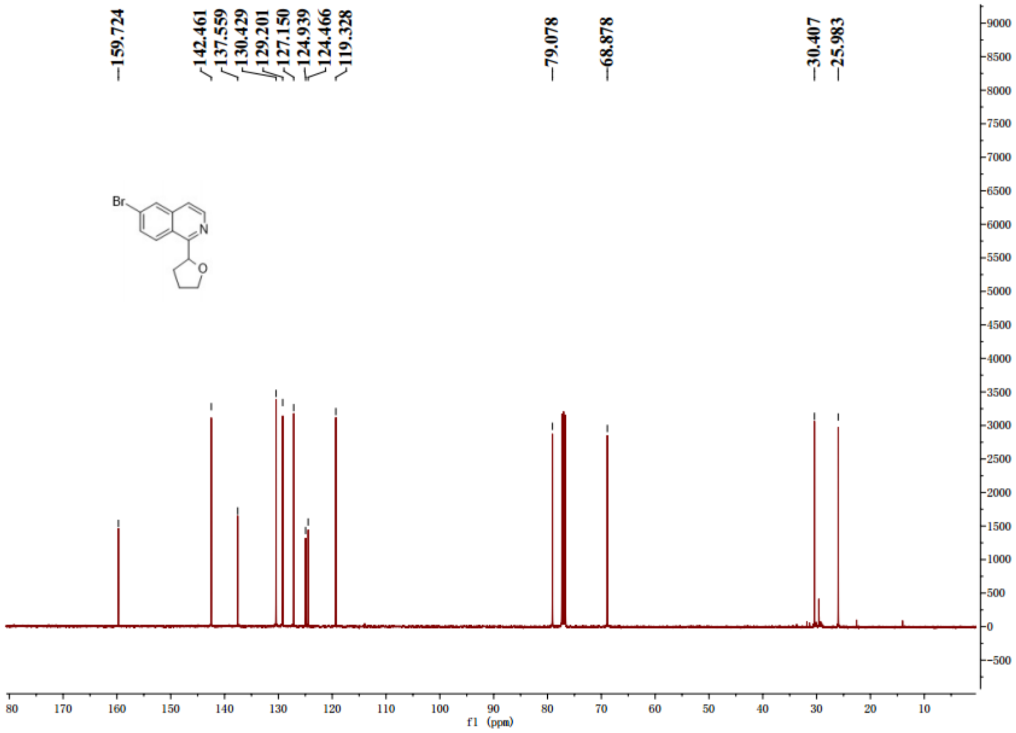
159.724
142.461
137.559
130.429
129.201
127.150
124.939
124.466
119.328
79.078
68.878
30.407
25.983
Br
N
O
f1 (ppm)

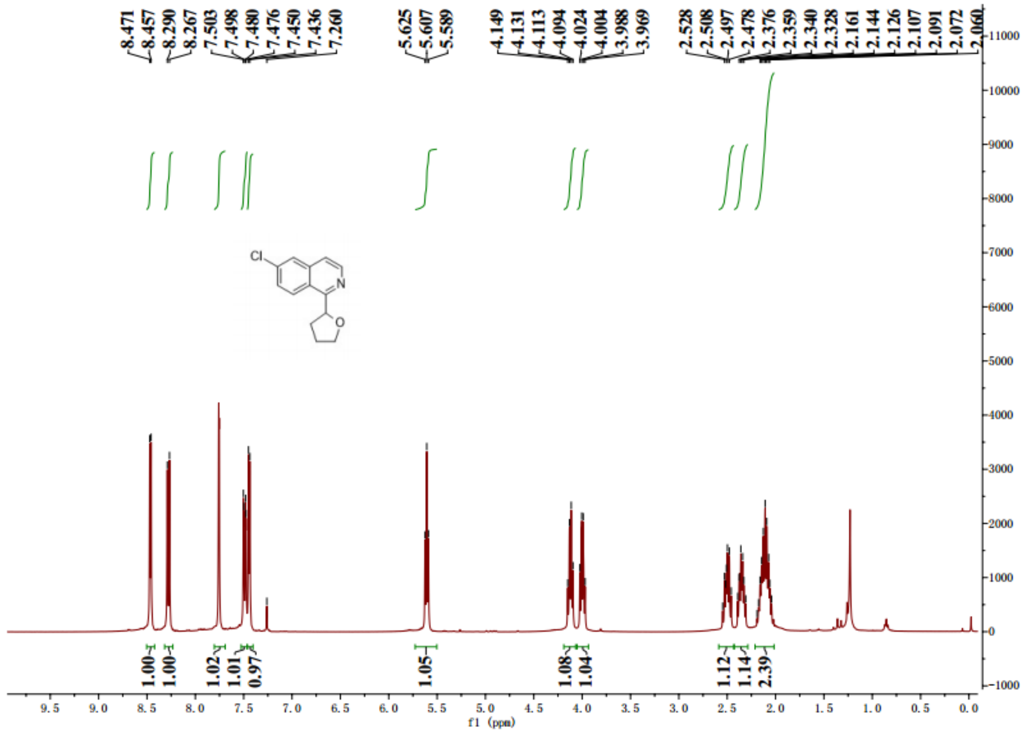

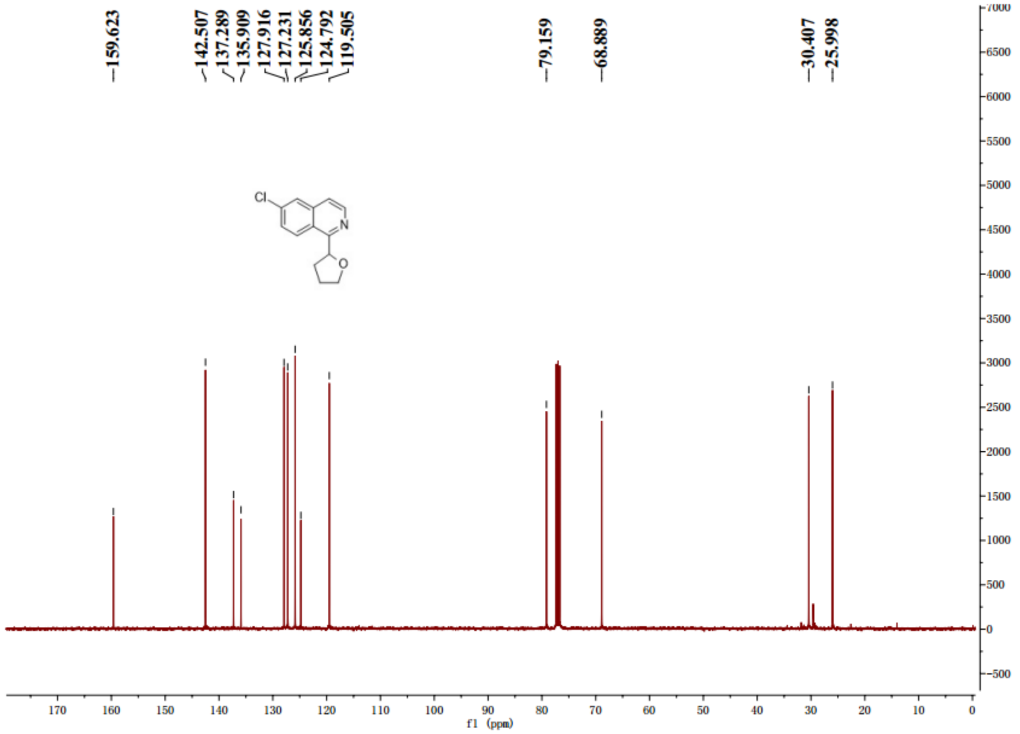

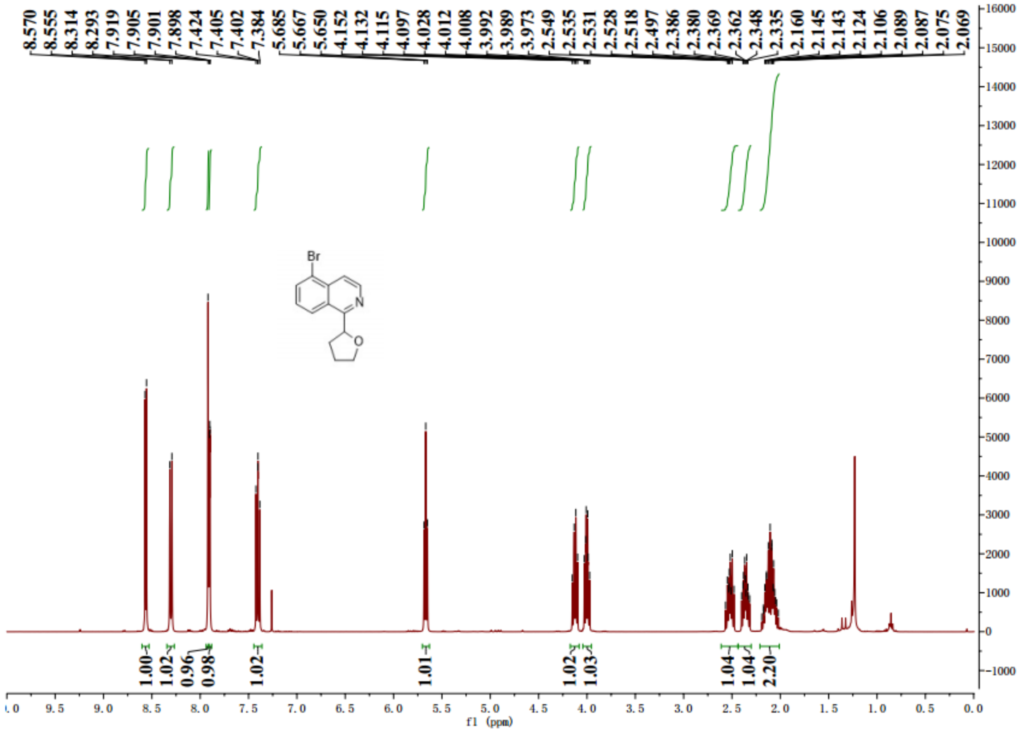

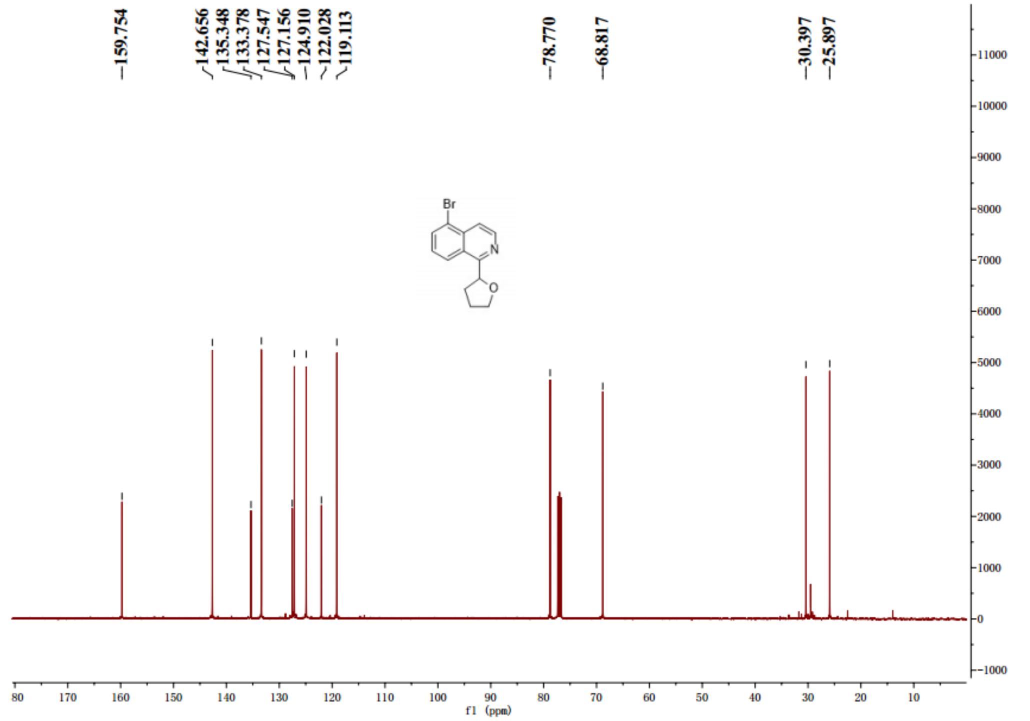

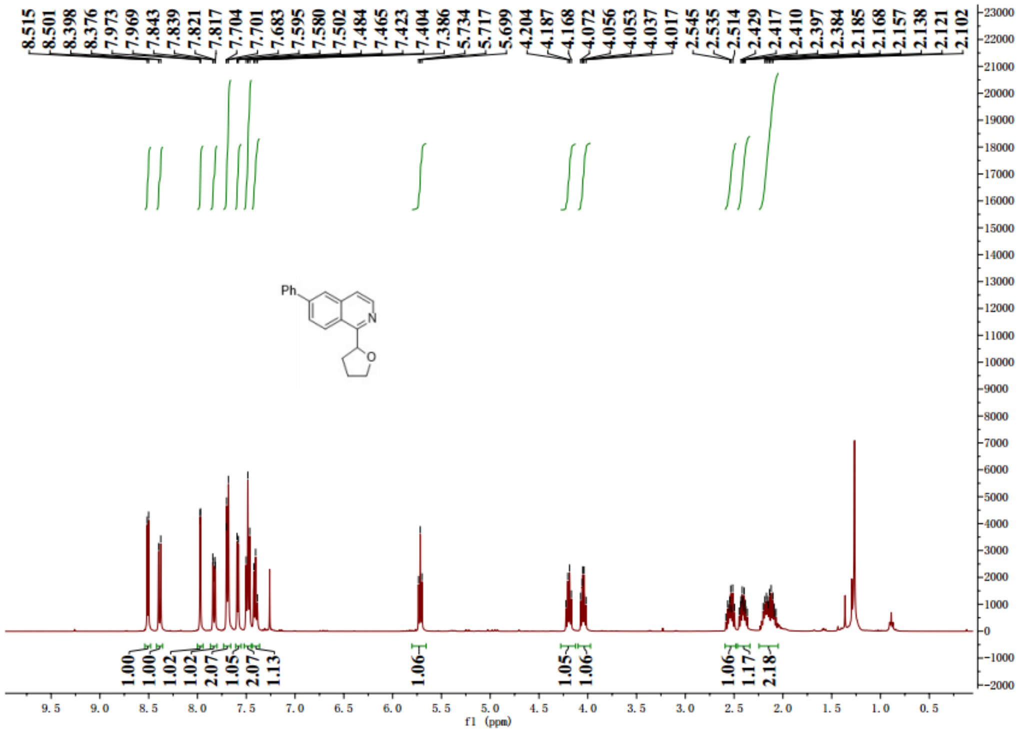

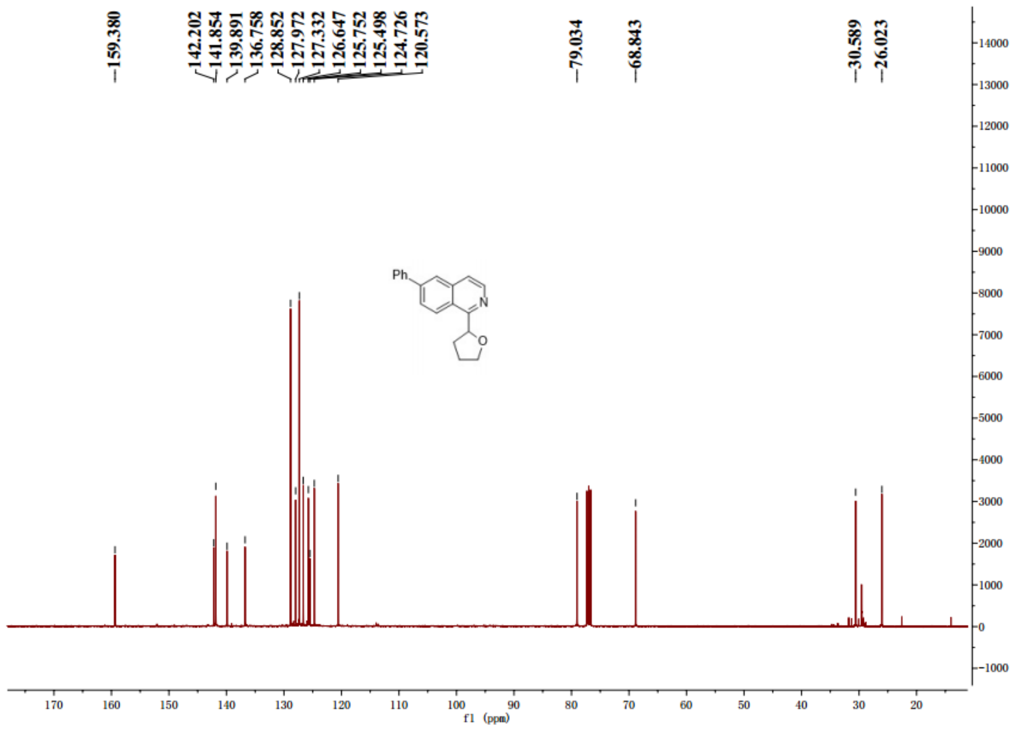

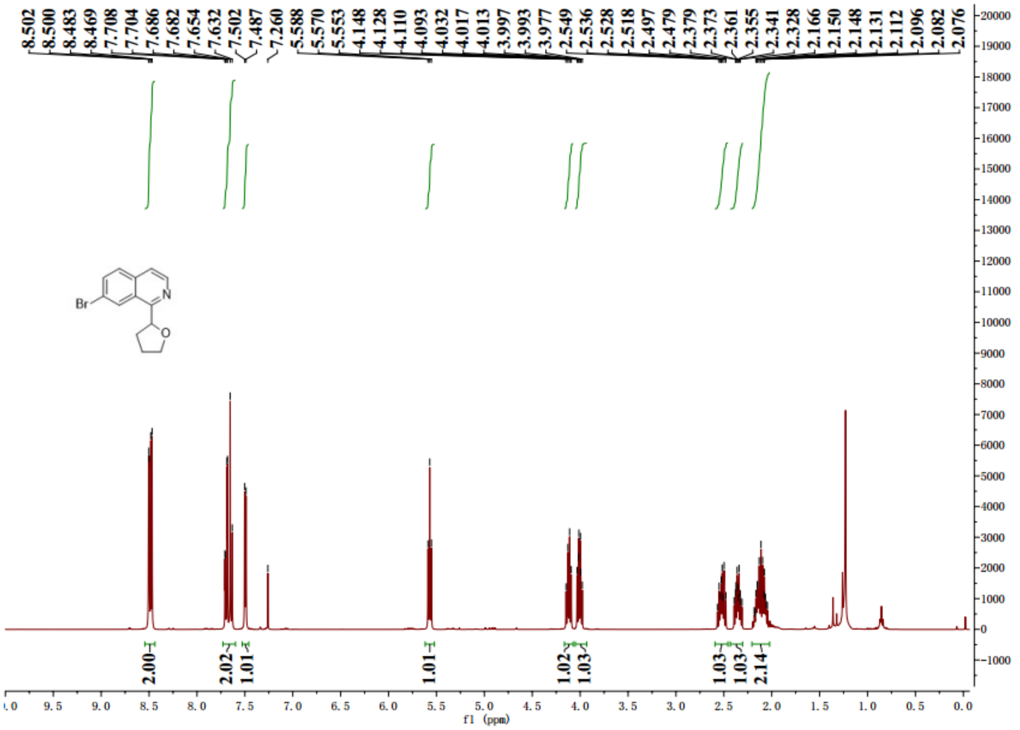

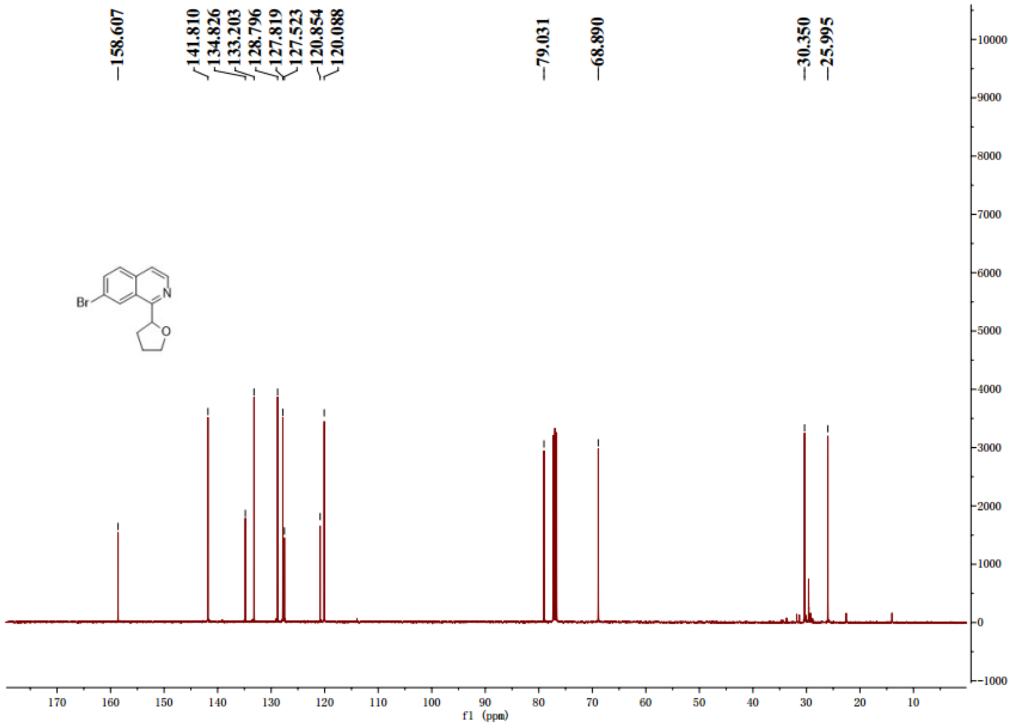

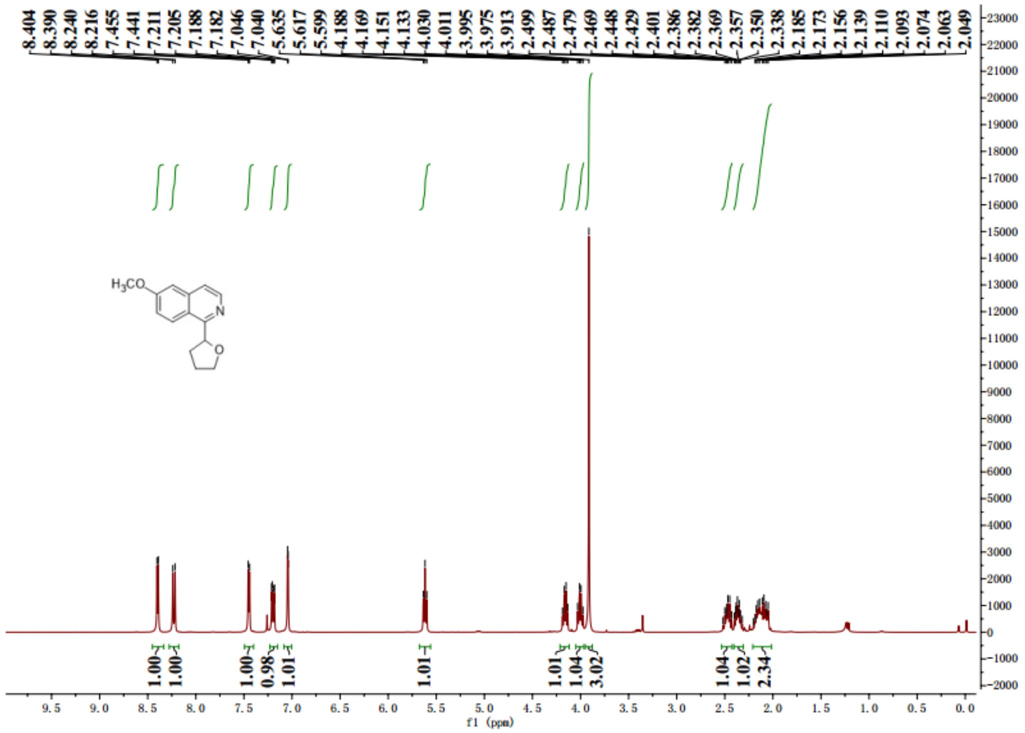

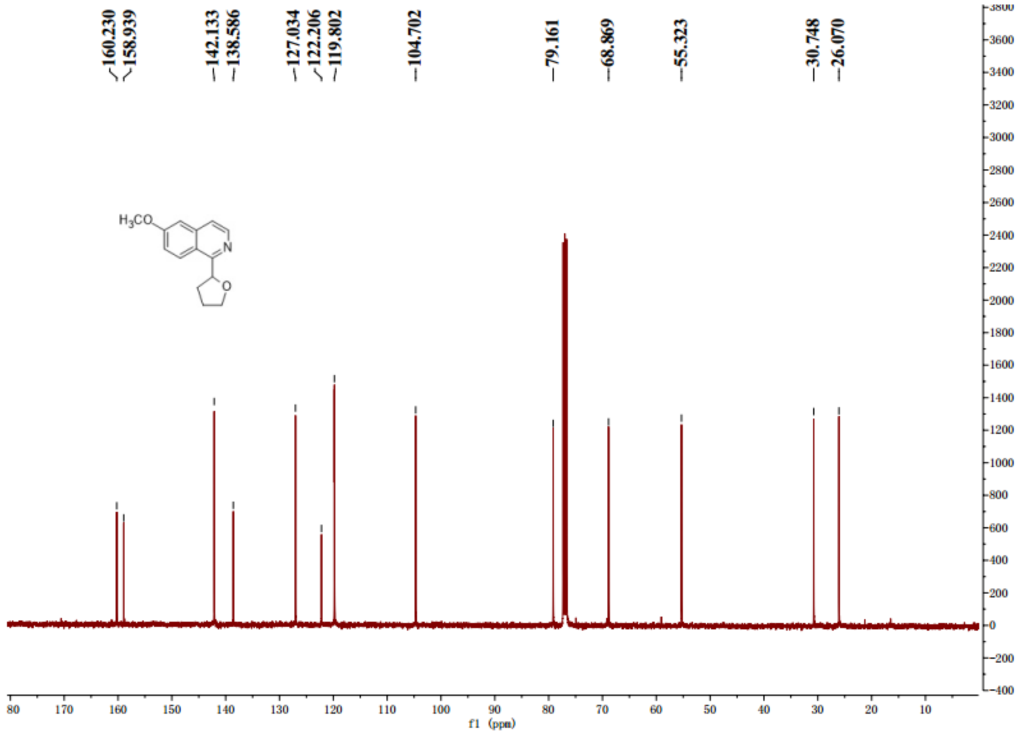

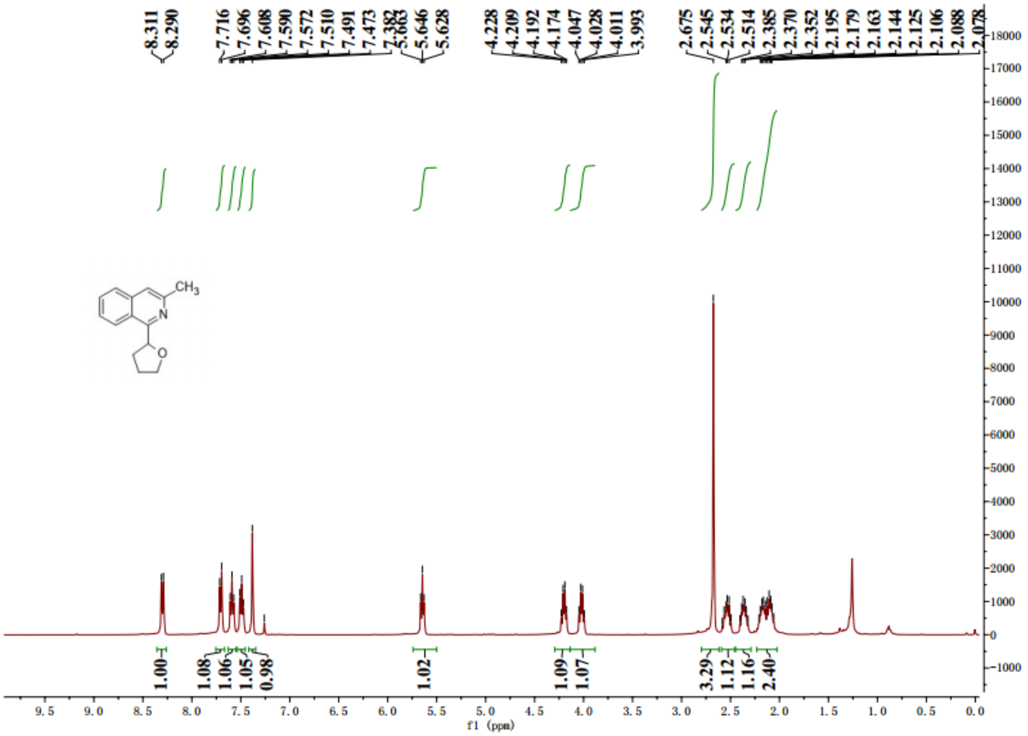
8.311
8.290
7.716
7.696
7.608
7.590
7.572
7.510
7.491
7.473
7.382
5.663
5.646
5.628
4.228
4.209
4.192
4.174
4.047
4.028
4.011
3.993
2.675
2.545
2.534
2.514
2.385
2.370
2.352
2.195
2.179
2.163
2.144
2.125
2.106
2.088
2.078
CH3
N
O
1.00
1.08
1.06
1.05
0.98
1.02
1.09
1.07
3.29
1.12
1.16
2.40
f1 (ppm)

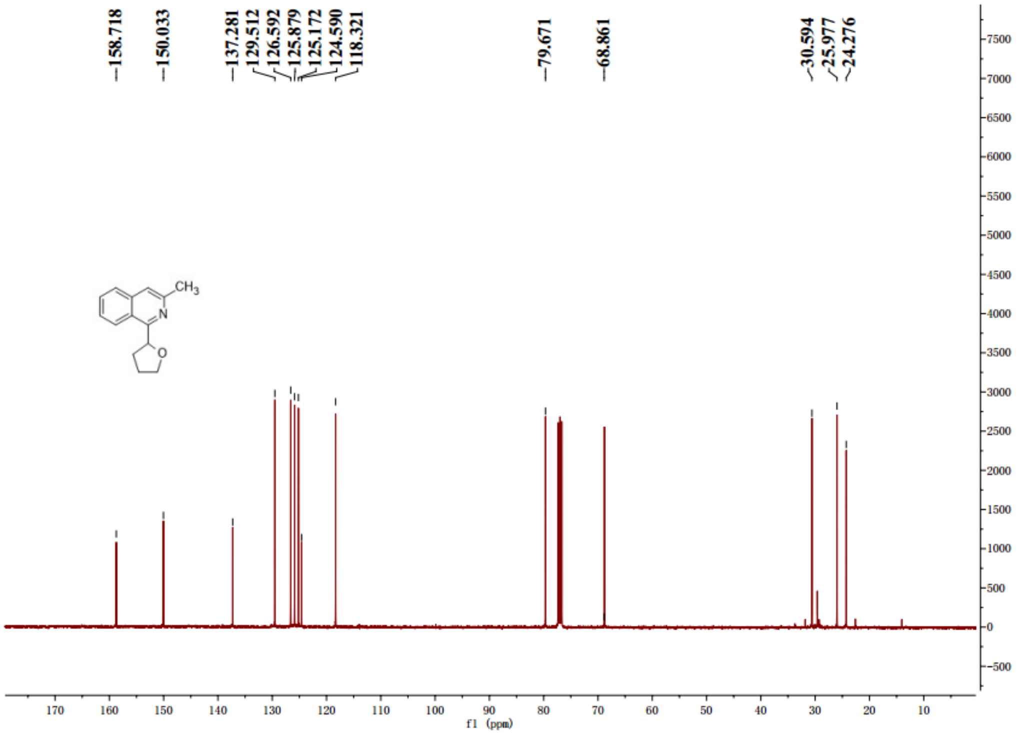
158.718
150.033
137.281
129.512
126.592
125.879
125.172
124.590
118.321
79.671
68.861
30.594
25.977
24.276
CH3
N
O
f1 (ppm)

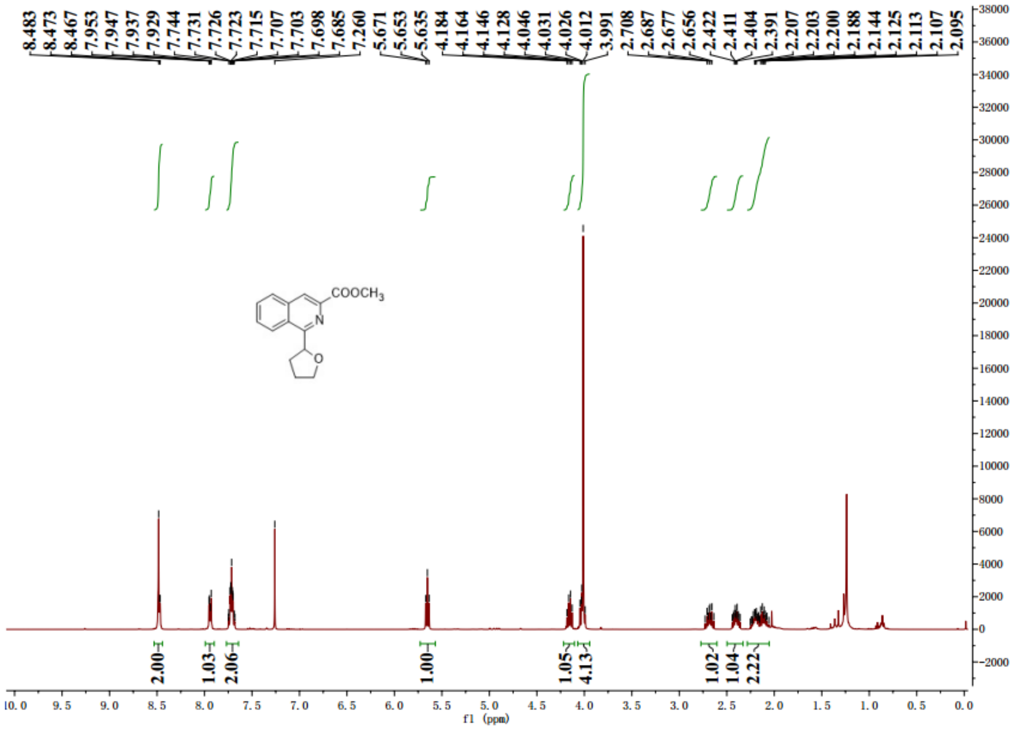

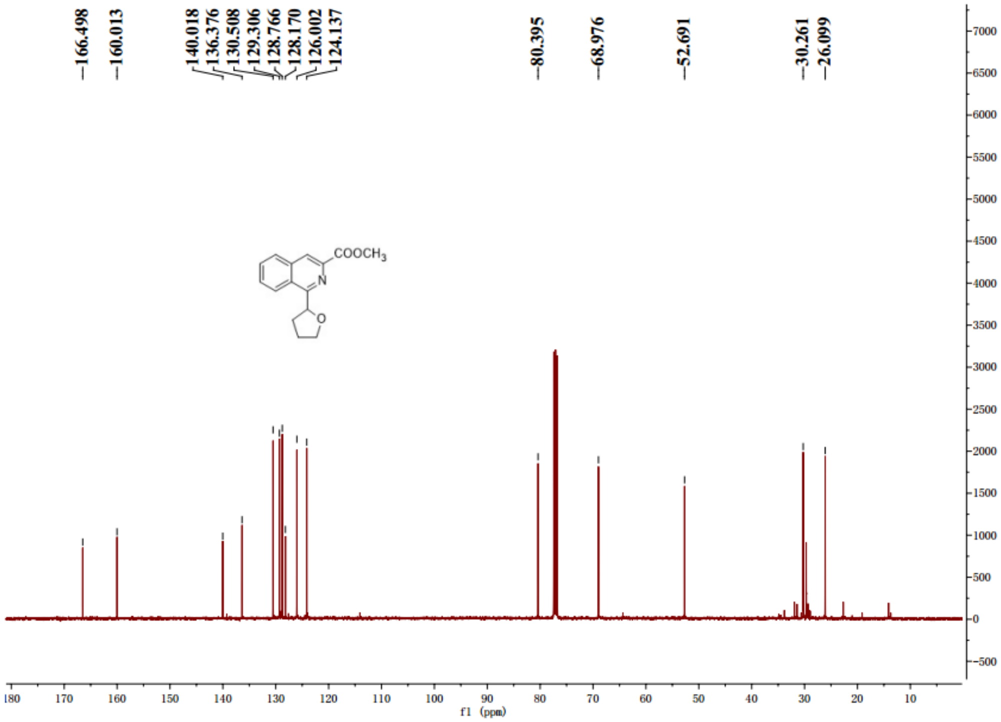

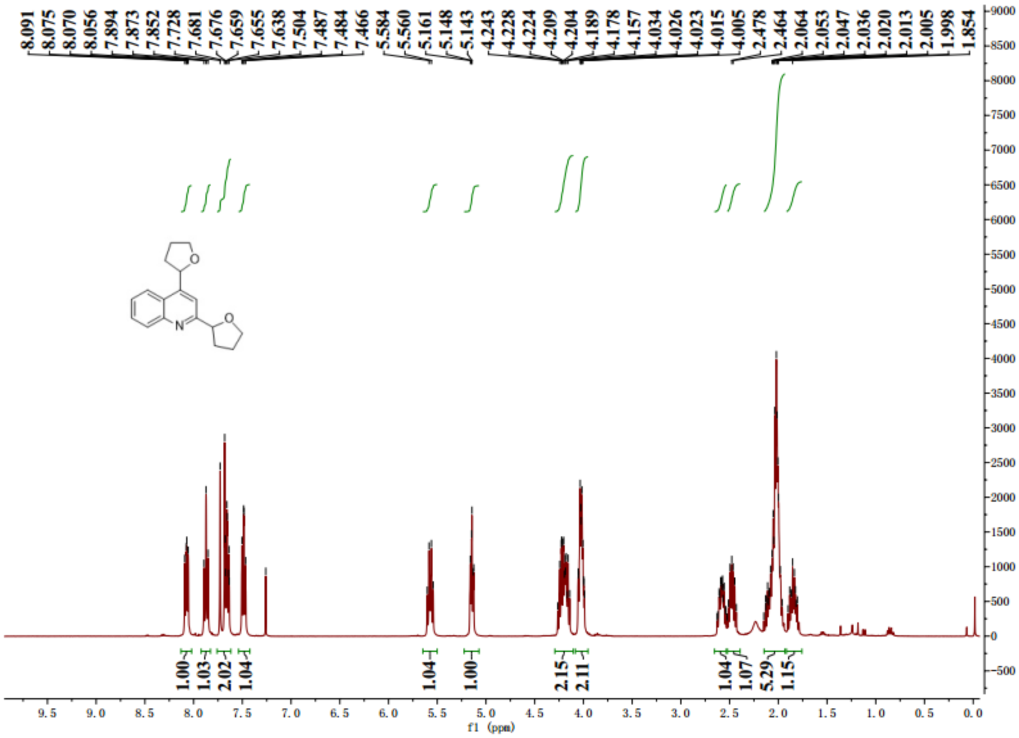

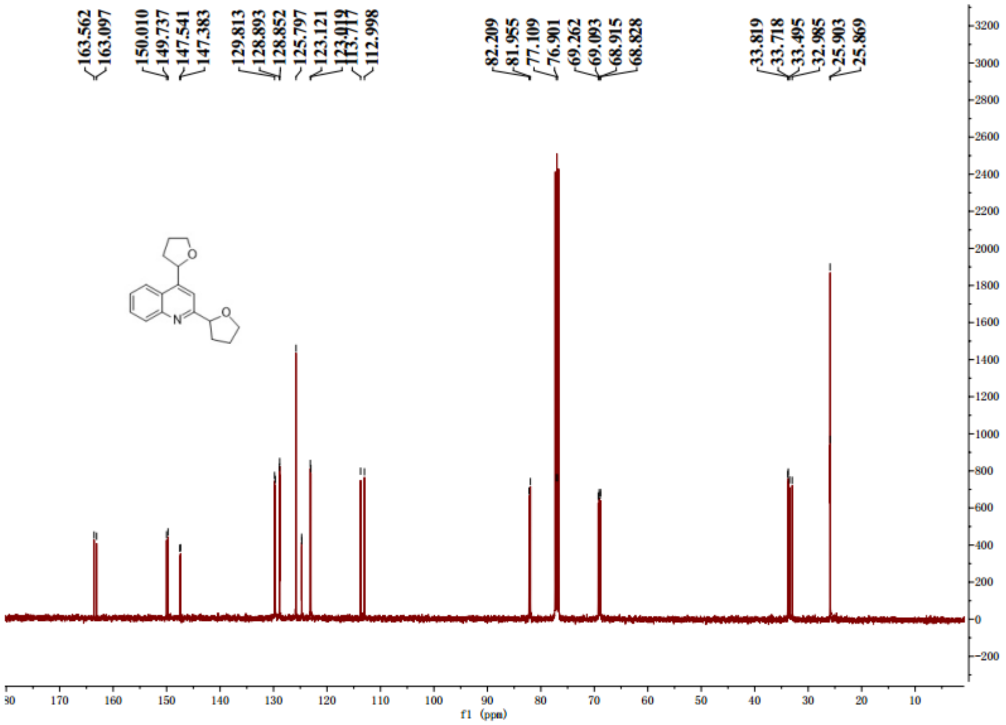

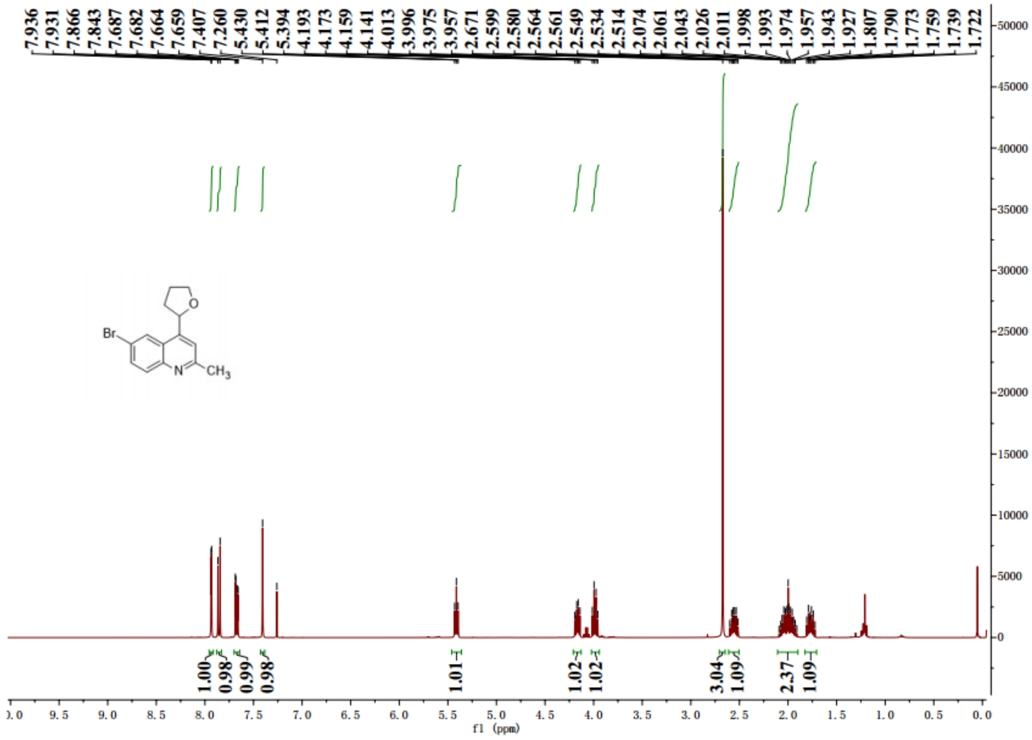

7.936
7.931
7.866
7.843
7.687
7.682
7.664
7.659
7.407
7.260
5.430
5.412
5.394
4.193
4.173
4.159
4.141
4.013
3.996
3.975
3.957
2.671
2.599
2.580
2.564
2.561
2.549
2.534
2.514
2.074
2.061
2.043
2.026
2.011
1.998
1.993
1.974
1.957
1.943
1.927
1.807
1.790
1.773
1.759
1.739
1.722
Br
O
N
CH3
1.00
0.98
0.99
0.98
1.01
1.02
1.02
3.04
1.09
2.37
1.09
f1 (ppm)

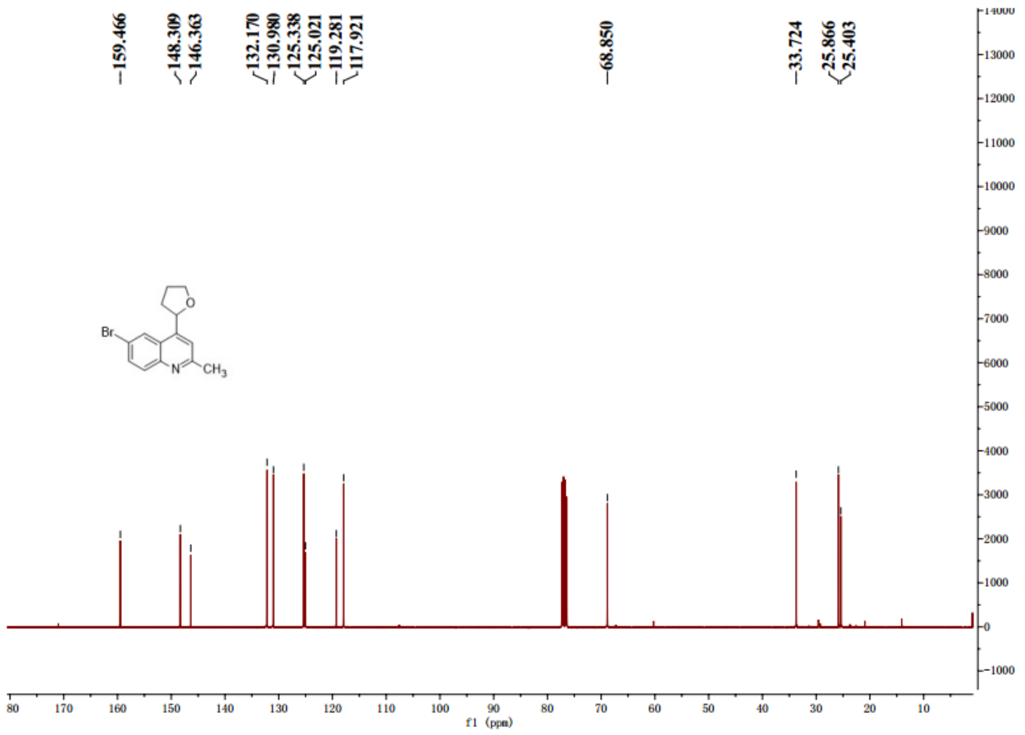

159.466
148.309
146.363
132.170
130.980
125.338
125.021
119.281
117.921
68.850
33.724
25.866
25.403
Br
O
N
CH3
f1 (ppm)

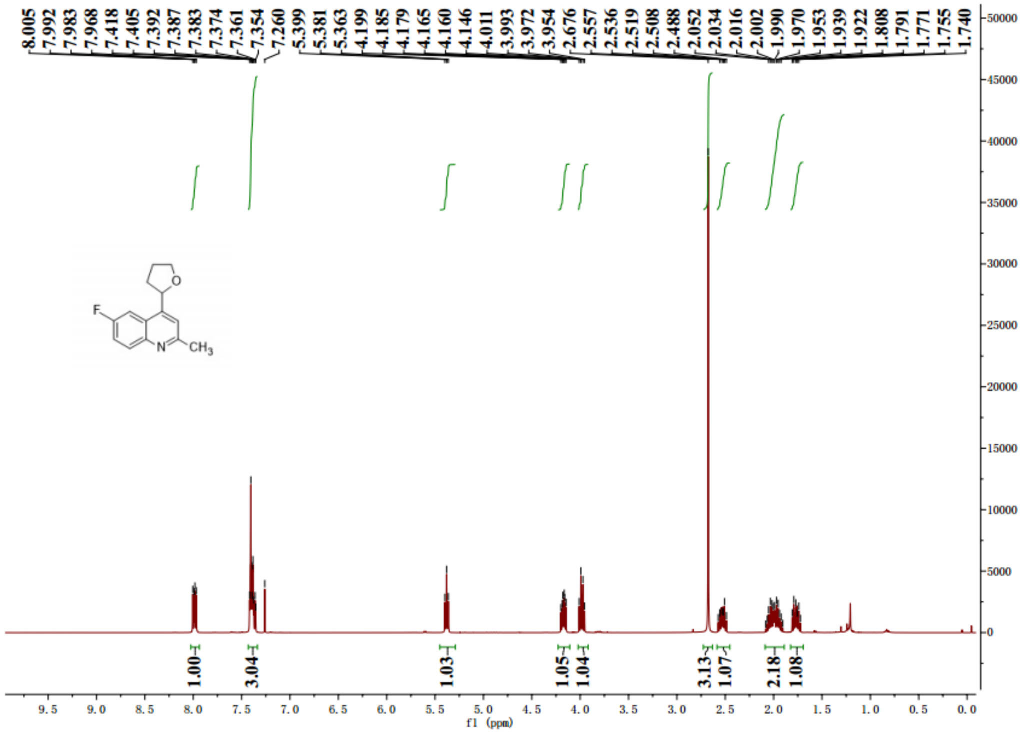

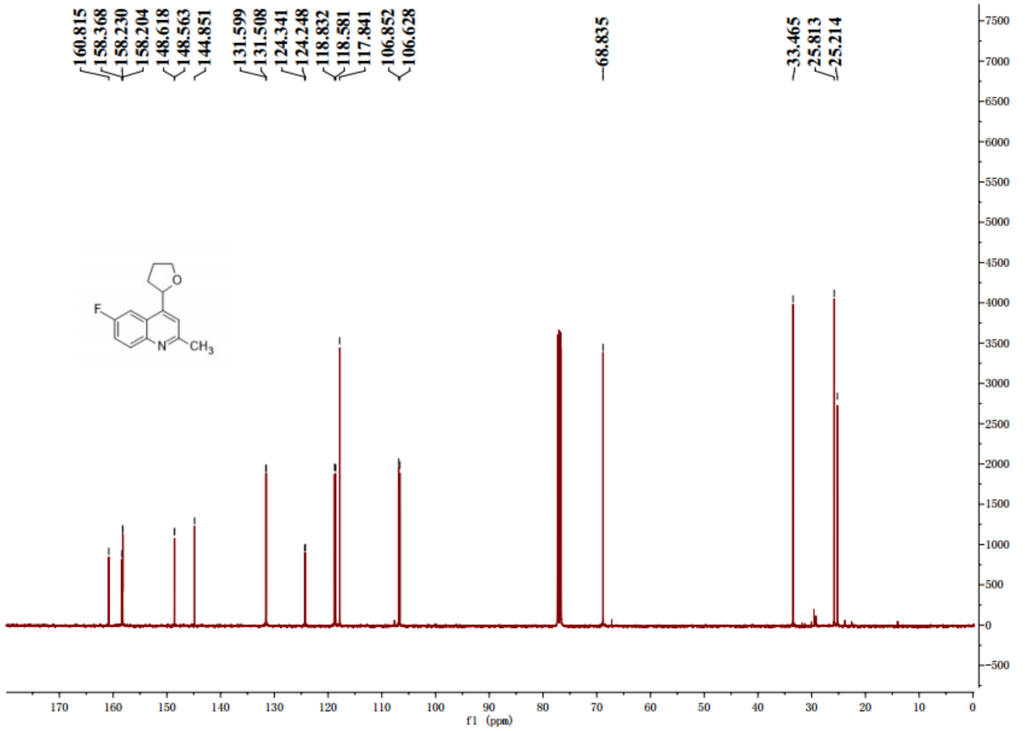

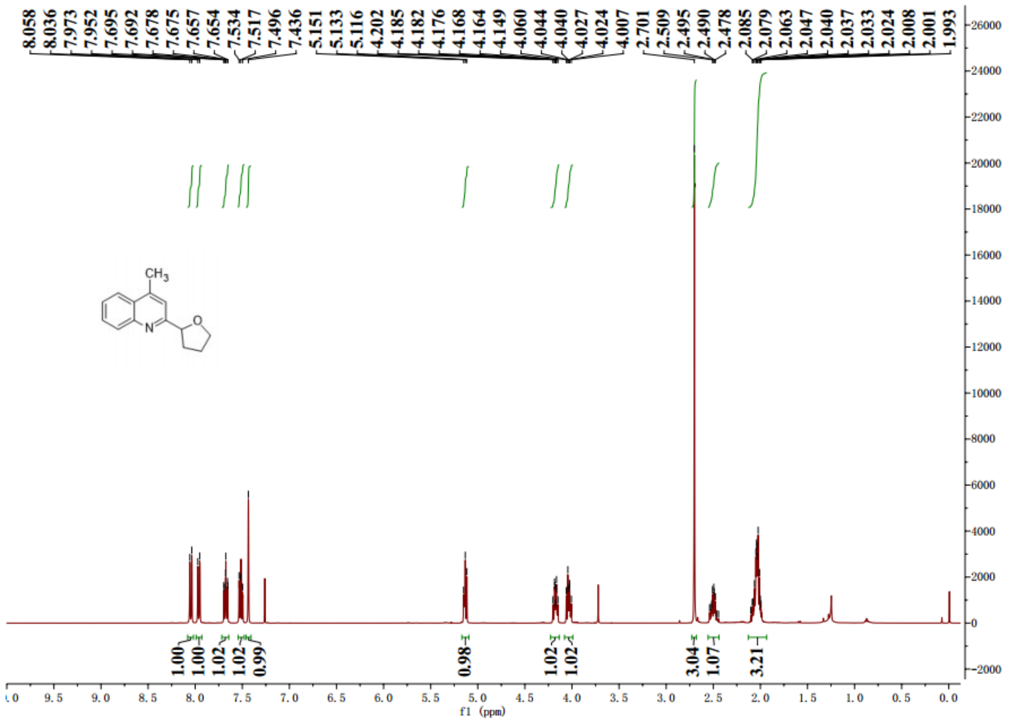
8.058
8.036
7.973
7.952
7.695
7.692
7.678
7.675
7.657
7.654
7.534
7.517
7.496
7.436
5.151
5.133
5.116
4.202
4.185
4.182
4.176
4.168
4.164
4.149
4.060
4.044
4.040
4.027
4.024
4.007
2.701
2.509
2.495
2.490
2.478
2.085
2.079
2.063
2.047
2.040
2.037
2.033
2.024
2.008
2.001
1.993
CH3
N
O
1.00
1.00
1.02
1.02
0.99
0.98
1.02
1.02
3.04
1.07
3.21
f1 (ppm)

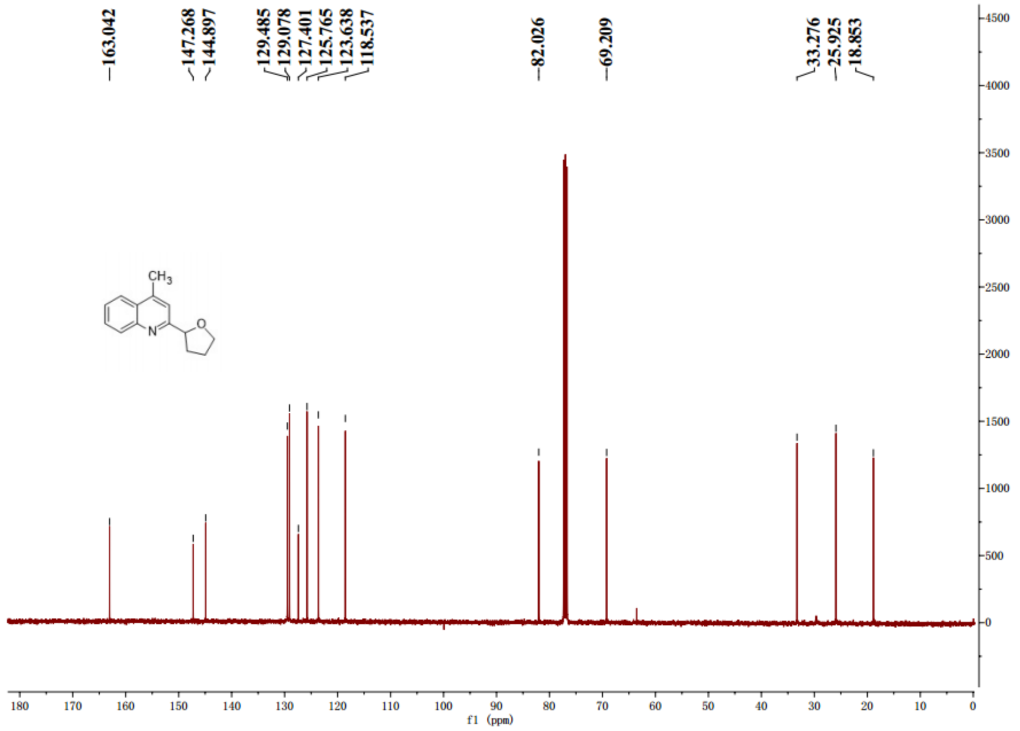
163.042
147.268
144.897
129.485
129.078
127.401
125.765
123.638
118.537
82.026
69.209
33.276
25.925
18.853
CH3
N
O
f1 (ppm)

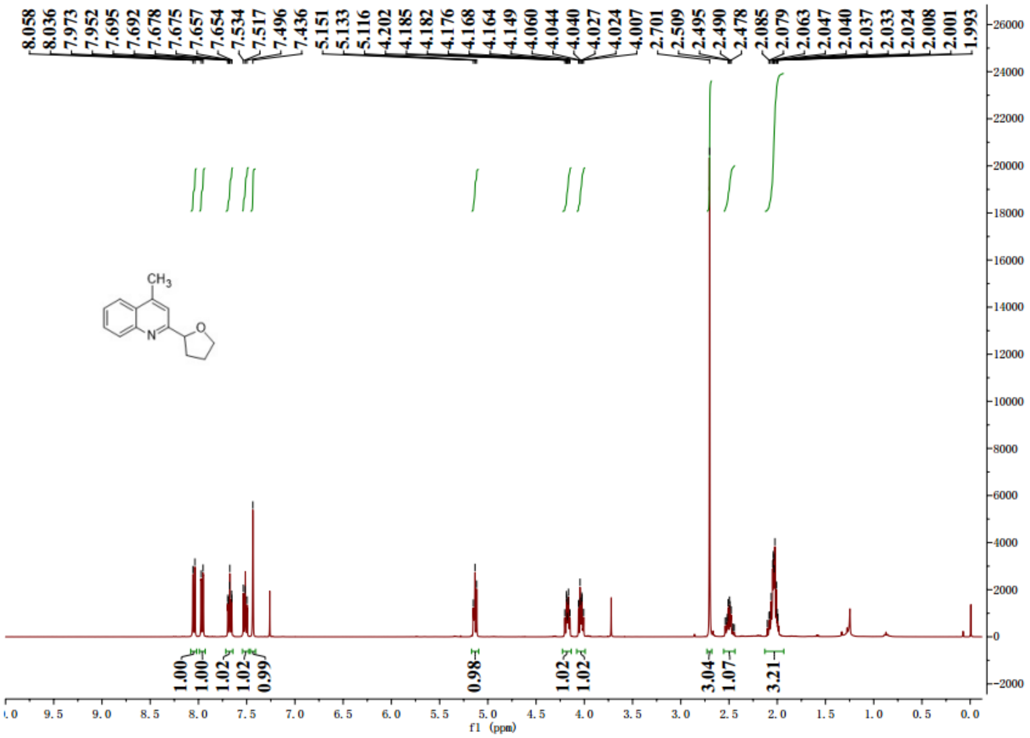
8.058
8.036
7.973
7.952
7.695
7.692
7.678
7.675
7.657
7.654
7.534
7.517
7.496
7.436
5.151
5.133
5.116
4.202
4.185
4.182
4.176
4.168
4.164
4.149
4.060
4.044
4.040
4.027
4.024
4.007
2.701
2.509
2.495
2.490
2.478
2.085
2.079
2.063
2.047
2.040
2.037
2.033
2.024
2.008
2.001
1.993
CH3
N
O
1.00
1.00
1.02
1.02
0.99
0.98
1.02
1.02
3.04
1.07
3.21
f1 (ppm)

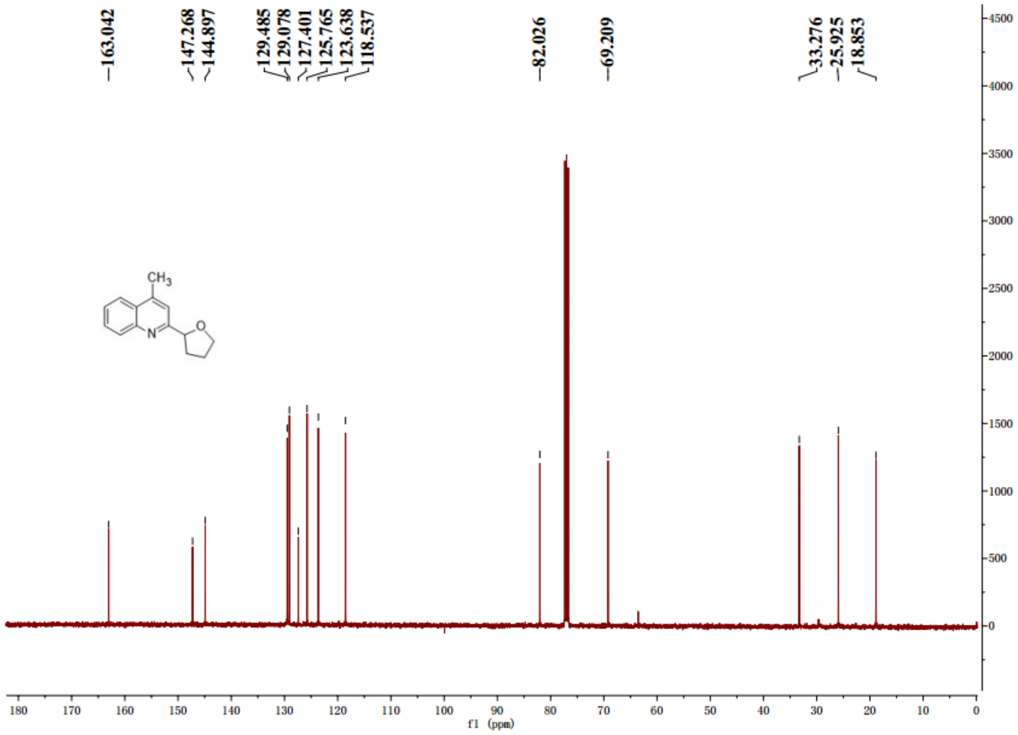
163.042
147.268
144.897
129.485
129.078
127.401
125.765
123.638
118.537
82.026
69.209
33.276
25.925
18.853
CH3
N
O
f1 (ppm)

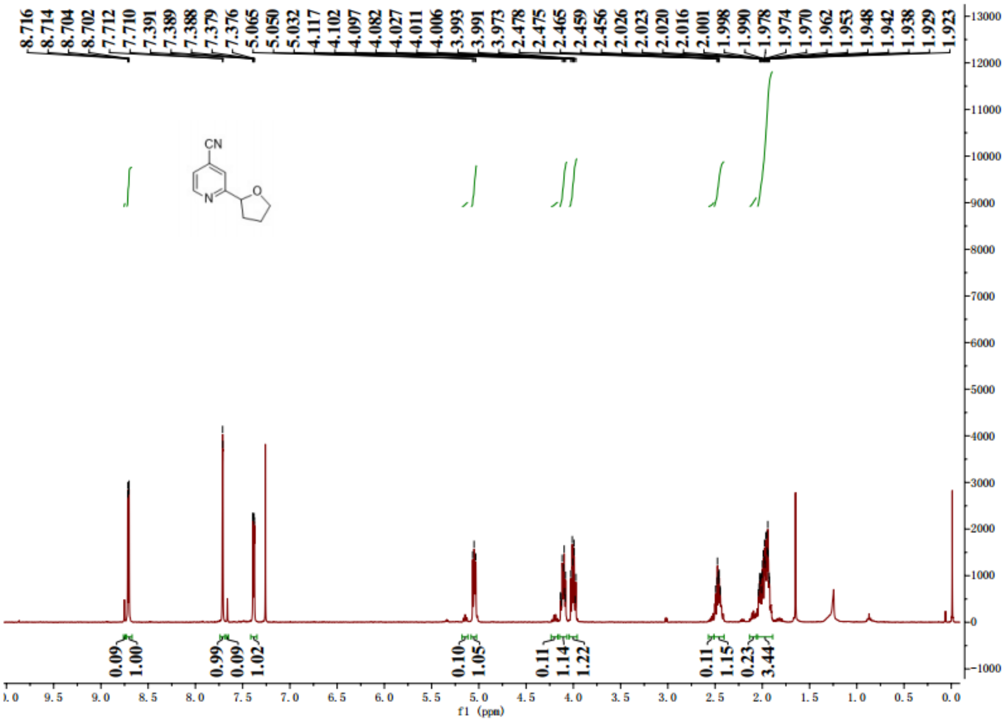

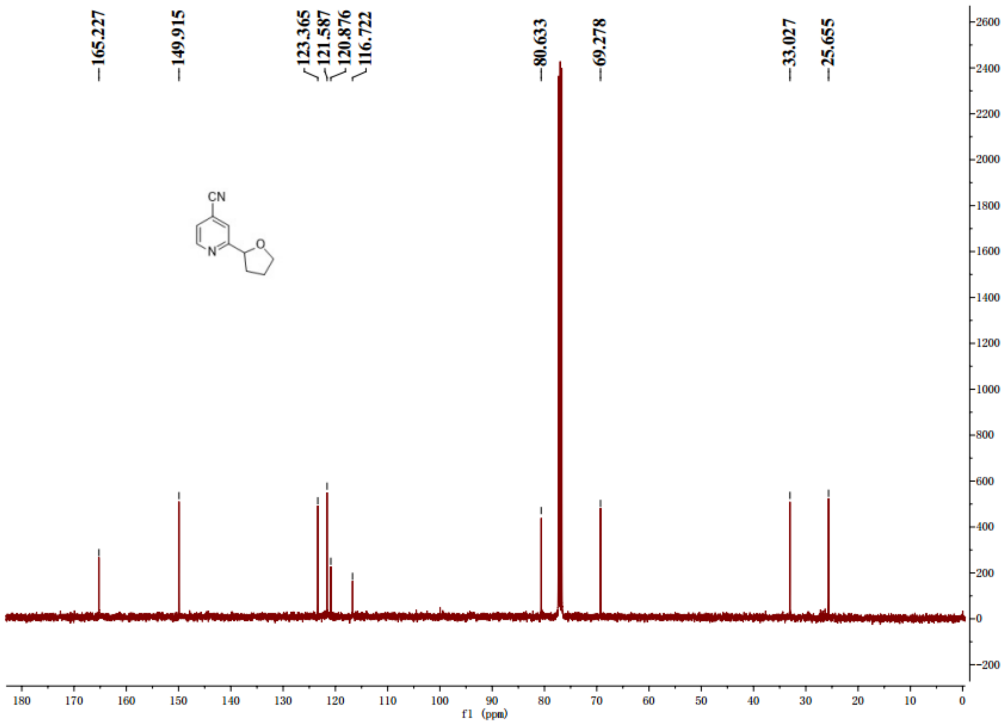

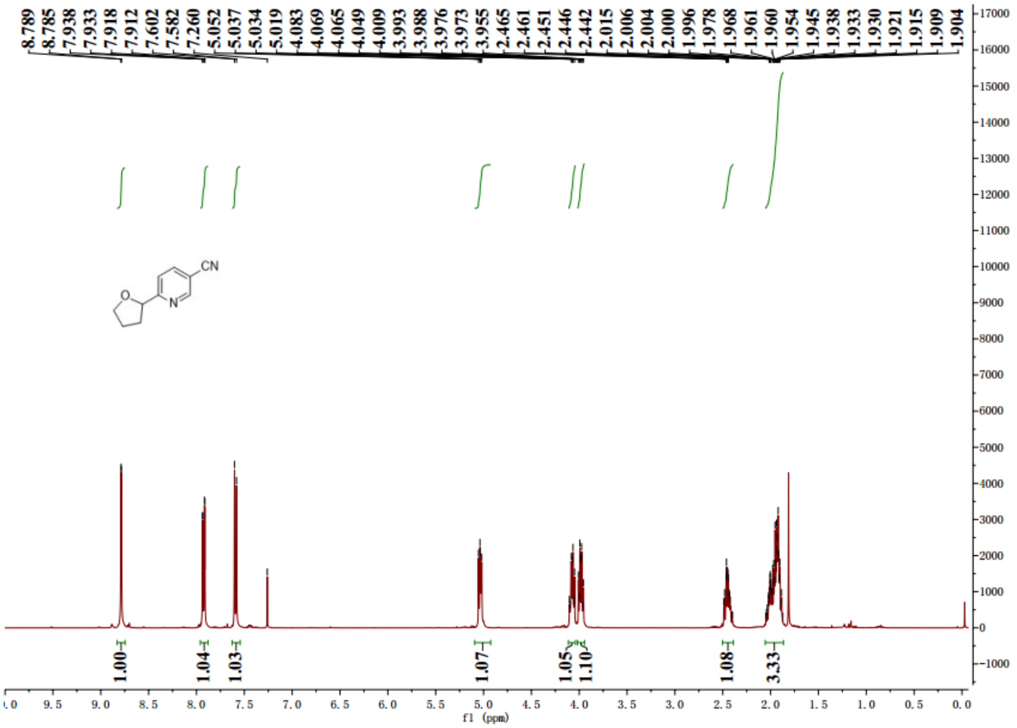

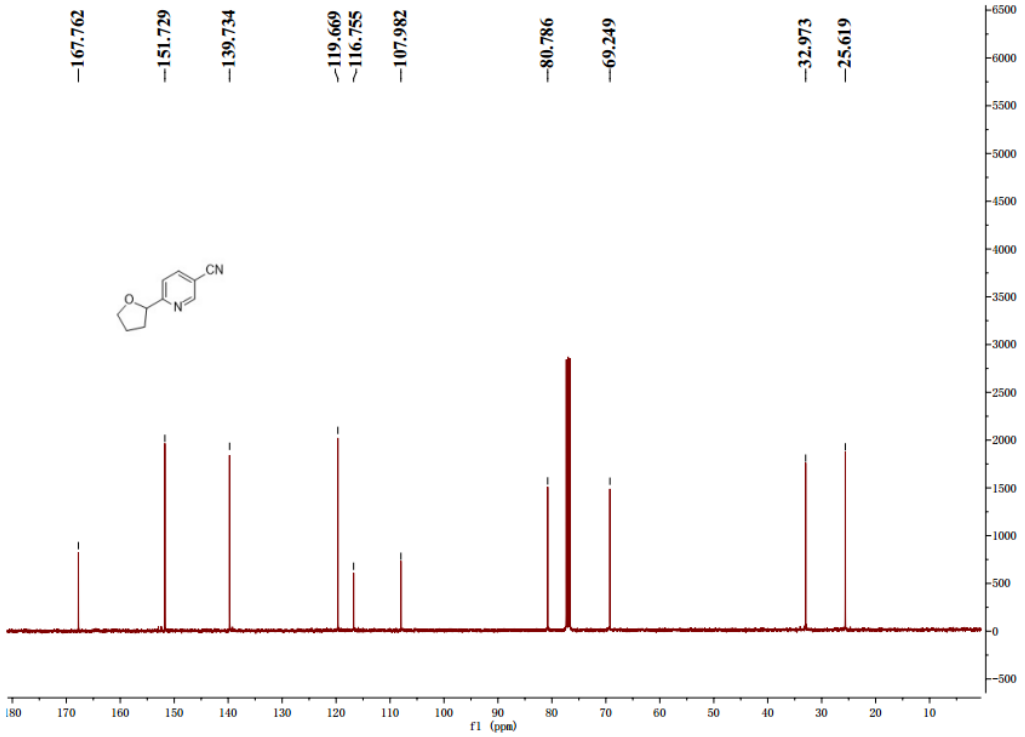

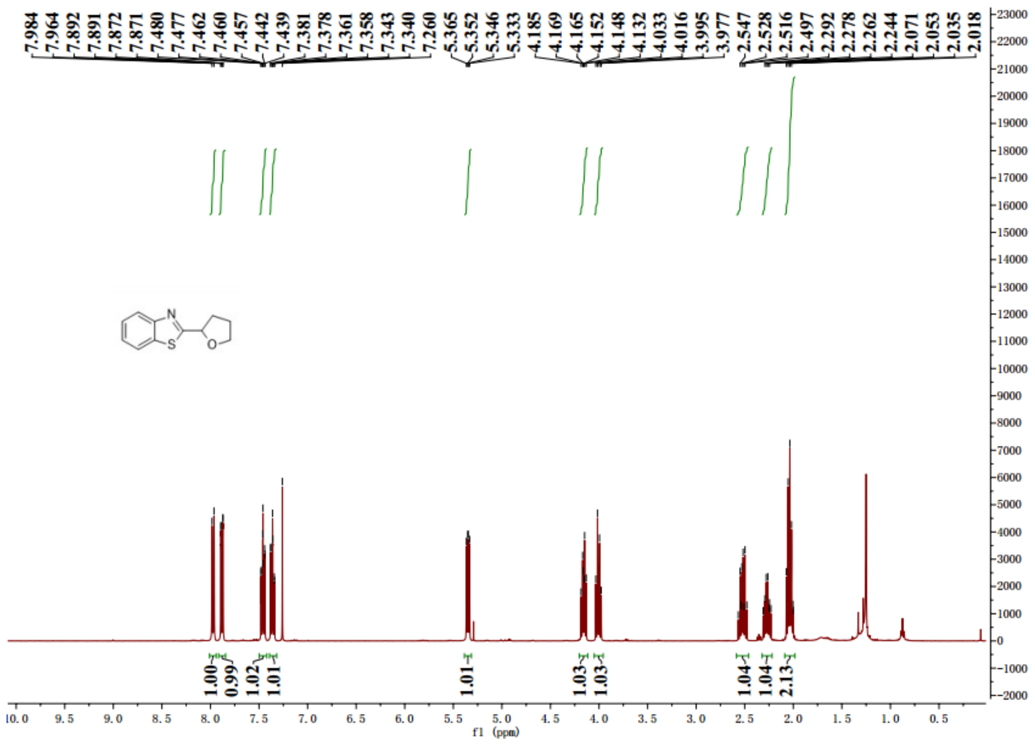

7.984
7.964
7.892
7.891
7.872
7.871
7.480
7.477
7.462
7.460
7.457
7.442
7.439
7.381
7.378
7.361
7.358
7.343
7.340
7.260
5.365
5.352
5.346
5.333
4.185
4.169
4.165
4.152
4.148
4.132
4.033
4.016
3.995
3.977
2.547
2.528
2.516
2.497
2.292
2.278
2.262
2.244
2.071
2.053
2.035
2.018
N
S
O
1.00
0.99
1.02
1.01
1.01
1.03
1.03
1.04
1.04
2.13
f1 (ppm)

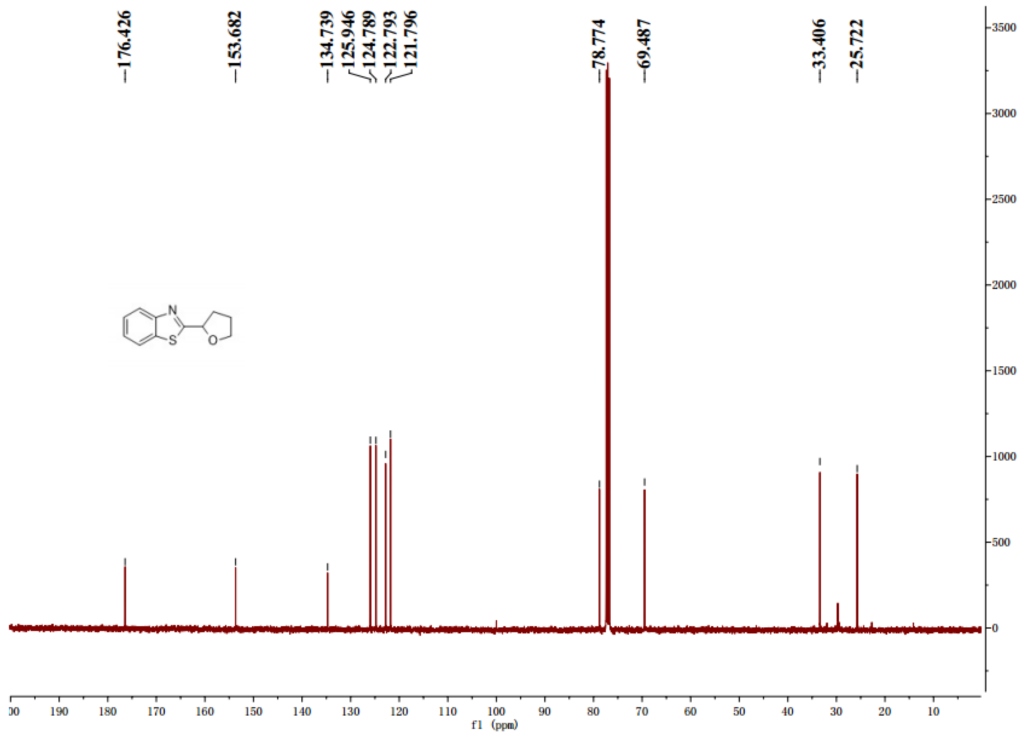

176.426
153.682
134.739
125.946
124.789
122.793
121.796
78.774
69.487
33.406
25.722
N
S
O
f1 (ppm)

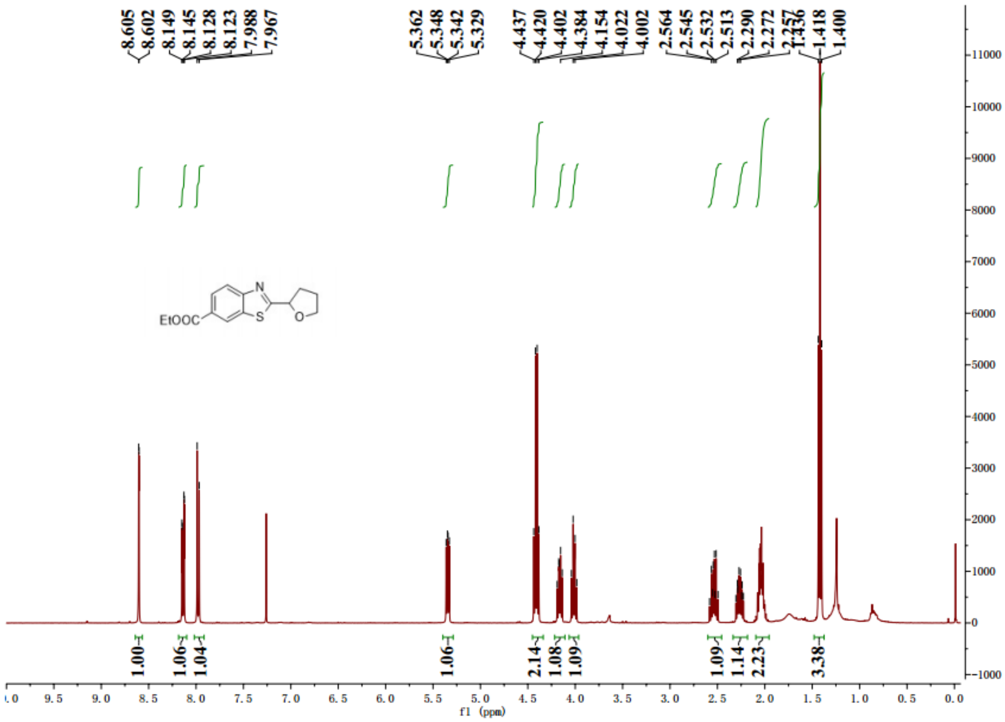
8.605
8.602
8.149
8.145
8.128
8.123
7.988
7.967
5.362
5.348
5.342
5.329
4.437
4.420
4.402
4.384
4.154
4.022
4.002
2.564
2.545
2.532
2.513
2.290
2.272
2.257
1.436
1.418
1.400
EtOOC
N
S
O
1.00
1.06
1.04
1.06
2.14
1.08
1.09
1.09
1.14
2.23
3.38
f1 (ppm)

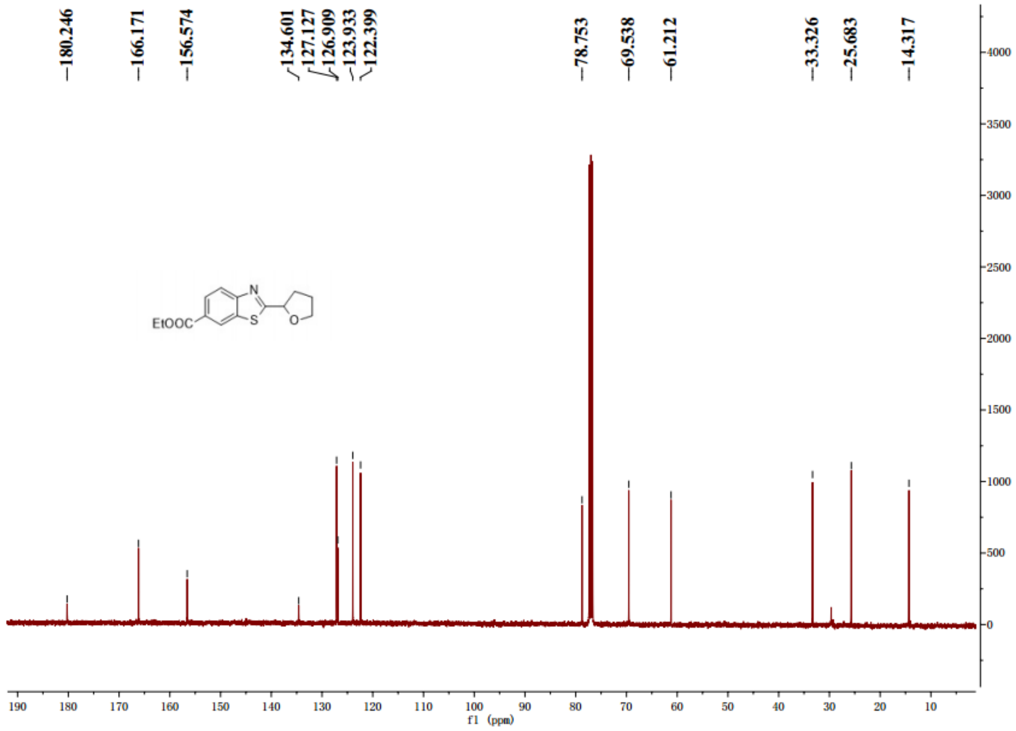
180.246
166.171
156.574
134.601
127.127
126.909
123.933
122.399
78.753
69.538
61.212
33.326
25.683
14.317
EtOOC
N
S
O
f1 (ppm)

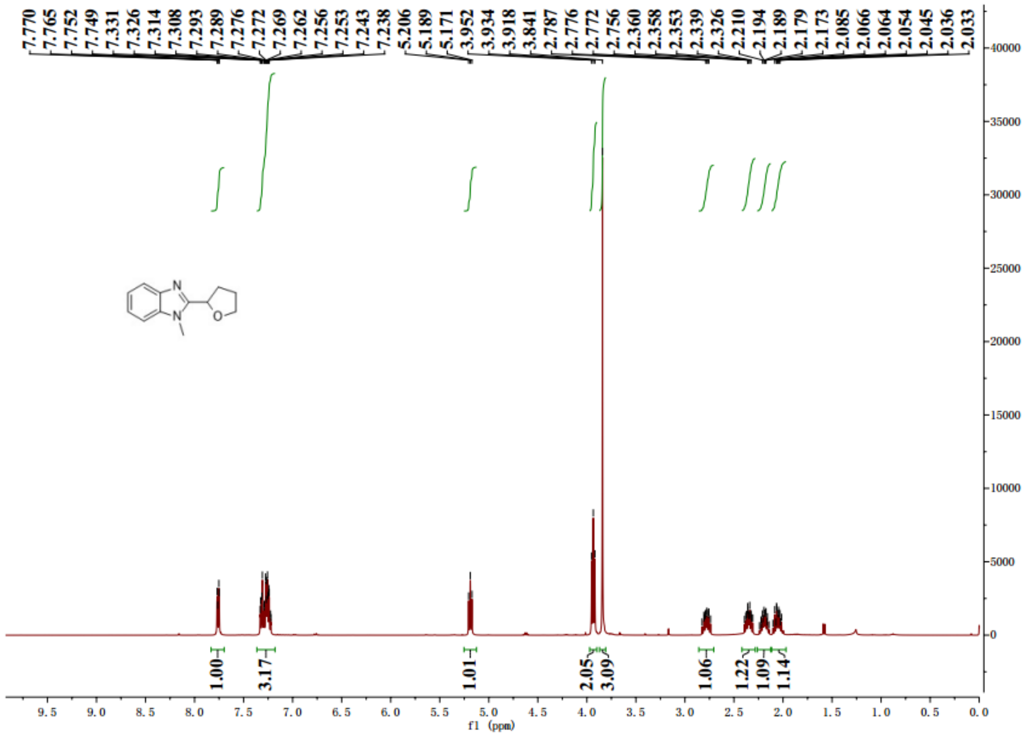

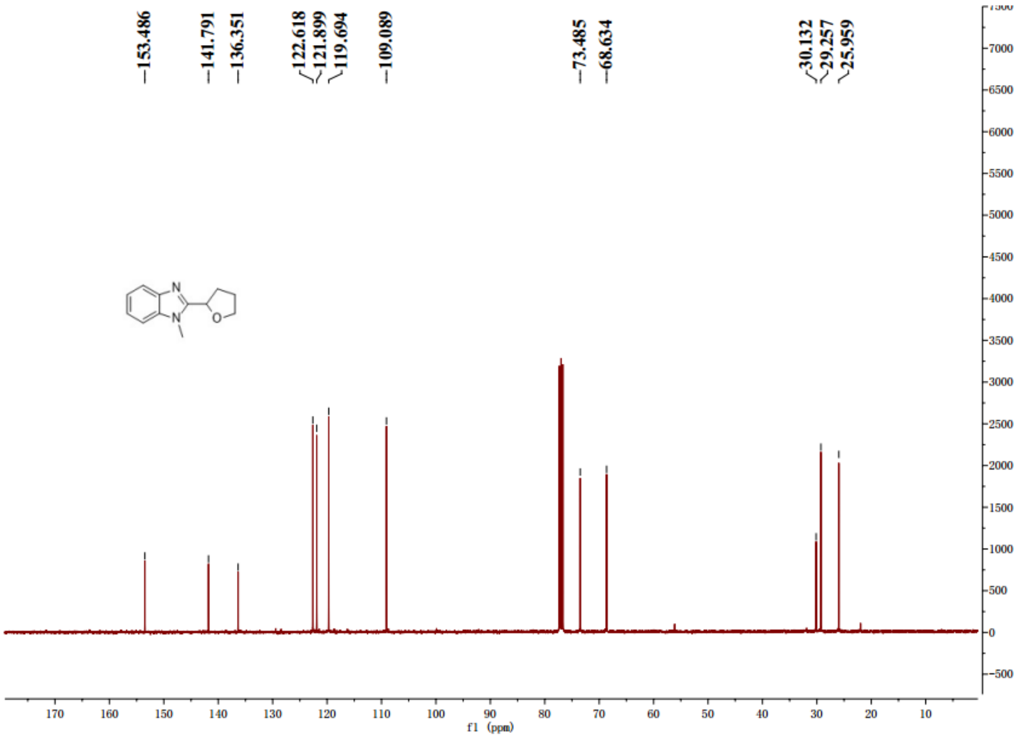

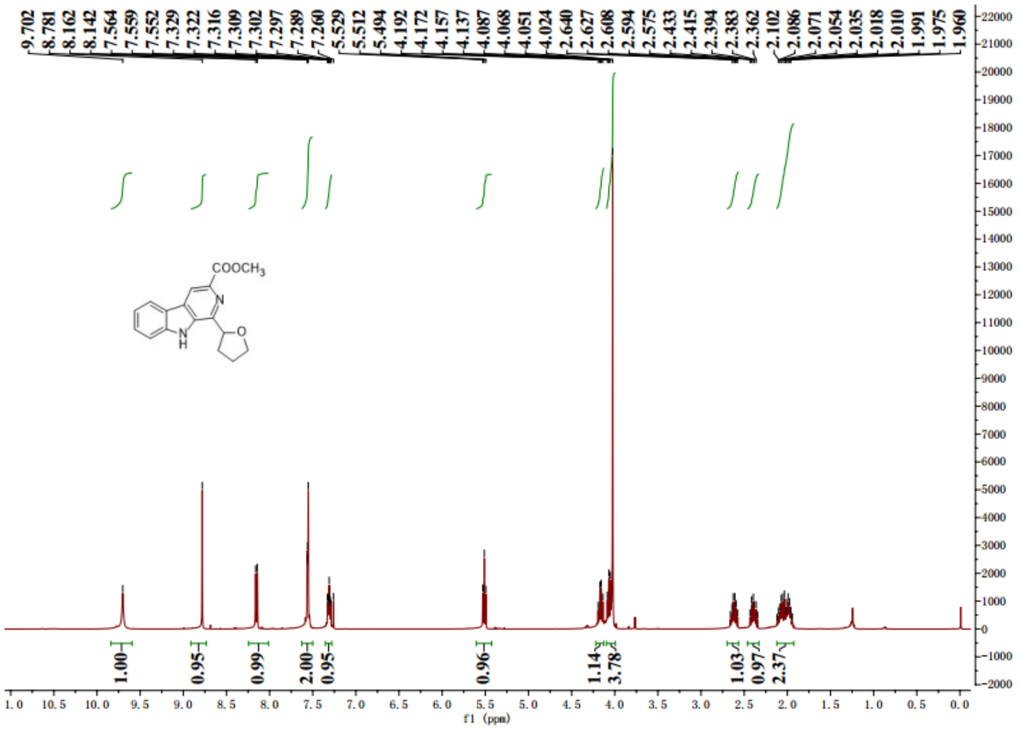
9.702
8.781
8.162
8.142
7.564
7.559
7.552
7.329
7.322
7.316
7.309
7.302
7.297
7.289
7.260
5.529
5.512
5.494
4.192
4.172
4.157
4.137
4.087
4.068
4.051
4.024
2.640
2.627
2.608
2.594
2.575
2.433
2.415
2.394
2.383
2.362
2.102
2.086
2.071
2.054
2.035
2.018
2.010
1.991
1.975
1.960
COOCH3
N
H
O
1.00
0.95
0.99
2.00
0.95
0.96
1.14
3.78
1.03
0.97
2.37
f1 (ppm)

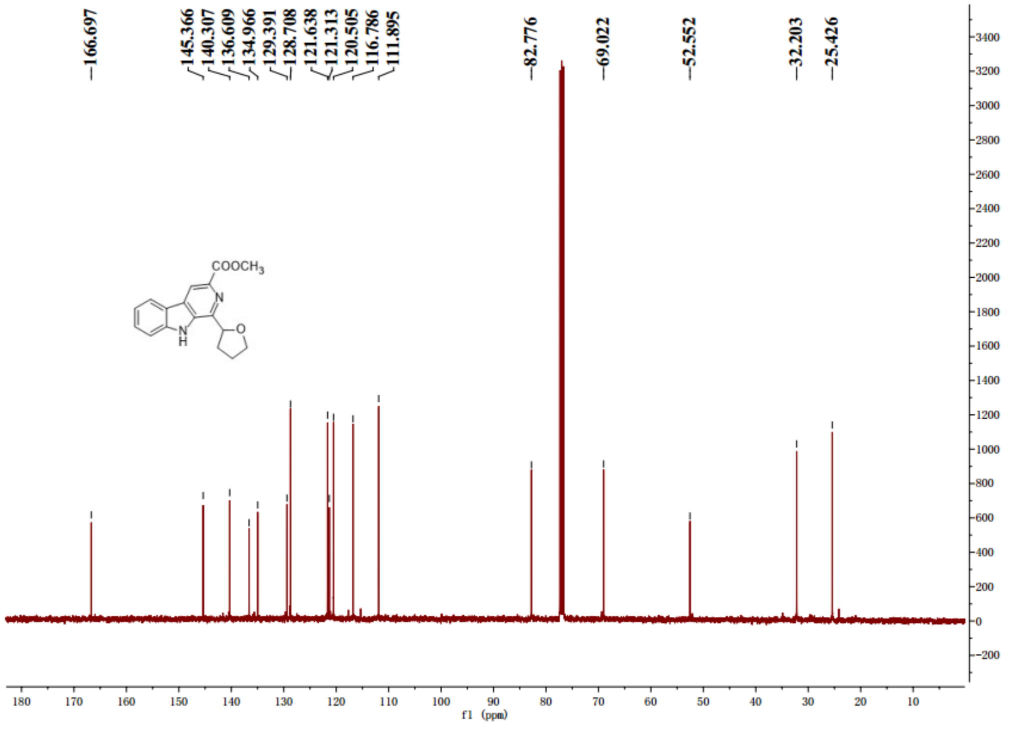
166.697
145.366
140.307
136.609
134.966
129.391
128.708
121.638
121.313
120.505
116.786
111.895
82.776
69.022
52.552
32.203
25.426
COOCH3
N
H
O
f1 (ppm)

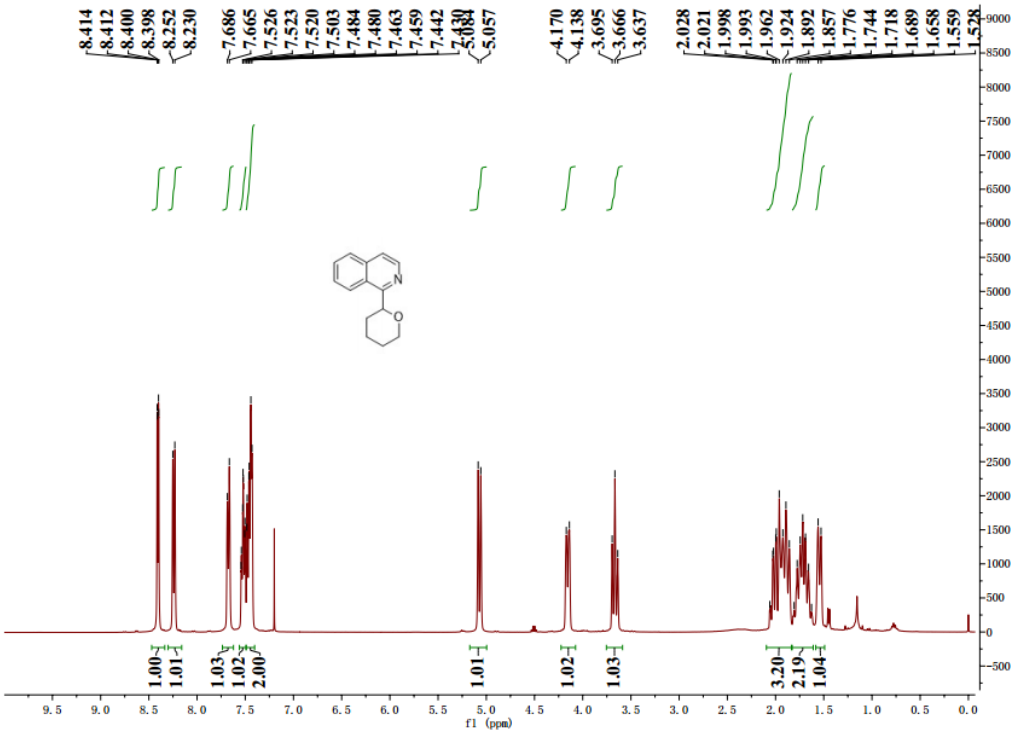

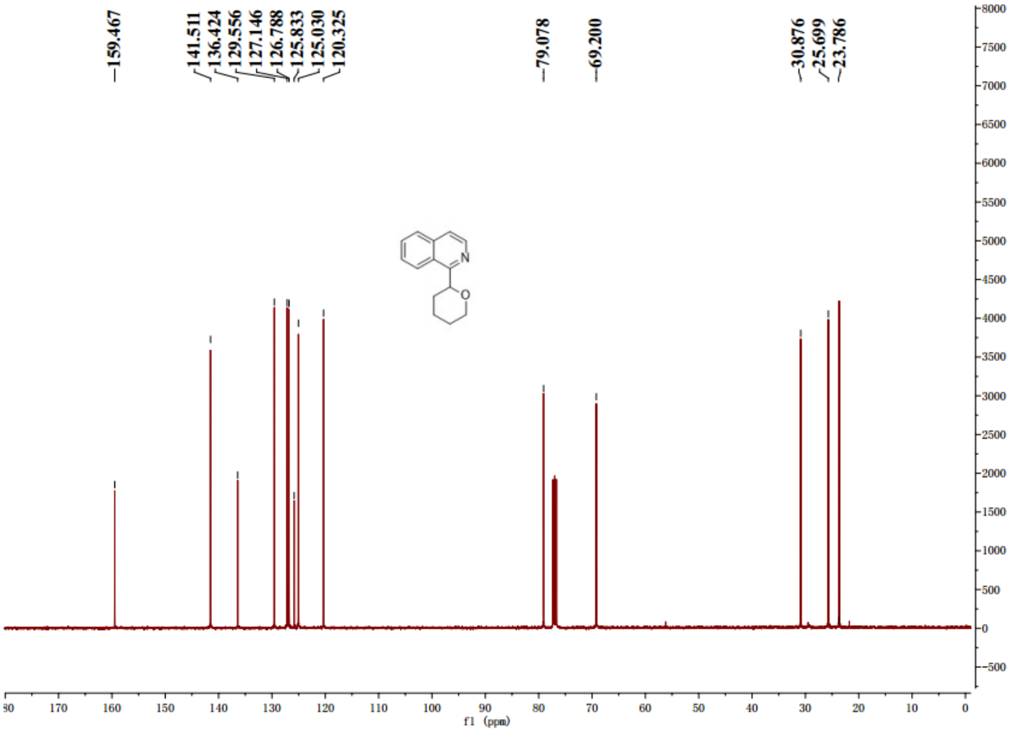

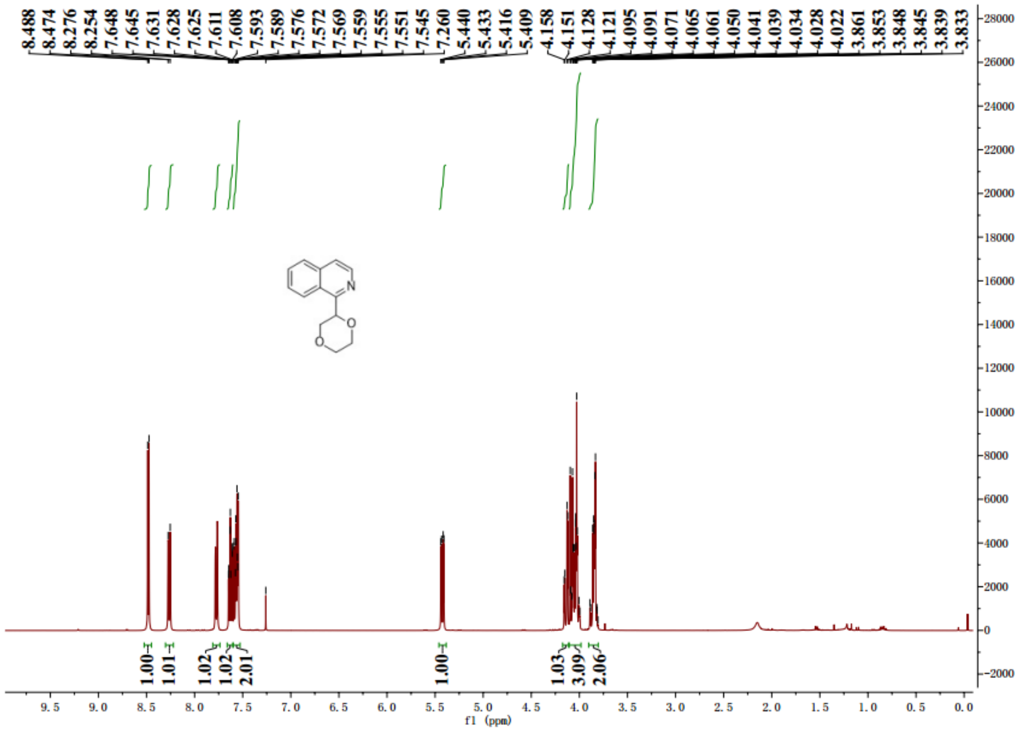

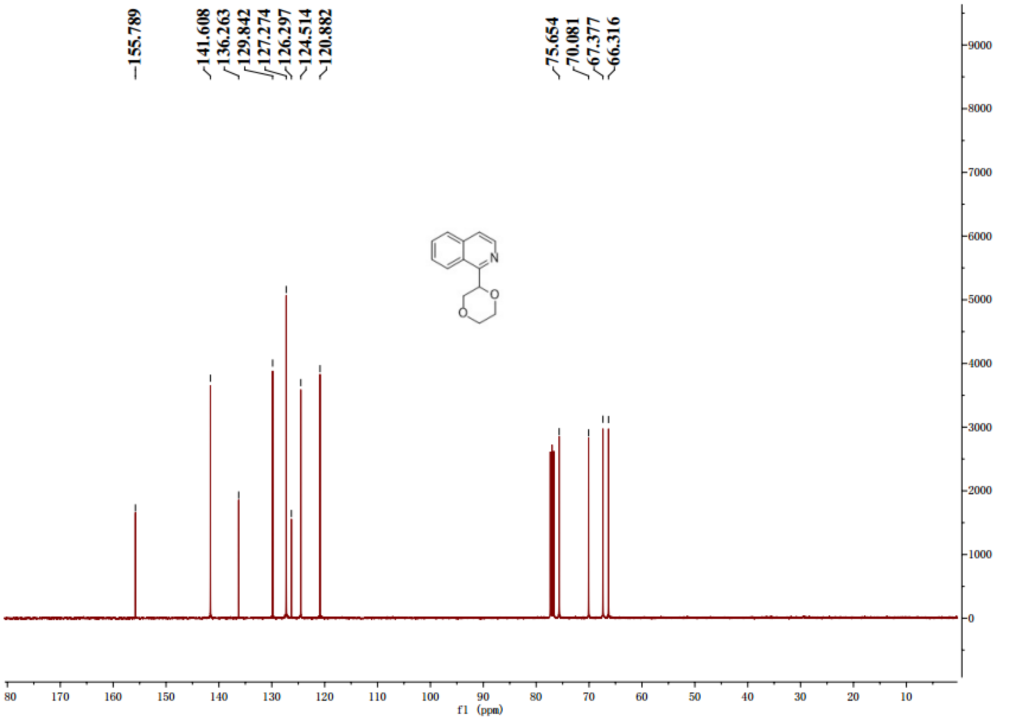

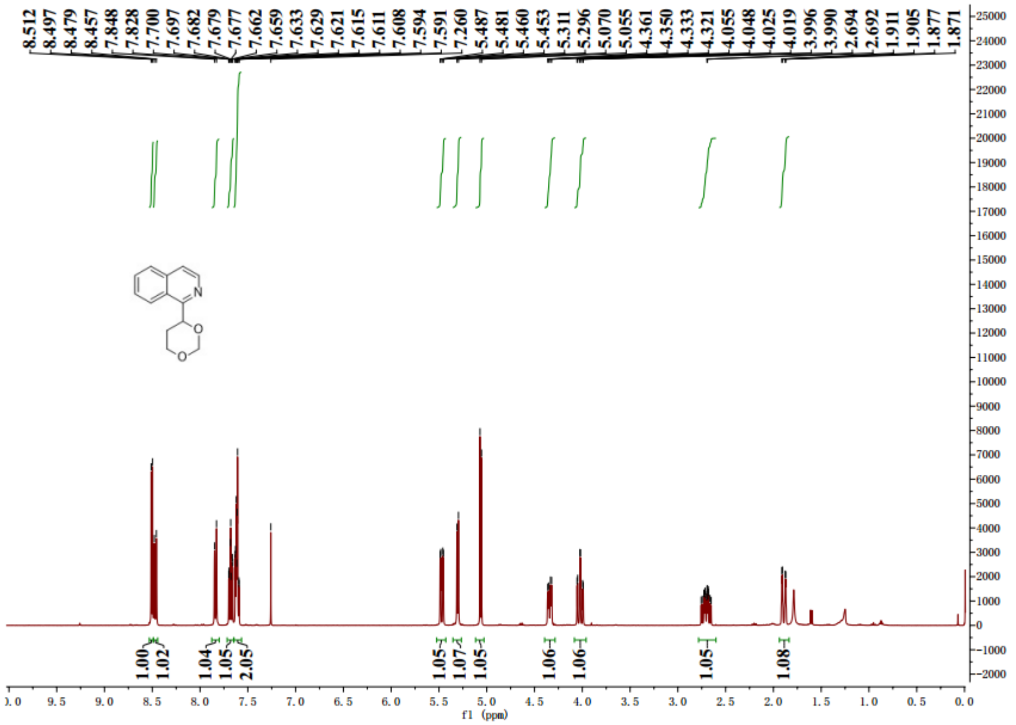

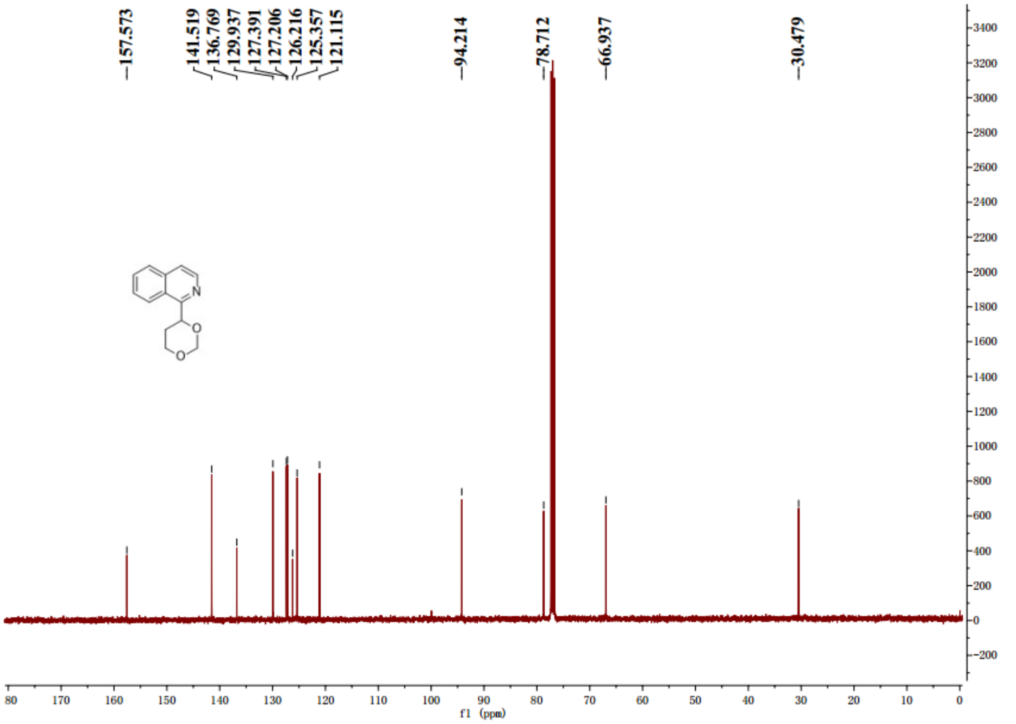

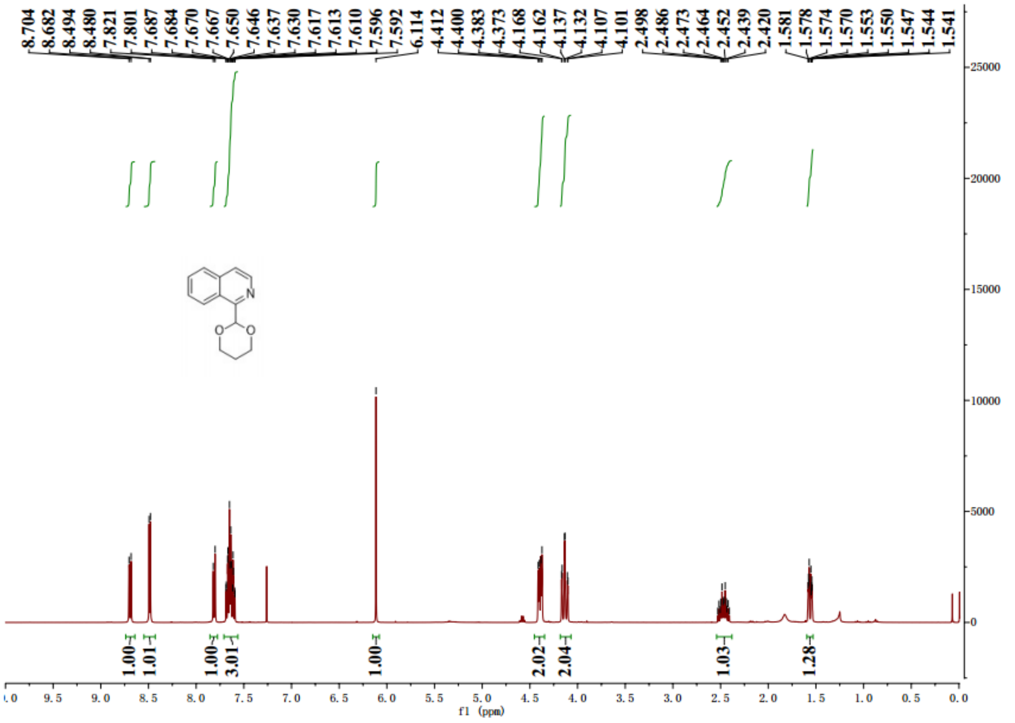
8.704
8.682
8.494
8.480
7.821
7.801
7.687
7.684
7.670
7.667
7.650
7.646
7.637
7.630
7.617
7.613
7.610
7.596
7.592
6.114
4.412
4.400
4.383
4.373
4.168
4.162
4.137
4.132
4.107
4.101
2.498
2.486
2.473
2.464
2.452
2.439
2.420
1.581
1.578
1.574
1.570
1.553
1.550
1.547
1.544
1.541
1.00
1.01
1.00
3.01
1.00
2.02
2.04
1.03
1.28
f1 (ppm)

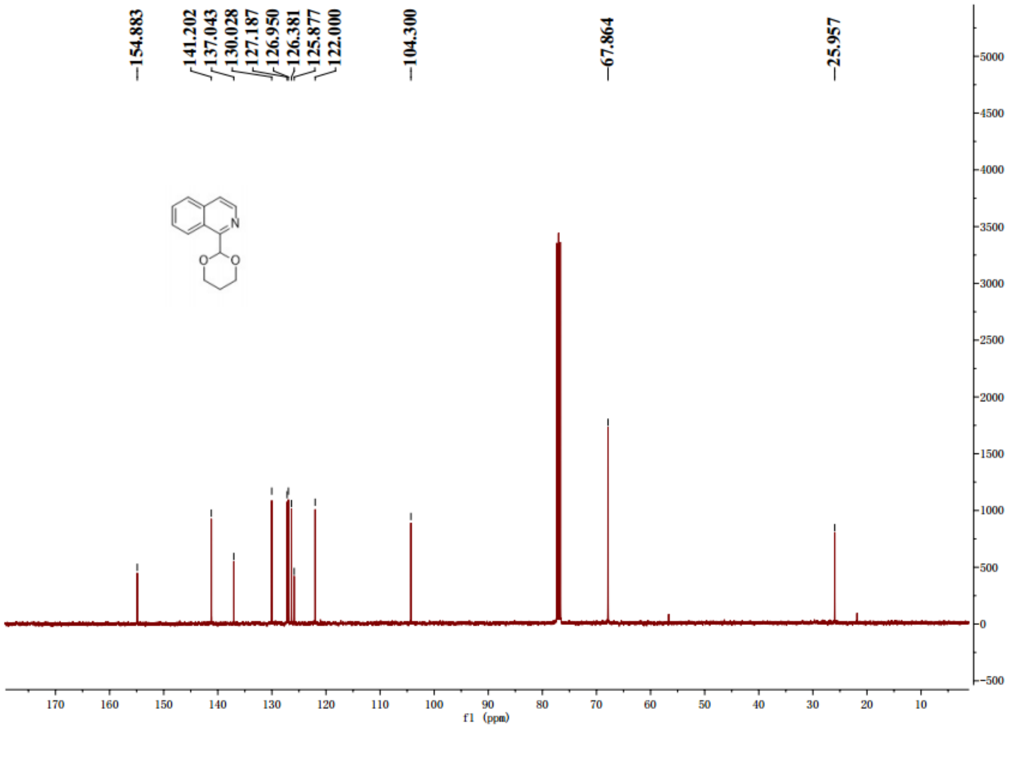
154.883
141.202
137.043
130.028
127.187
126.950
126.381
125.877
122.000
104.300
67.864
25.957
f1 (ppm)

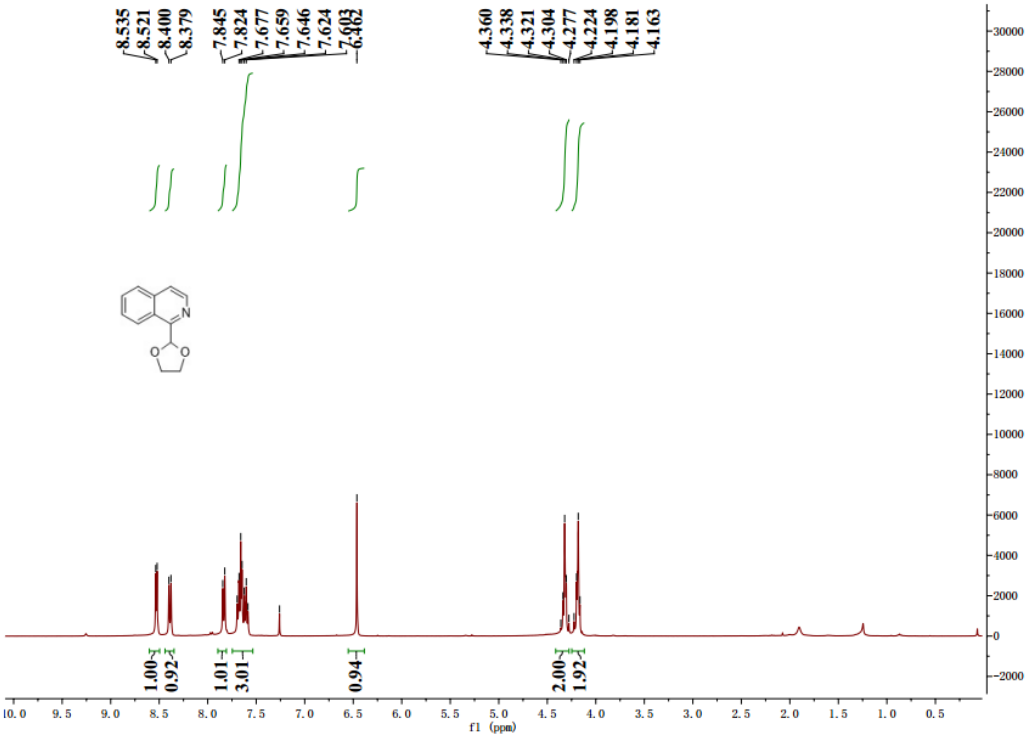
8.535
8.521
8.400
8.379
7.845
7.824
7.677
7.659
7.646
7.624
7.603
6.462
4.360
4.338
4.321
4.304
4.277
4.224
4.198
4.181
4.163
1.00
0.92
1.01
3.01
0.94
2.00
1.92
f1 (ppm)

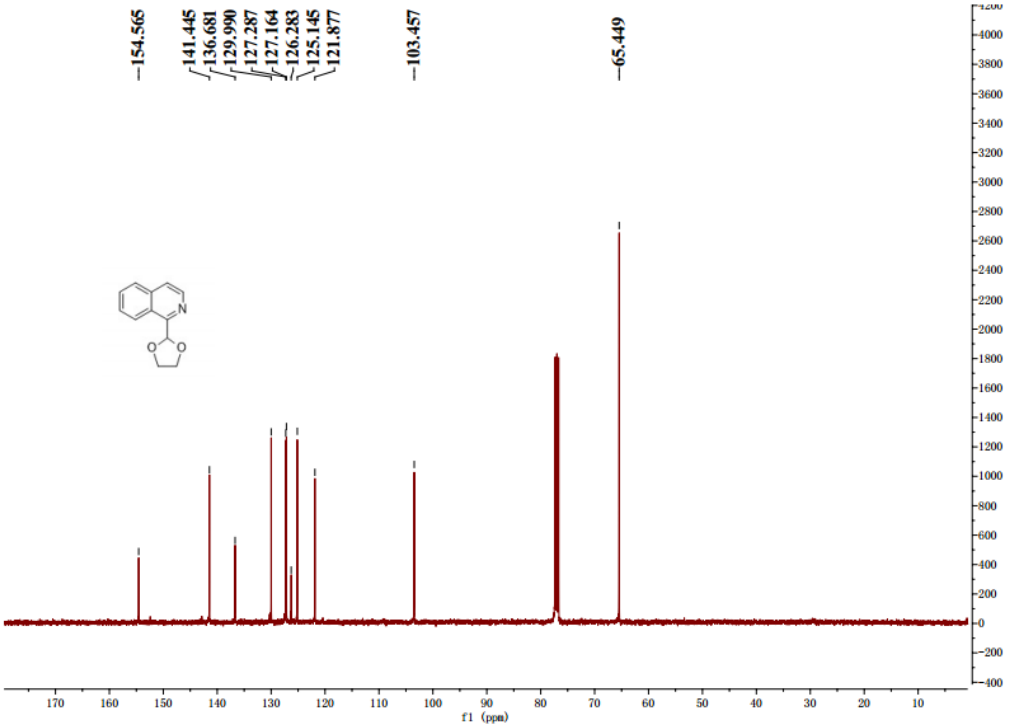
154.565
141.445
136.681
129.990
127.287
127.164
126.283
125.145
121.877
103.457
65.449
f1 (ppm)

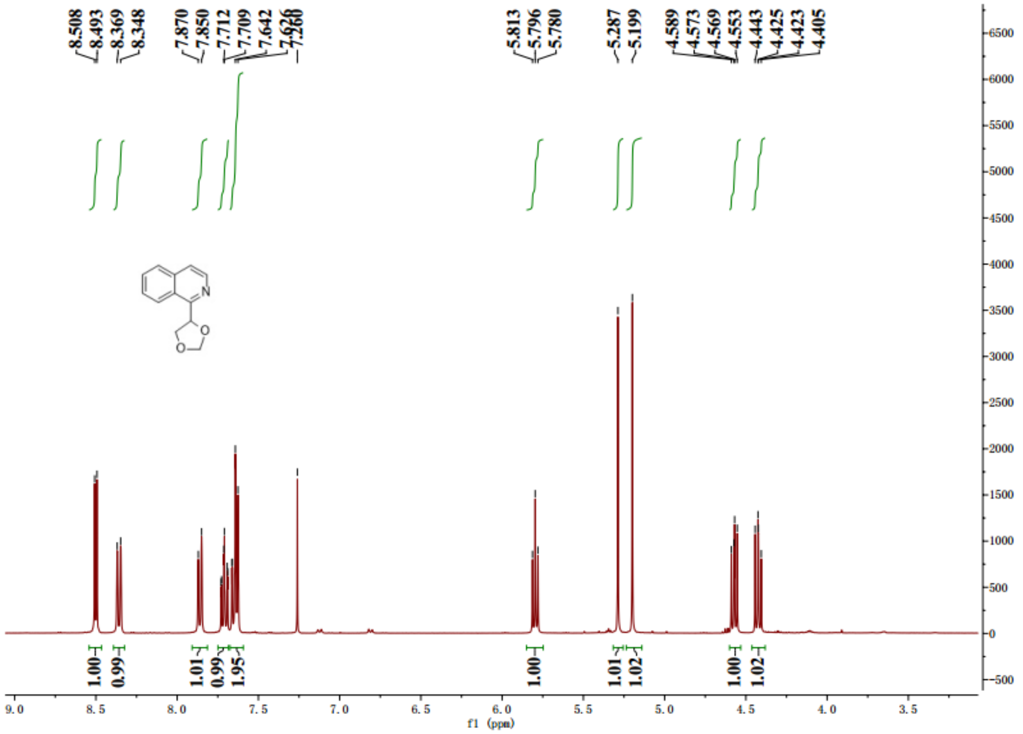
8.508
8.493
8.369
8.348
7.870
7.850
7.712
7.709
7.642
7.626
7.260
5.813
5.796
5.780
5.287
5.199
4.589
4.573
4.569
4.553
4.443
4.425
4.423
4.405
1.00
0.99
1.01
0.99
1.95
1.00
1.01
1.02
1.00
1.02
f1 (ppm)

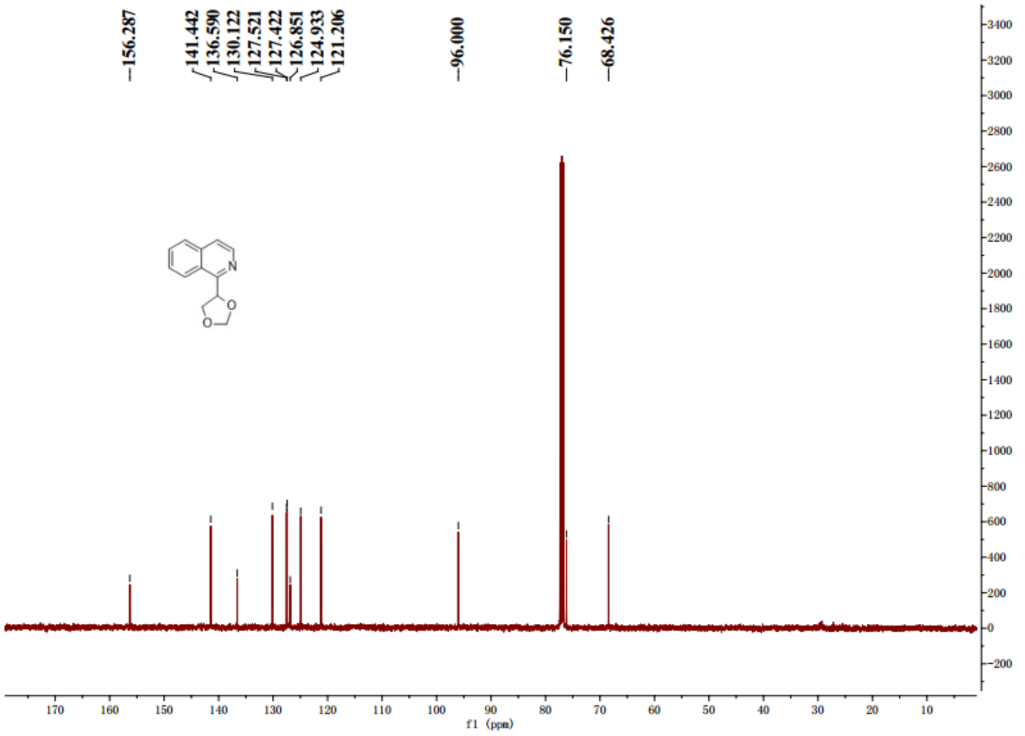
156.287
141.442
136.590
130.122
127.521
127.422
126.851
124.933
121.206
96.000
76.150
68.426
f1 (ppm)

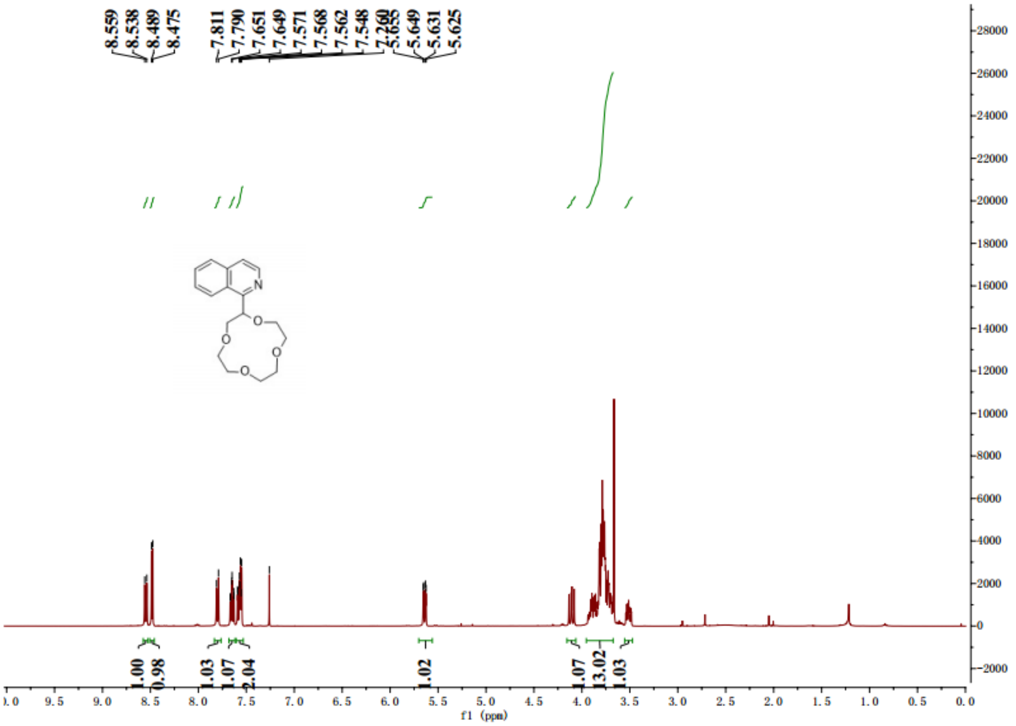

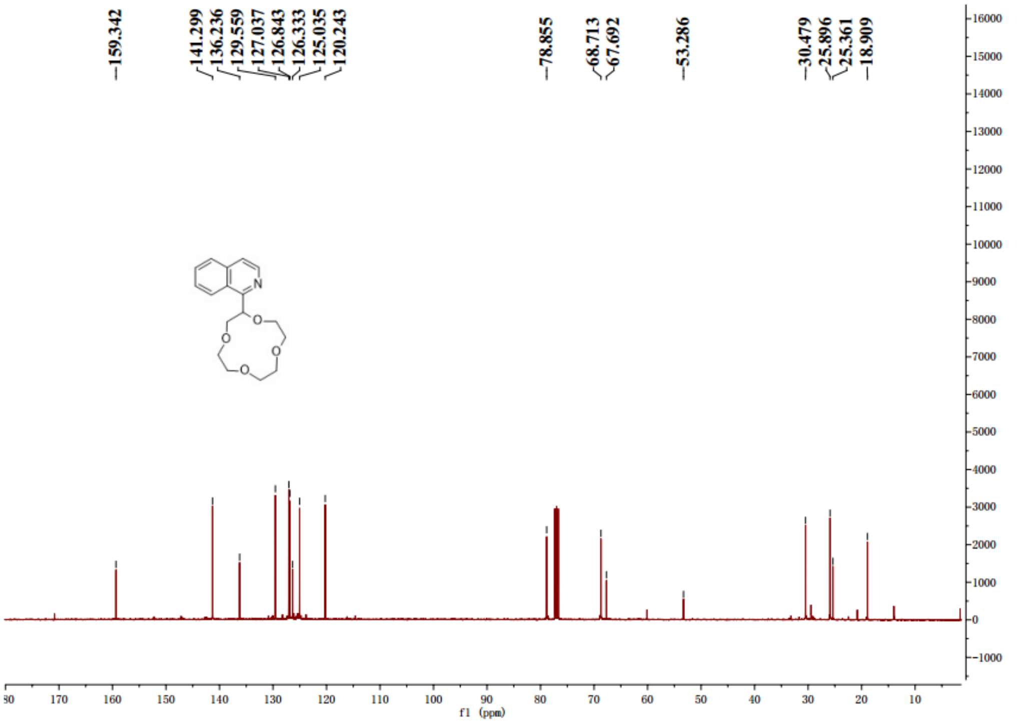

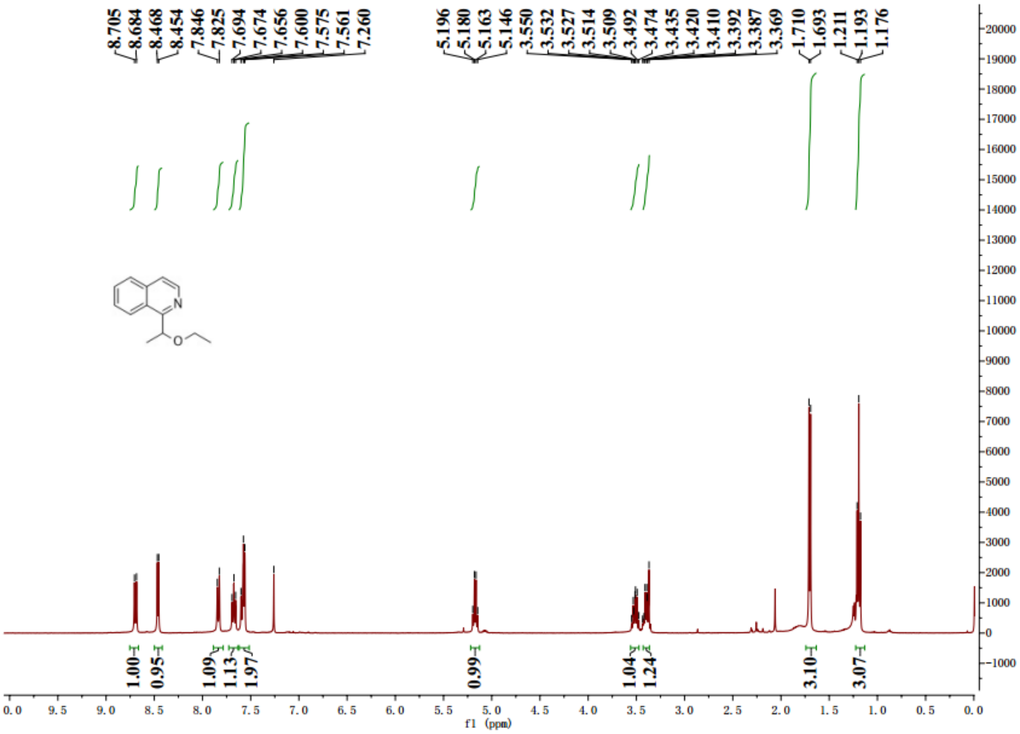
8.705
8.684
8.468
8.454
7.846
7.825
7.694
7.674
7.656
7.600
7.575
7.561
7.260
5.196
5.180
5.163
5.146
3.550
3.532
3.527
3.514
3.509
3.492
3.474
3.435
3.420
3.410
3.392
3.387
3.369
1.710
1.693
1.211
1.193
1.176
N
O
1.00
0.95
1.09
1.13
1.97
0.99
1.04
1.24
3.10
3.07
f1 (ppm)

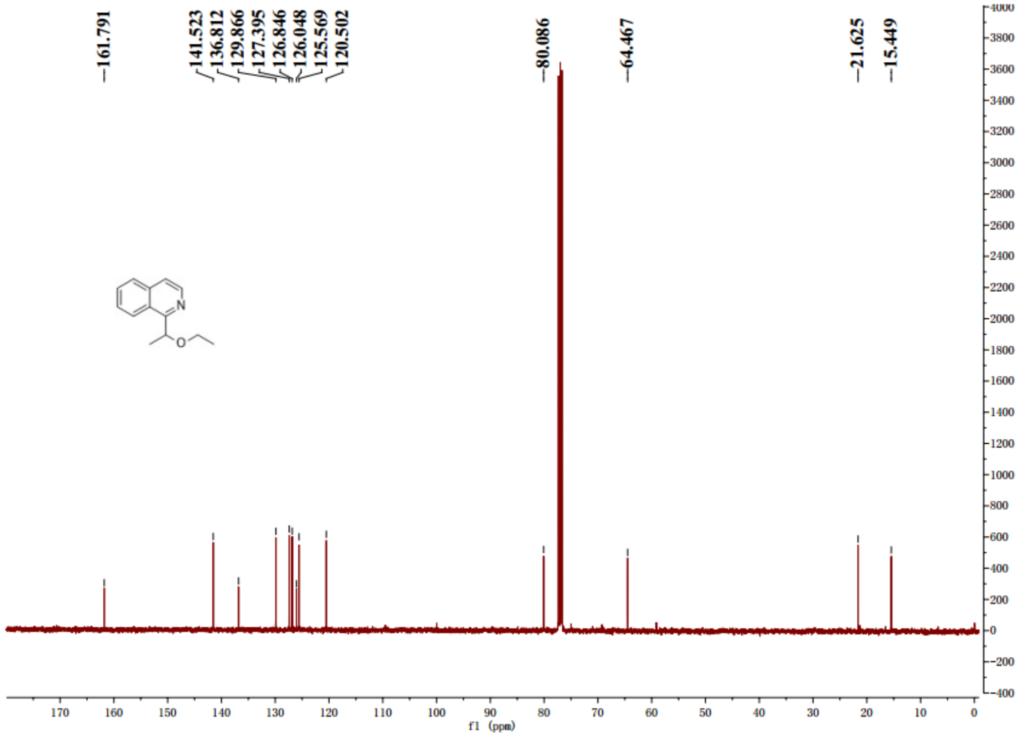
161.791
141.523
136.812
129.866
127.395
126.846
126.048
125.569
120.502
80.086
64.467
21.625
15.449
N
O
f1 (ppm)

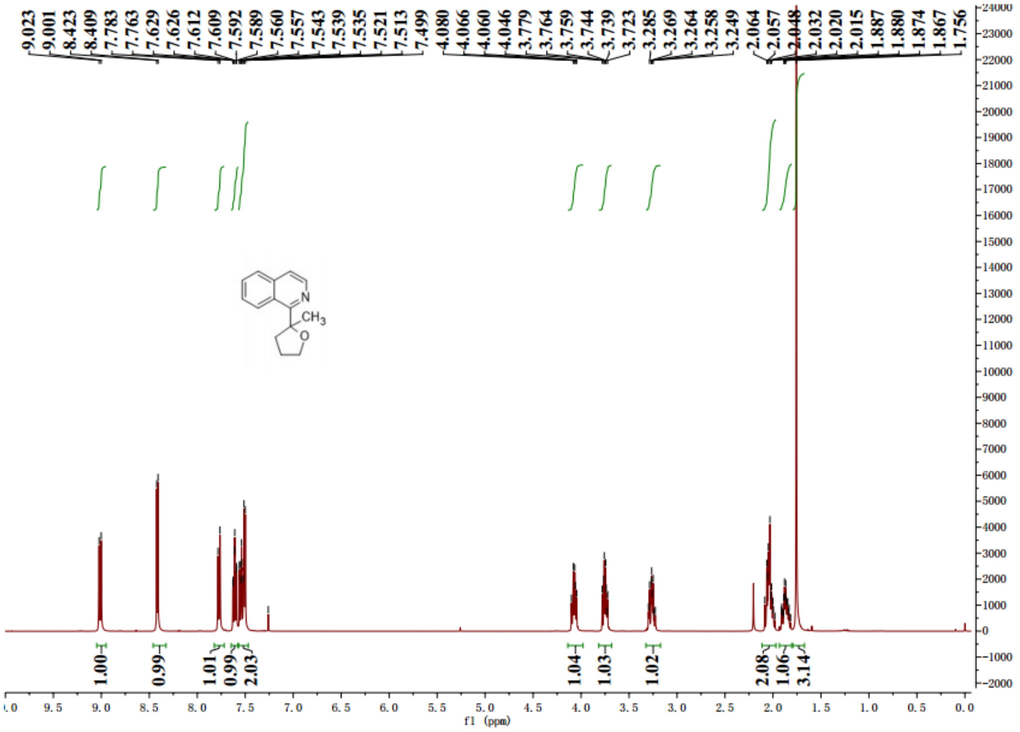

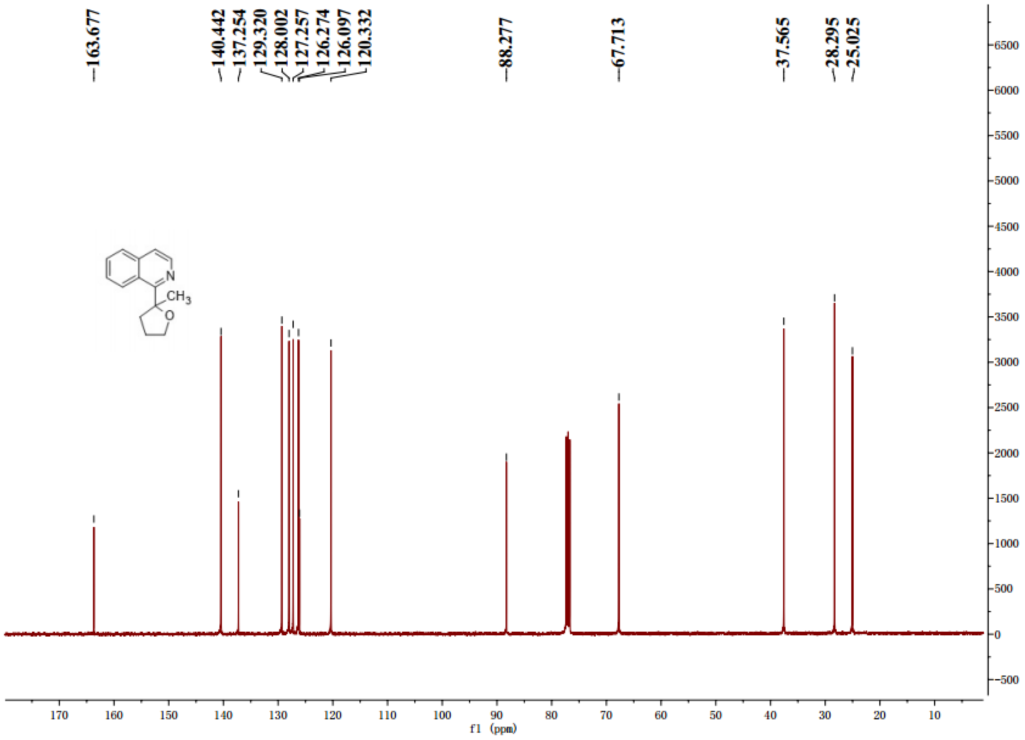

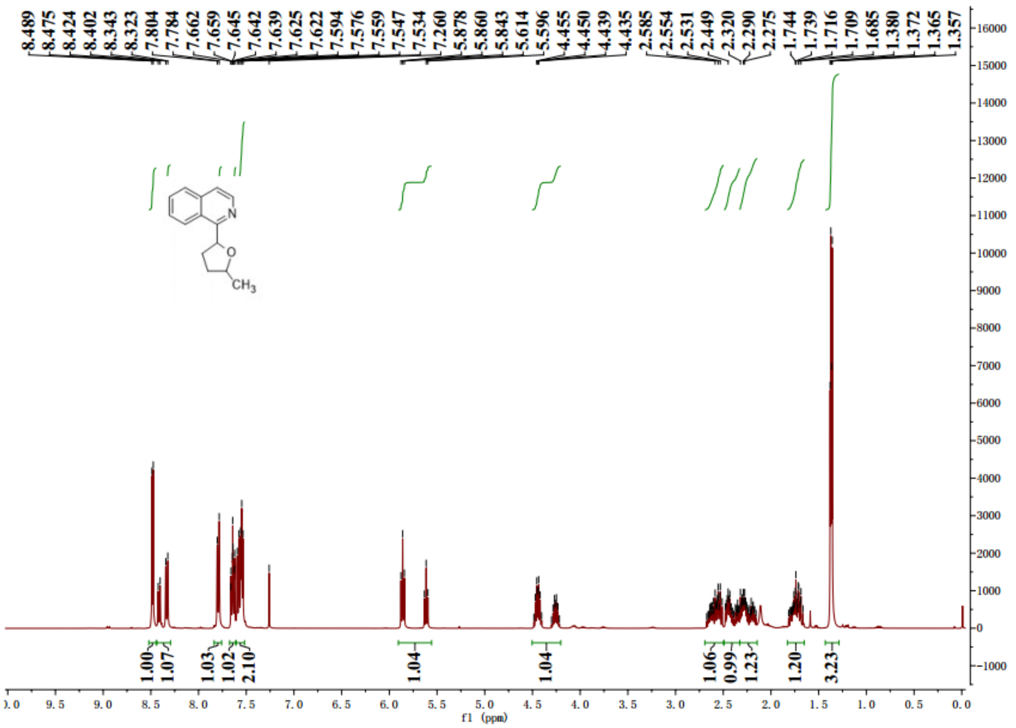
8.489
8.475
8.424
8.402
8.343
8.323
7.804
7.784
7.662
7.659
7.645
7.642
7.639
7.625
7.622
7.594
7.576
7.559
7.547
7.534
7.260
5.878
5.860
5.843
5.614
5.596
4.455
4.450
4.439
4.435
2.585
2.554
2.531
2.449
2.320
2.290
2.275
1.744
1.739
1.716
1.709
1.685
1.380
1.372
1.365
1.357
N
O
CH3
1.00
1.07
1.03
1.02
2.10
1.04
1.04
1.06
0.99
1.23
1.20
3.23
f1 (ppm)

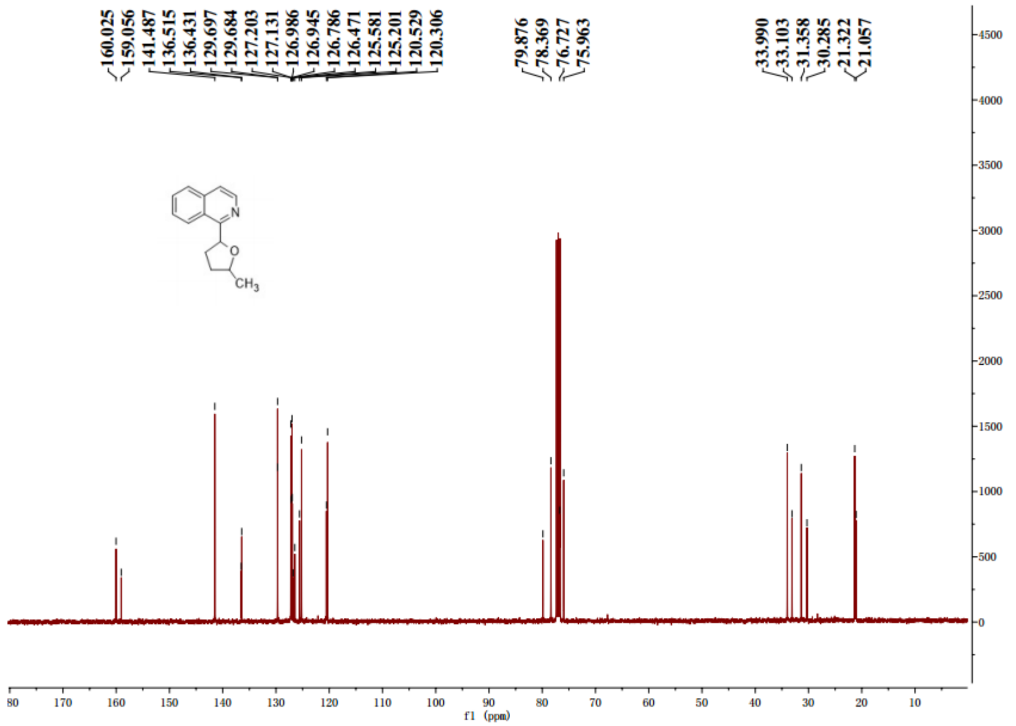
160.025
159.056
141.487
136.515
136.431
129.697
129.684
127.203
127.131
126.986
126.945
126.786
126.471
125.581
125.201
120.529
120.306
79.876
78.369
76.727
75.963
33.990
33.103
31.358
30.285
21.322
21.057
N
O
CH3
f1 (ppm)

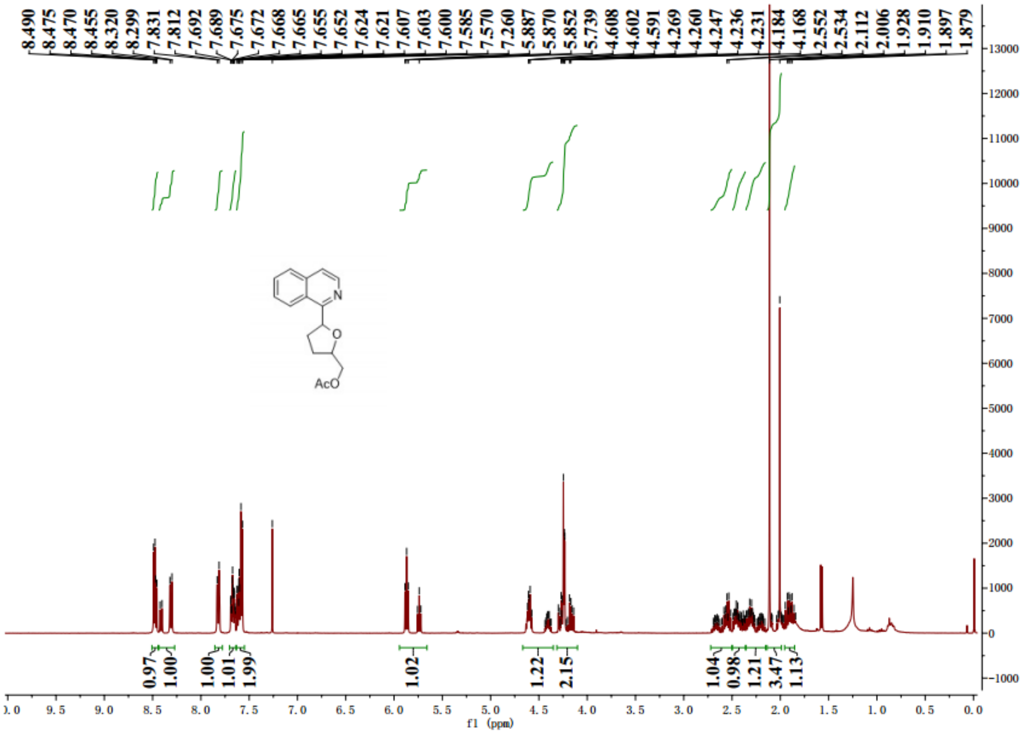

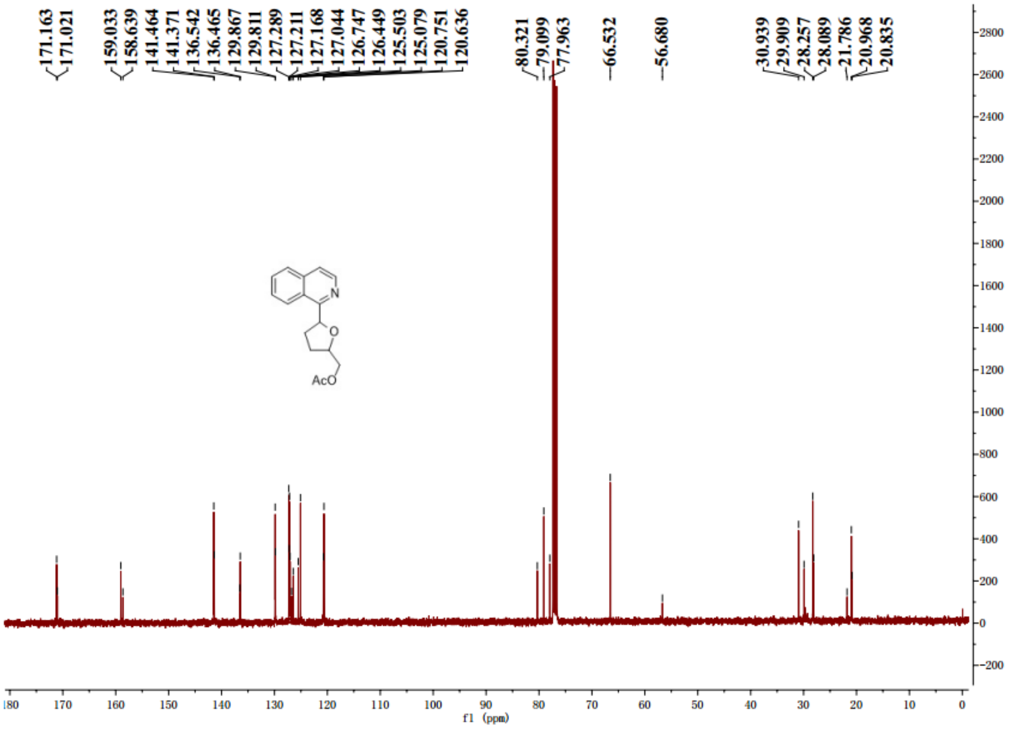

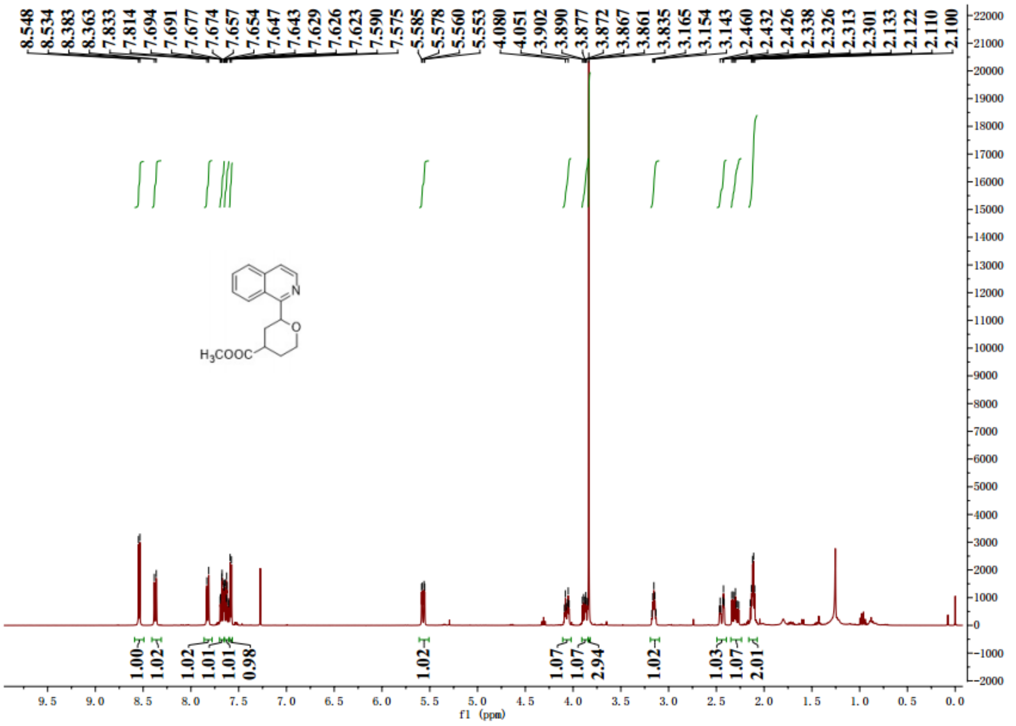
8.548
8.534
8.383
8.363
7.833
7.814
7.694
7.691
7.677
7.674
7.657
7.654
7.647
7.643
7.629
7.626
7.623
7.590
7.575
5.585
5.578
5.560
5.553
4.080
4.051
3.902
3.890
3.877
3.872
3.867
3.861
3.835
3.165
3.154
3.143
2.460
2.432
2.426
2.338
2.326
2.313
2.301
2.133
2.122
2.110
2.100
1.00
1.02
1.02
1.01
1.01
0.98
1.02
1.07
1.07
2.94
1.02
1.03
1.07
2.01
H$_3$COOC
N
O
f1 (ppm)

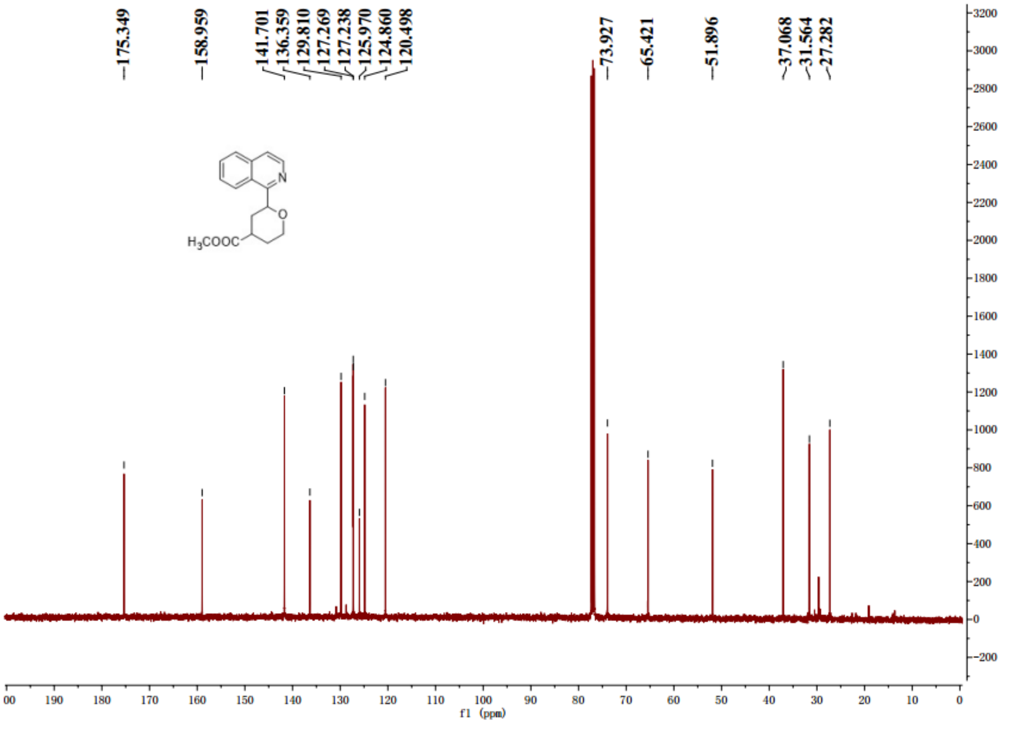
175.349
158.959
141.701
136.359
129.810
127.269
127.238
125.970
124.860
120.498
73.927
65.421
51.896
37.068
31.564
27.282
H$_3$COOC
N
O
f1 (ppm)

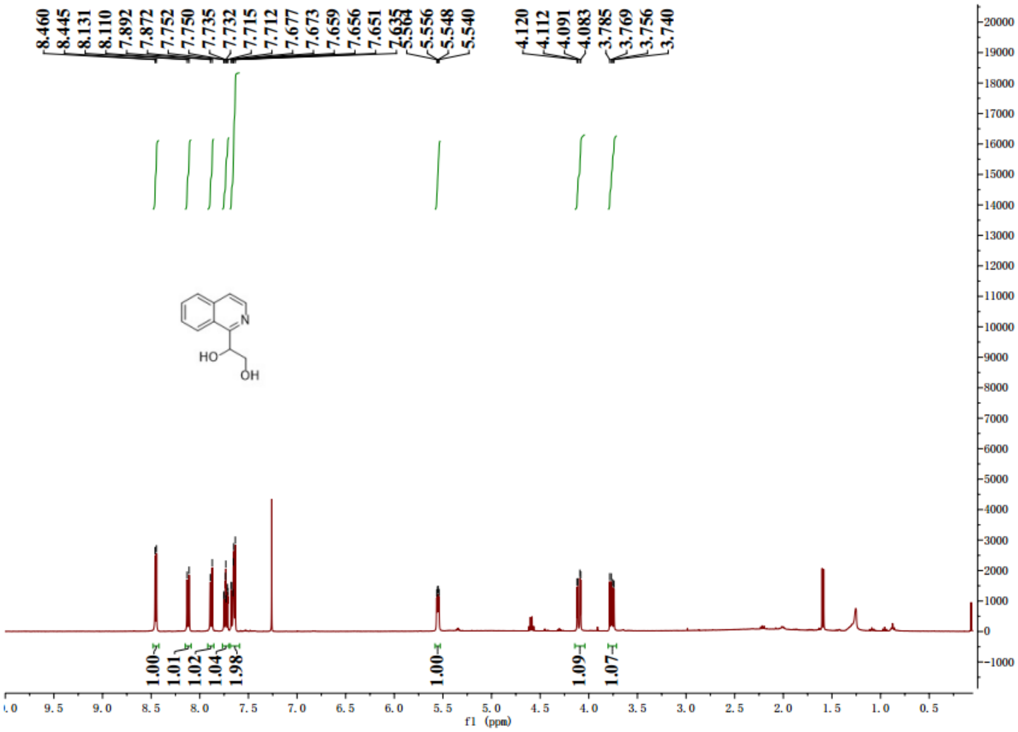

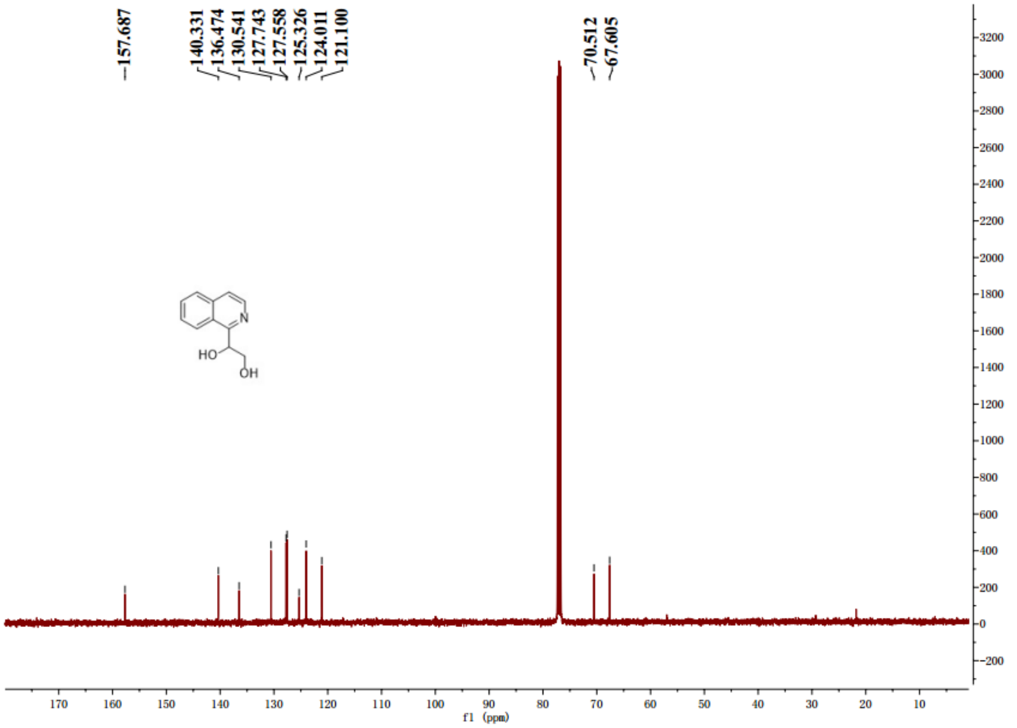

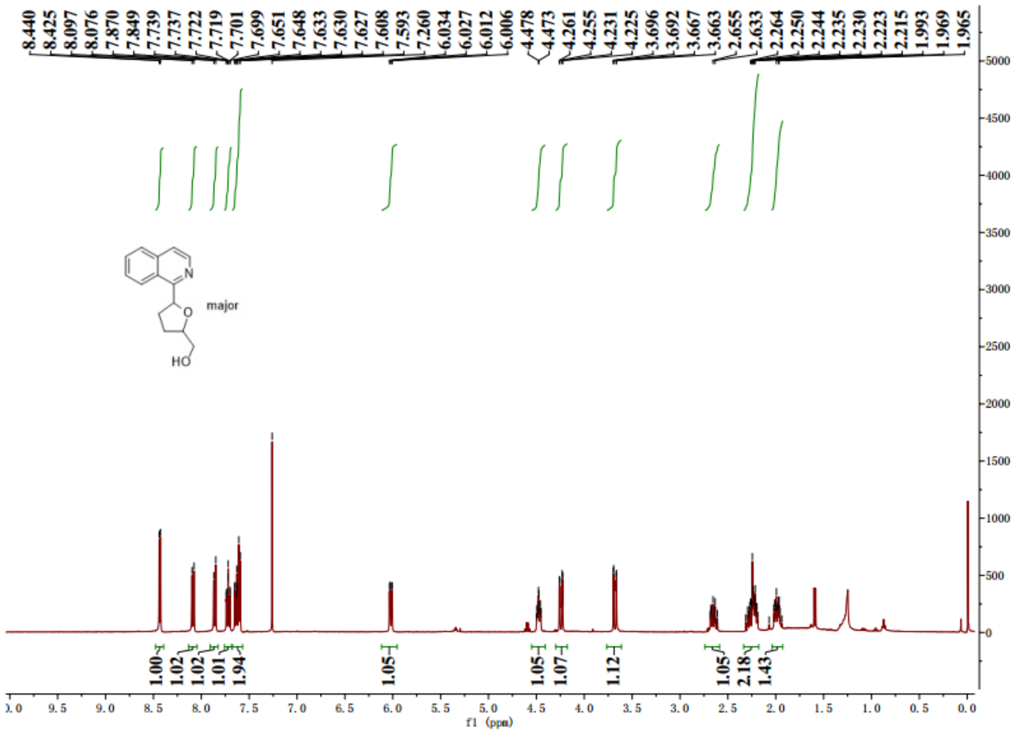

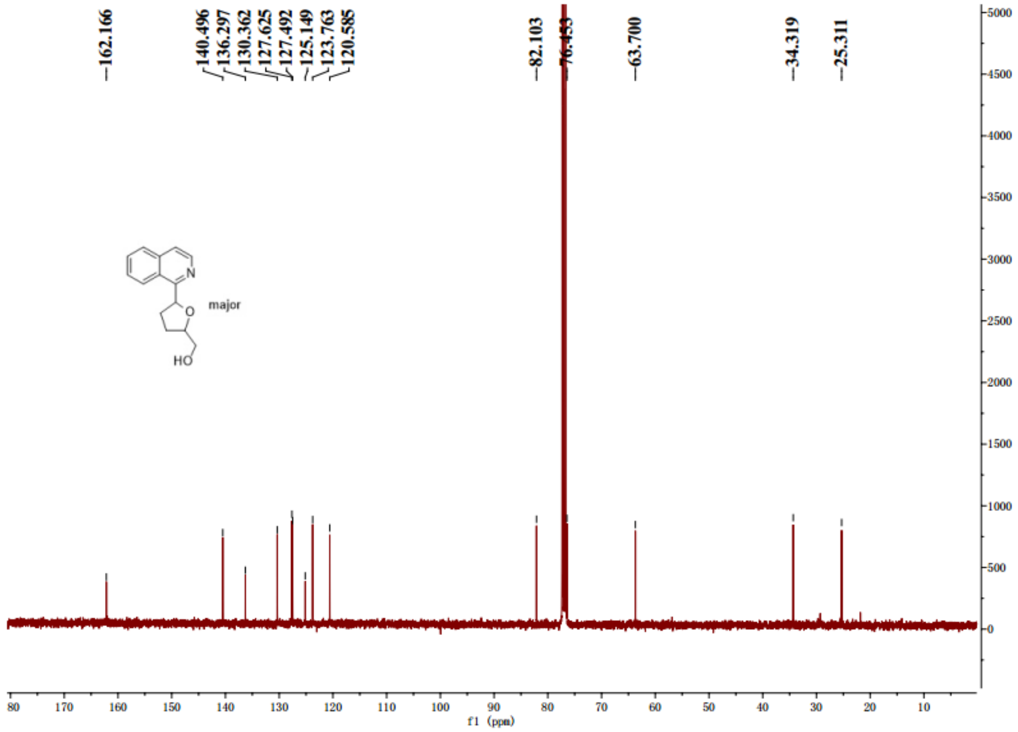

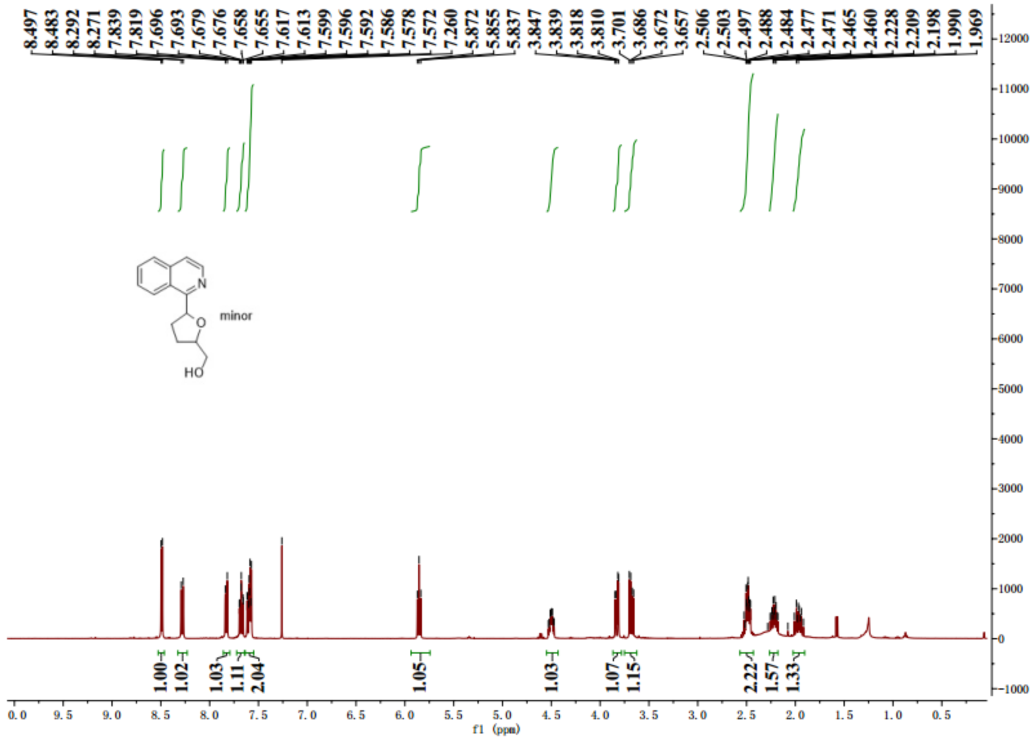

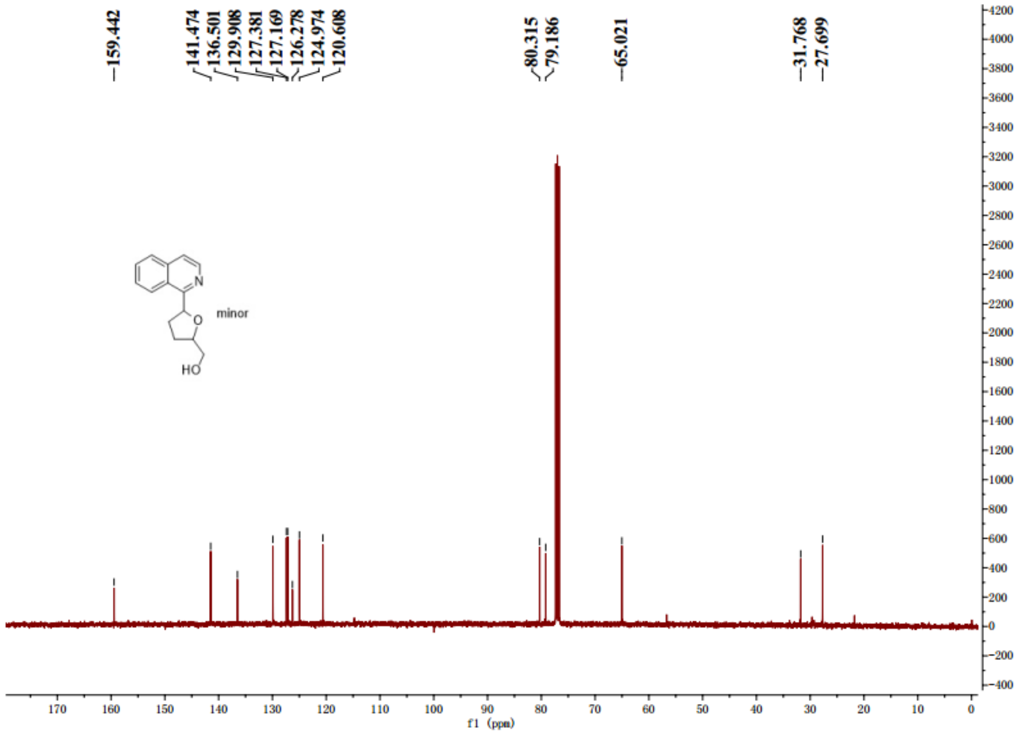

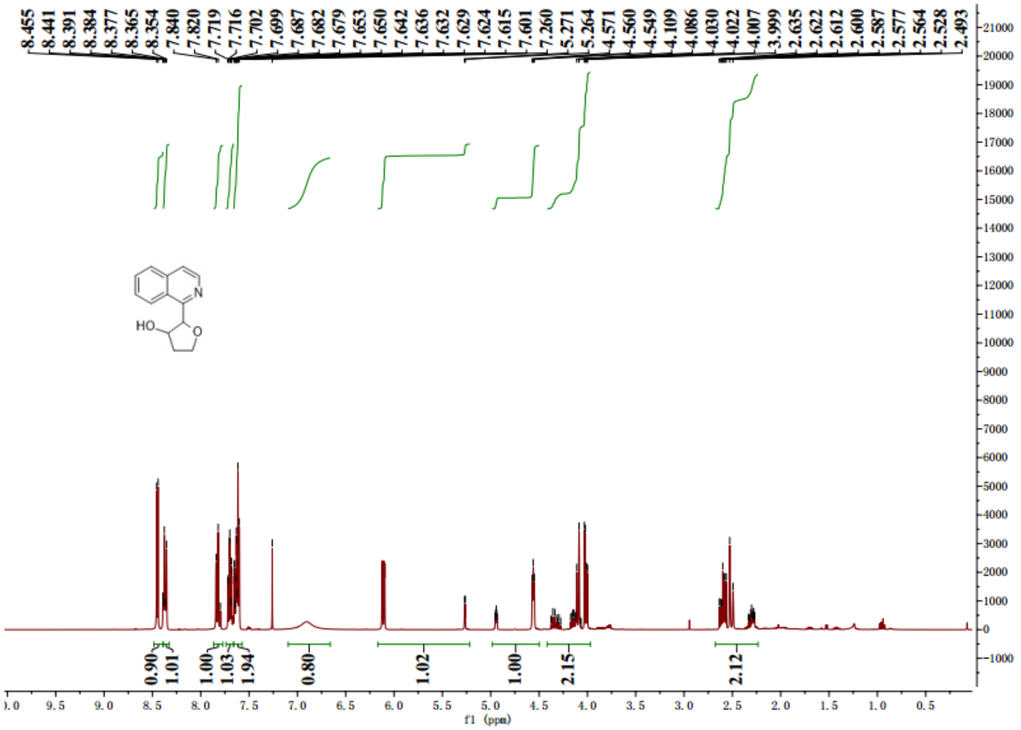

8.455
8.441
8.391
8.384
8.377
8.365
8.354
7.840
7.820
7.719
7.716
7.702
7.699
7.687
7.682
7.679
7.653
7.650
7.642
7.636
7.632
7.629
7.624
7.615
7.601
7.260
5.271
5.264
4.571
4.560
4.549
4.109
4.086
4.030
4.022
4.007
3.999
2.635
2.622
2.612
2.600
2.587
2.577
2.564
2.528
2.493
HO
O
N
0.90
1.01
1.00
1.03
1.94
0.80
1.02
1.00
2.15
2.12
f1 (ppm)

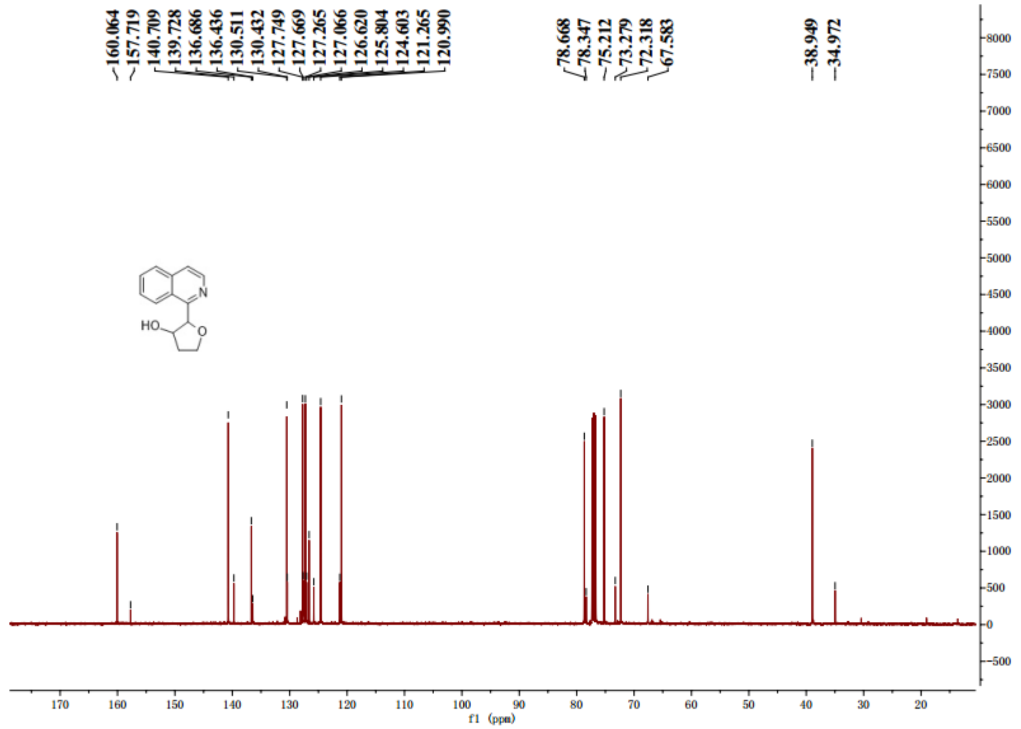

160.064
157.719
140.709
139.728
136.686
136.436
130.511
130.432
127.749
127.669
127.265
127.066
126.620
125.804
124.603
121.265
120.990
78.668
78.347
75.212
73.279
72.318
67.583
38.949
34.972
HO
O
N
f1 (ppm)

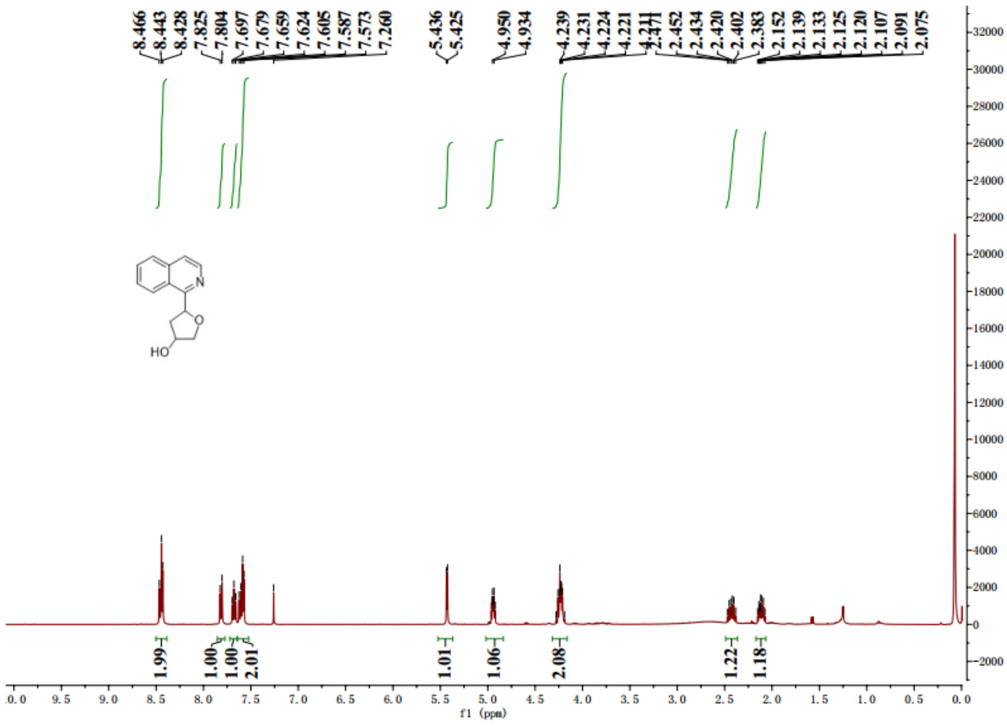

8.466
8.443
8.428
7.825
7.804
7.697
7.679
7.659
7.624
7.605
7.587
7.573
7.260
5.436
5.425
4.950
4.934
4.239
4.231
4.224
4.221
4.211
2.471
2.452
2.434
2.420
2.402
2.383
2.152
2.139
2.133
2.125
2.120
2.107
2.091
2.075
HO
O
N
1.99
1.00
1.00
2.01
1.01
1.06
2.08
1.22
1.18
f1 (ppm)

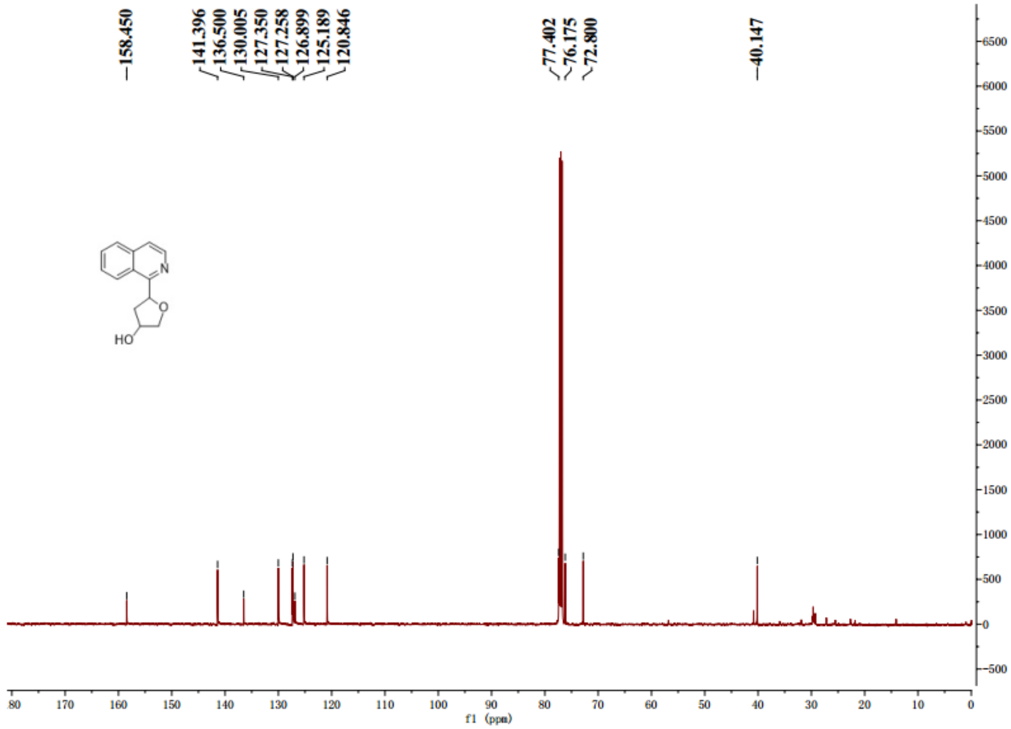

158.450
141.396
136.500
130.005
127.350
127.258
126.899
125.189
120.846
77.402
76.175
72.800
40.147
HO
O
N
f1 (ppm)

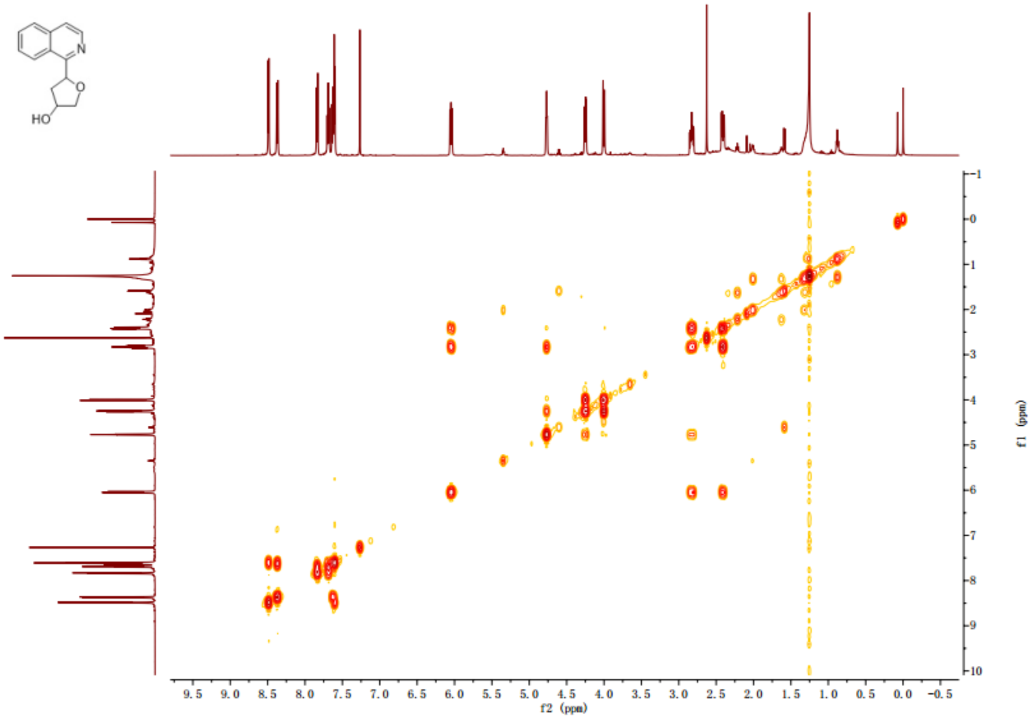

HO
N
O
f2 (ppm)
f1 (ppm)

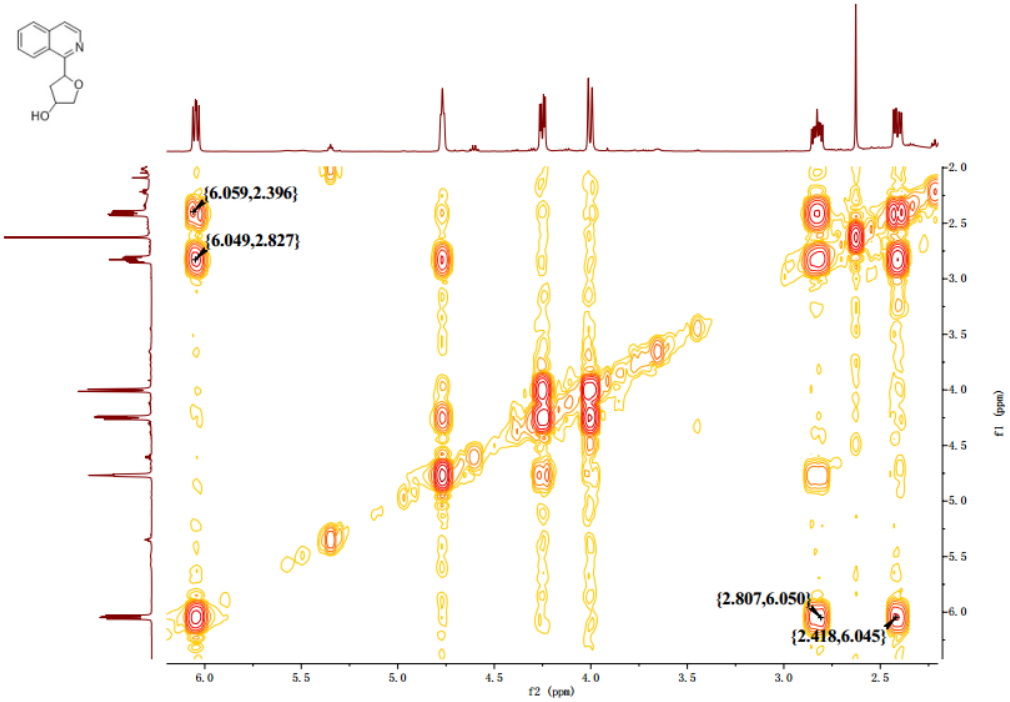

HO
N
O
{6.059,2.396}
{6.049,2.827}
{2.807,6.050}
{2.418,6.045}
f2 (ppm)
f1 (ppm)

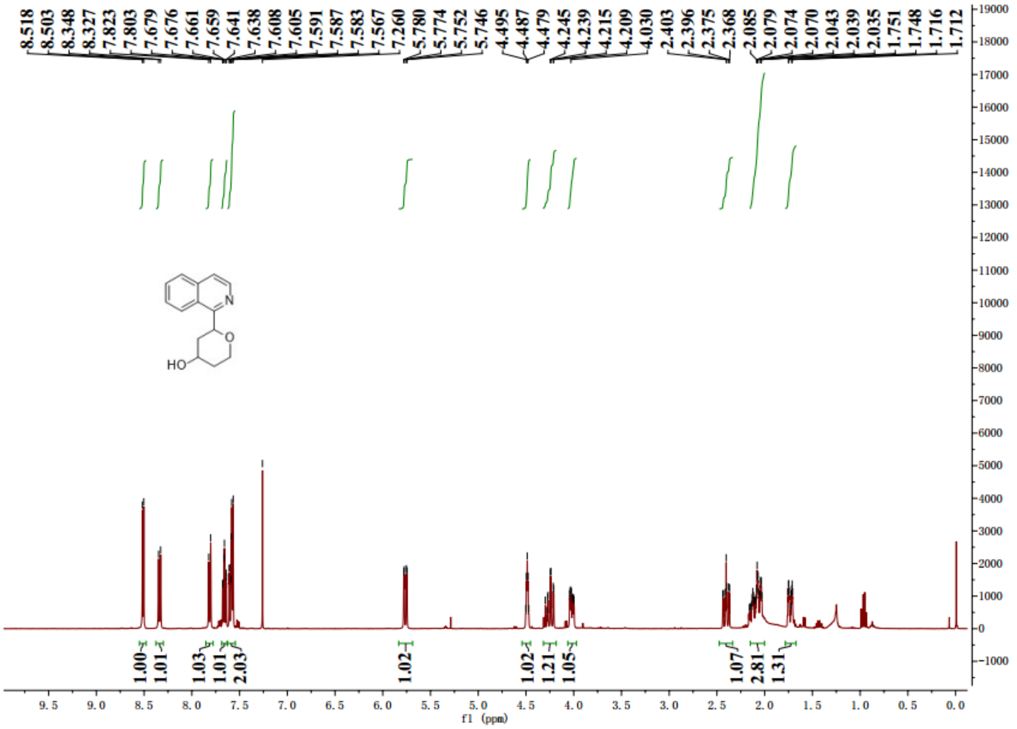
8.518
8.503
8.348
8.327
7.823
7.803
7.679
7.676
7.661
7.659
7.641
7.638
7.608
7.605
7.591
7.587
7.583
7.567
7.260
5.780
5.774
5.752
5.746
4.495
4.487
4.479
4.245
4.239
4.215
4.209
4.030
2.403
2.396
2.375
2.368
2.085
2.079
2.074
2.070
2.043
2.039
2.035
1.751
1.748
1.716
1.712
HO
N
O
1.00
1.01
1.03
1.01
2.03
1.02
1.02
1.21
1.05
1.07
2.81
1.31
f1 (ppm)

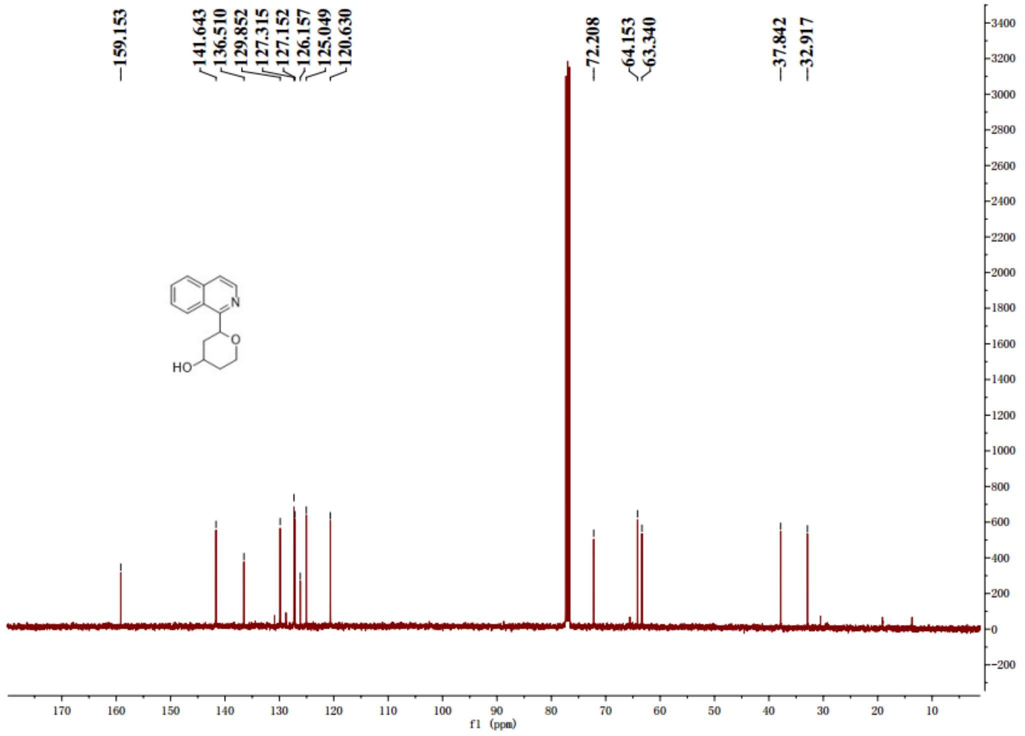
159.153
141.643
136.510
129.852
127.315
127.152
126.157
125.049
120.630
72.208
64.153
63.340
37.842
32.917
HO
N
O
f1 (ppm)

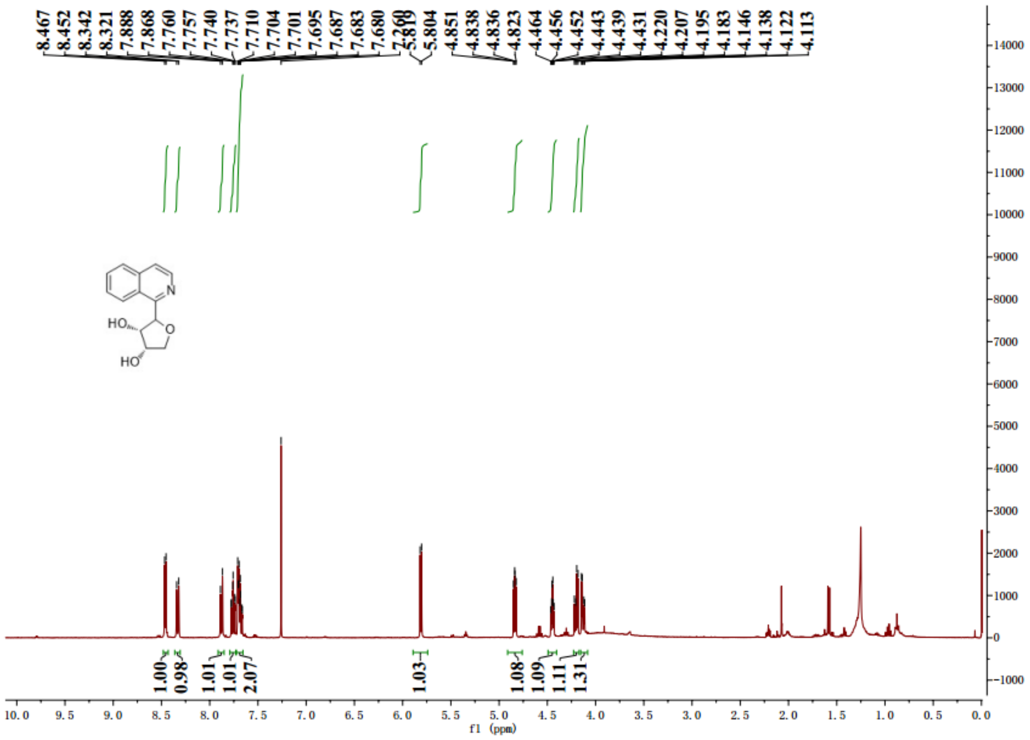

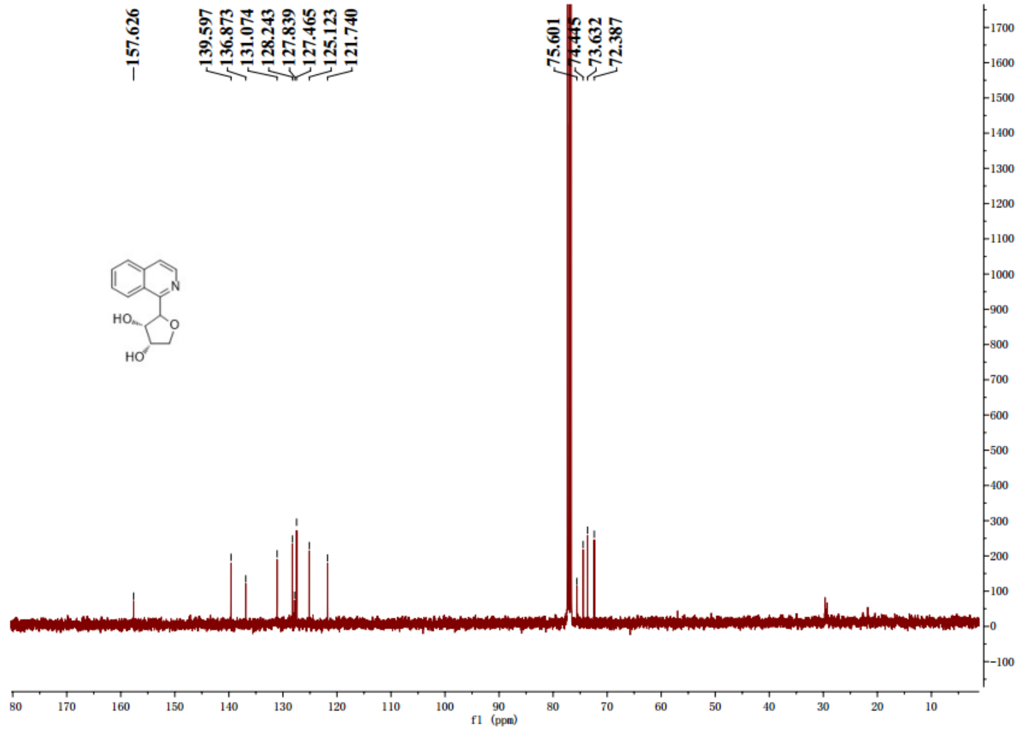

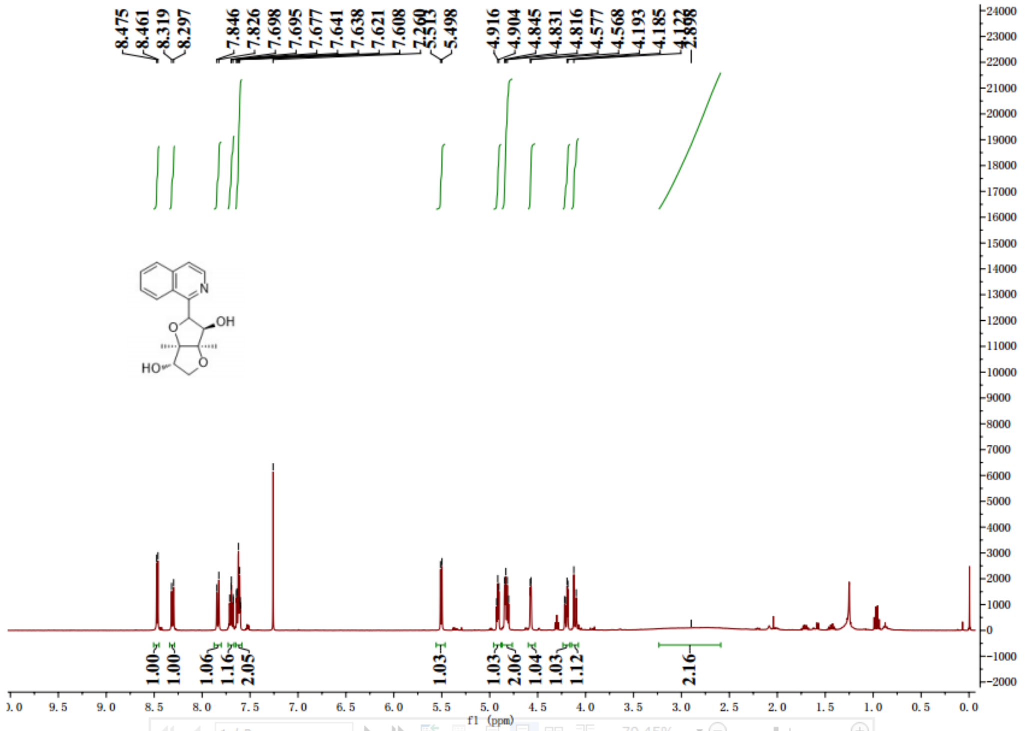

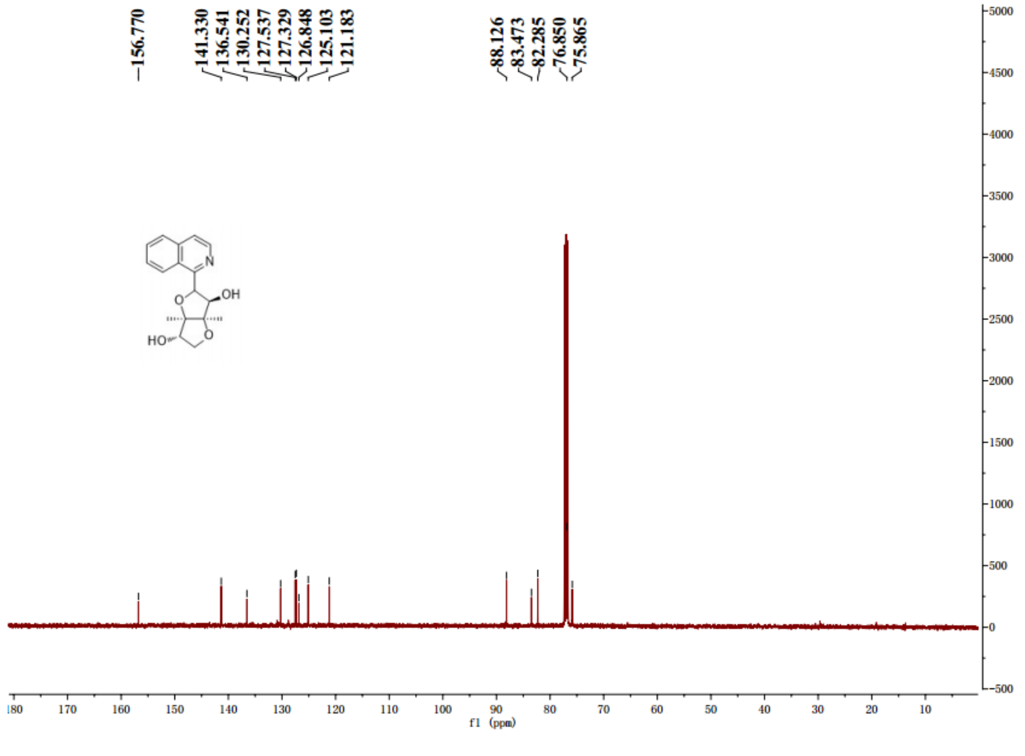

Supplement: Supplementary file 1 [file SC-008-C6SC05697K-s001.pdf]
